# Gap‐Free *Cannabis sativa* Assemblies Reveal Post‐Polyploid Genome Remodelling and Refine the Evolution of Cannabinoid Synthase Genes

**DOI:** 10.1111/pbi.70766

**Published:** 2026-10-22

**Authors:** Jiangjiang Zhang, Chuchu Qin, Zhao Li, Lining Zhao, Ashok Biswas, Touming Liu, Mingbao Luan, Lin Li, Defang Li, Jianbin Yan

**Affiliations:** ^1^ Shenzhen Branch, Guangdong Laboratory for Lingnan Modern Agriculture, Key Laboratory of Synthetic Biology, Ministry of Agriculture and Rural Affairs, Advanced Synthetic Biology Institute at Hohhot, Agricultural Genomics Institute at Shenzhen Chinese Academy of Agricultural Sciences Shenzhen China; ^2^ Institute of Bast Fiber Crops and Center for Southern Economic Crops Chinese Academy of Agricultural Sciences Changsha China; ^3^ National Key Laboratory of Crop Genetic Improvement Huazhong Agricultural University Wuhan China; ^4^ Key Laboratory of Biobreeding for Specialty Horticultural Crops of Jiangsu Province, College of Horticulture and Landscape Architecture Yangzhou University Yangzhou China

**Keywords:** cannabidiol, cannabinoid biosynthesis genes, *Cannabis sativa*, hemp, LTR retrotransposons, nature product, whole‐genome triplication

## Abstract

Despite the therapeutic importance of cannabinoids, the scarcity of gap‐free *Cannabis* genomes has limited the dissection of the genetic and genomic basis of cannabinoid biosynthesis and genome evolution. Here, we generated two haplotype‐phased, gap‐free, gold‐standard assemblies and used them to investigate post‐polyploid genome remodelling and refine the evolutionary history of cannabinoid synthase genes. Analyses of these gap‐free assemblies recovered a *γ* whole‐genome triplication (WGT) signal shared with core eudicots and provided no evidence for additional lineage‐specific whole‐genome duplication (WGD) in *Cannabis*. The extant *Cannabis* genome reflects long‐term retention and divergence of a subset of *γ*‐derived duplicates, together with subsequent non‐WGD‐derived gene duplications and repeat‐associated remodelling that contributed substantially to present‐day genome diversification. Furthermore, phylogenomic, structural and syntenic evidence indicate that a precursor duplication predating the *Cannabis*–*Humulus* split gave rise to two lineages retained in *Cannabis* on chromosomes 7 and 2. In *Cannabis*, the chromosome 7 lineage subsequently expanded and diversified into the functional cannabinoid synthase genes, whereas *Humulus retained* only the lineage resembling the *Cannabis* chromosome 2 copy. This asymmetric retention and diversification provide new insights into the origin and specialization of cannabinoid biosynthesis in *Cannabis*. Together, these results provide a high‐quality genomic framework for understanding the history of polyploidy, the evolution of the cannabinoid synthase gene and genome diversification in *Cannabis*.

## 1 Introduction

*Cannabis sativa* is a multipurpose crop with rapidly expanding global cultivation. It provides fibre, seed oil, protein and dietary supplements, and plays an increasingly important role in medicine and wellness (van Bakel et al. 2011). The expanding medical, agricultural and commercial value of *Cannabis* has driven increasing interest in its biology, domestication and genome evolution (Xie et al. 2023). *Cannabis* also represents a powerful system for studying evolution and diversification, shaped by both natural environmental pressures and prolonged human influence (Ren et al. 2021; Rull 2022; Wee et al. 2025; Zhang et al. 2018). Its exceptional environmental adaptability is reflected in a broad geographic range spanning Europe, China and North America (Wartenberg et al. 2021), and wild germplasm collections reveal substantial variation in traits such as seed weight and responses to drought and ultraviolet‐B radiation (Rodriguez‐Morrison et al. 2021; Welling et al. 2016, 2018). Complementing this natural selection, *Cannabis* has one of the oldest and most intensive histories of anthropogenic domestication, dating back to the Neolithic period in East Asia, where humans imposed strong and multidirectional selection for fibre, food (oil), medicine and psychoactive compounds (van Bakel et al. 2011). This combined action of environmental and human‐mediated selection has sculpted a genome with high heterozygosity, numerous polymorphic loci and distinct population structure, leaving detectable signatures of selective sweeps on specific chromosomes (Lynch et al. 2025; Ren et al. 2021).

The growing recognition of the medicinal potential of *Cannabis* has focused pharmacological interest on three primary cannabinoids: Δ^9^‐tetrahydrocannabinol (Δ^9^‐THC), cannabidiol (CBD) and cannabigerol (CBG). Δ^9^‐THC is the main psychoactive constituent and has analgesic, antiemetic and anti‐inflammatory effects (McPartland et al. 2017; Rock et al. 2021). CBD is non‐intoxicating and has been investigated for its potential therapeutic applications in conditions such as glaucoma, epilepsy, and depression (Kim et al. 2023). CBG, another non‐psychoactive cannabinoid, is emerging as a candidate therapy for neurodegenerative disorders, inflammatory bowel disease and neuropathic pain (Li et al. 2024). To better describe chemical variation among cultivars, modern nomenclature and regulatory frameworks classify *Cannabis* cultivars into chemotypes based on predominant cannabinoid content: Type I (Δ^9^‐THC–dominant), Type II (approximately 1:1 THC: CBD), Type III (CBD–dominant), and Type IV (CBG–dominant), with further characterization into chemovars defined by terpene profiles such as myrcene, limonene and caryophyllene (Adhikary et al. 2021; Cui et al. 2022; Hillig and Mahlberg 2004; Lewis et al. 2018; Li 1973; McPartland and Small 2020; Sakamoto et al. 1998; Simiyu et al. 2022; Smith et al. 2022; Visković et al. 2023).

The core enzymatic steps of the cannabinoid biosynthetic pathway have been elucidated. Olivetol synthase (*OLS*) catalyses the stepwise condensation of hexanoyl‐CoA to generate a polyketide intermediate, which is then cyclized and aromatized by olivetolic acid cyclase (*OAC*) to form olivetolic acid (OA). An aromatic prenyltransferase (*aPT4*) then prenylates OA at the 2‐resorcinol position, producing cannabigerolic acid (CBGA), the central branch point of the pathway. CBGA is subsequently oxidocyclized by cannabidiolic acid synthase (*CBDAS*), Δ^9^‐tetrahydrocannabinolic acid synthase (*THCAS*), or cannabichromenic acid synthase (*CBCAS*) to yield CBDA, THCA and CBCA, which decarboxylate spontaneously to their neutral forms (CBD, THC and CBC) (Luo et al. 2019). Previous research reported higher expression of *THCAS* and *CBDAS* in the drug‐type cultivar Purple Kush (PK) than in the fibre‐type cultivar Finola (van Bakel et al. 2011). Further analyses showed that the *THCAS* and *CBDAS* loci reside in repeat‐rich, low‐recombination regions with extensive genomic rearrangements and that they occupy distinct chromosomal regions in PK and Finola, with high structural divergence between the cultivars (Laverty et al. 2019). In the high‐CBD cultivar Cs10 (CBDRx), which has predominantly marijuana ancestry, the genome harbours hemp‐derived *CBDAS* introgression but lacks an intact *THCAS* gene sequence, confirming that *CBDAS* and *THCAS* map to chromosome 7 (hereafter Chr7; ‘Chr’ denotes chromosome) within highly repetitive regions (Grassa et al. 2021). A *Cannabis* pangenome constructed from 193 assemblies further revealed that cannabinoid synthase genes are embedded in structurally dynamic regions that nonetheless exhibit unusually low local genetic diversity and that *THCAS* and *CBDAS* typically map within 5 Mb on Chr7 (Lynch et al. 2025).

Over the past decade, genome‐scale studies of *Cannabis sativa* have expanded rapidly, paralleling the growth of the global cannabis industry. Multiple de novo genome assemblies now achieve chromosome‐scale continuity across diverse germplasm types, including Purple Kush (PK), Cannbio‐2, the CBD‐type cultivars Cs10 (CBDRx) and Pink Pepper (Pink), the hemp‐type cultivar Finola, the seed cultivar Yushe, and the wild accession JL (van Bakel et al. 2011; Braich et al. 2020; Gao et al. 2020; Grassa et al. 2021; Hurgobin et al. 2021; Laverty et al. 2019; Ryu et al. 2024; Wei et al. 2023). The dioecious nature of *Cannabis*, together with asynchronous flowering and high outcrossing rates (Punja and Holmes 2020; Steel et al. 2023), has contributed to high heterozygosity and abundant repetitive content, which in turn complicates assembly and scaffolding. As a result, most early *Cannabis* assemblies still contained gaps, and some were scaffolded using reference‐guided approaches rather than independent Hi‐C data. These limitations have contributed to inconsistencies in chromosome orientation, sequence ordering, and the resolution of highly repetitive regions across assemblies, particularly in cannabinoid synthase genes on Chr7 (van Bakel et al. 2011; Braich et al. 2020; Gao et al. 2020; Grassa et al. 2021; Hurgobin et al. 2021; Laverty et al. 2019; Ryu et al. 2024; Wei et al. 2023). In addition, many early genome resources were derived from a limited set of Western cultivars and therefore did not adequately represent global germplasm diversity (Lynch et al. 2025). Although a recent pangenome has begun to address this limitation, it has also highlighted that worldwide diversity remains undersampled, with Asian germplasm notably underrepresented (Lynch et al. 2025).

Despite these advances, several key questions remain incompletely resolved. Earlier analyses of synonymous substitution rate distributions and intragenomic synteny in *Cannabis* assemblies were interpreted as evidence for one or two additional whole‐genome duplication events beyond the *γ* triplication shared with core eudicots (Gao et al. 2020; Wei et al. 2023). More recently, a *Cannabis* pangenome study suggested that much of the apparent recent duplication signal may instead reflect transposable elements and supported the absence of additional lineage‐specific whole‐genome duplications. Whether this conclusion can be independently recovered from gap‐free, haplotype‐phased assemblies without explicit TE masking remains unclear. In parallel, previous studies have proposed an evolutionary framework for cannabinoid synthase genes within the broader berberine bridge enzyme–like (BBE‐like) oxidoreductase family, but finer‐scale aspects of their evolutionary history remain unresolved, including their detailed relationships to closely related BBE‐like homologues in *Cannabis* and its allied taxa (Daniel et al. 2017; Van Velzen and Schranz 2021). In addition, the highly repetitive, heterozygous nature of the *Cannabis* genome, together with the localization of synthase genes in long terminal repeat (LTR) retrotransposon‐rich, low‐recombination regions, has made it difficult to resolve copy number variation, local genome structure, and cis‐regulatory organization at these loci, as well as the potential effects of transposable element insertions on cannabinoid pathway gene expression. More broadly, how LTR‐RT expansion and the retention and divergence of duplicated genes have contributed to the structural evolution of *Cannabis* also remains unclear.

To address these limitations, we generated two gap‐free, haplotype‐phased, chromosome‐level assemblies for a single high‐CBDA‐content *Cannabis* accession of Asian origin cultivated in Changsha, China. We examined the structure of putative centromeric regions and reassessed the duplication history of the *Cannabis* genome within a gap‐free assembly framework. Building on previous models of cannabinoid oxidocyclase evolution (Van Velzen and Schranz 2021), we further investigated the evolutionary history of cannabinoid synthase genes, including their relationship to BBE‐like precursors and the structural diversification of the Chr7 synthase region. We also characterized LTR retrotransposon diversity and duplicate‐gene repertoires across multiple *Cannabis* assemblies to investigate how repeat variation and duplicated‐gene retention and divergence have contributed to genome evolution and the present genomic landscape. In addition, we examined cannabinoid pathway gene expression across accessions and explored its relationship with transposable element insertions. Together, this work provides a high‐quality, gap‐free genomic resource for studying *Cannabis* genome evolution and cannabinoid biology and expands the genomic representation of Asian‐origin *Cannabis* in comparative analyses.

## 2 Results

### 2.1 Gap‐Free Assembly and Annotation of the Golden *Cannabis sativa* Haplotypes

The *Cannabis* genome is structurally complex, and many gaps in currently available assemblies remain difficult to resolve. In total, we generated 40.37 Gb of PacBio HiFi reads (54× coverage, mean read length 15.52 kb), 102 Gb of PacBio CLR reads (137.80× coverage, mean read length 25.80 kb), and 82.26 Gb of BGI (Beijing Genomics Institute) Hi‐C reads (111.68× coverage, mean read length 300 bp) (Table S1). Based on k‐mer frequency spectra, the genome size was estimated at ~736 Mb (Figure S1a,b; Tables S2 and S5). To generate a gap‐free assembly, we combined PacBio high‐fidelity (HiFi), PacBio continuous long reads (CLR) and BGI Hi‐C data and developed a Graph‐based Gap‐filling guided by Unanchored Reads (GGUR) strategy, in which graph paths derived from CLR reads were integrated with Hi‐C‐supported phased backbones and subsequently validated by Hi‐C contact patterns and read mapping (Figure 1a–d; Figure S1c,d; Tables S3 and S4). Using GGUR, we assembled two gap‐free, haplotype‐phased *Cannabis* assemblies with sizes of 741.03 Mb and 744.89 Mb, which closely matched the genome survey estimates (Tables S2 and S5). We refer to this phased assembly set as Golden *Cannabis sativa* (GCS), with the two haplotypes designated GCS_Hap1 and GCS_Hap2 (hereafter Hap1 and Hap2). Here, Hap1 and Hap2 are used as identifiers for the two phased assemblies and do not imply chromosome‐wide biological haplotype identity across the genome.

**FIGURE 1:**
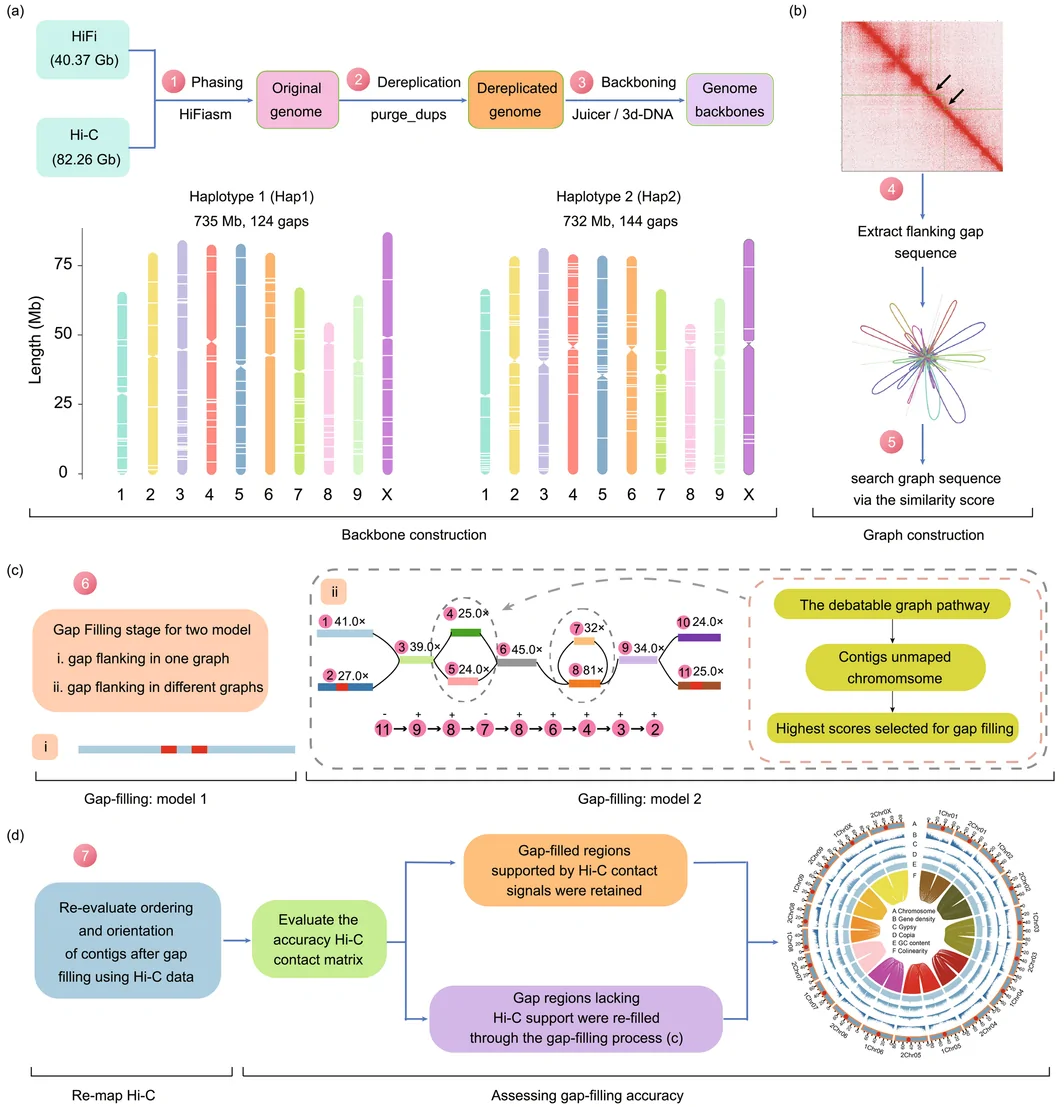
Assembly and graph‐based gap‐filling workflow for the *Cannabis sativa* genome. (a) Construction of two haplotype‐phased assembly backbones (Hap1 and Hap2) from HiFi and Hi‐C data. White marks indicate residual gap locations along each chromosome. Chromosomes are colour‐coded for clarity. (b) Construction of a CLR‐based sequence graph for gap filling. Gap‐flanking sequences from the draft backbones, together with Hi‐C contact information, were used to query the graph and identify candidate paths spanning each gap. (c) Two gap‐filling models were used. In model 1, both flanking sequences of a gap were located within a single graph context, and the gap was resolved by tracing a continuous graph path between them. In model 2, the two flanking sequences were associated with different graph branches or contigs, and the gap was resolved by searching candidate bridging paths among disputed graph connections using unanchored sequences from the initial assembly and selecting the highest‐scoring match for gap filling. When graph loops were present, node depth was used as auxiliary evidence to evaluate the candidate path. The lower schematic illustrates the contig‐connection graph used to recover the filling sequence. ‘+’ and ‘−’ indicate the forward and reverse strands, respectively. (d) After gap filling, contig order and orientation were re‐evaluated using Hi‐C data. Regions supported by the Hi‐C contact map were retained, whereas unsupported regions were subjected to an additional round of graph‐based gap filling as in panel (d). The Circos plot summarizes the final assemblies and their major genomic features.

Both haplotypes show high assembly quality. The completeness of Benchmarking Universal Single‐Copy Orthologs (BUSCO) reached 99.40% for Hap1 and 99.30% for Hap2 (Tables S6 and S7). The assemblies also showed high contiguity, with a contig N50 length of 78.94 Mb, and both reached gold‐standard quality under LAI classification, with LAI values of 23.54 and 23.24, respectively. Genome quality values (QV) were 58.33 for Hap1 and 59.37 for Hap2 (Table 1). Whole‐genome structural comparisons further supported high consistency between the two haplotypes in overall TE landscape and Hi‐C contact patterns (Figure S1e,f).

**TABLE 1:** Assembly and annotation features of the two haplotype‐phased assemblies of GCS (Golden *Cannabis sativa* ).

Genome features	Haplotype 1	Haplotype 2
Genome size (bp)	741 035 395	744 889 911
The shortest chromosome (bp)	54 165 230	55 107 759
The longest chromosome (bp)	86 241 464	86 244 503
Average length (bp)	74 103 540	74 488 991
Gap number	0	0
N50 length (contigs, scaffold) (bp)	78 935 160	79 915 473
GC content (%)	34.13	34.13
LAI value	23.54	23.24
Scaffold number	10	10
Repeat region size (bp)	581 968 272	584 892 678
Repeat percentage (%)	78.53	78.52
BUSCOs(C) (%)	99.40	99.30
QV value	58.33	59.37
Predicted genes	27 365	27 776
Exon number	5.074	5.175
Average gene length (bp)	2772	2976
Average coding sequence length (bp)	1117	1129
Average intron length (bp)	1655	1847

A total of 27 365 and 27 776 genes were annotated in the two assemblies, respectively (Table 1), of which 25 813 and 26 332 were functionally assigned based on multiple databases, corresponding to annotation rates of 94.33% and 94.80% (Figure S2a–c). Canonical telomeric repeats (TTTAGGG/CCCTAAA) were identified at multiple chromosome ends in both haplotypes, consistent with the high continuity of the assemblies (Table S8). We also mapped 45S and 5S rDNA arrays to multiple chromosomes. In both haplotypes, the major 45S nucleolar organizer region was located on Chr7 and formed the largest rDNA tract (~0.49 Mb in Hap1 and ~0.57 Mb in Hap2), whereas 5S arrays were more numerous and more broadly dispersed across the genome (Tables S9 and S10).

Independent validation further supported the accuracy and completeness of the assemblies. Sequencing reads from all three platforms mapped back to the assemblies at rates exceeding 99% (Tables S11–S13), and summaries in non‐overlapping 50‐kb windows showed per‐chromosome median HiFi depths close to the genome‐wide median (RelToGenome ≈1×; Table S14). Both haplotype assemblies exhibited high base‐level accuracy. Genome‐wide, the estimated error rates were 1.47 × 10^−6^ for Hap1 and 1.16 × 10^−6^ for Hap2, corresponding to QVs of 58.33 and 59.37, respectively, indicating slightly higher base accuracy in Hap2. When the two assemblies were considered together, the overall error rate was 1.31 × 10^−6^ (QV 58.82) (Tables S15 and S16). Consistent with these estimates, k‐mer analysis showed that Hap1 and Hap2 individually recovered 86.38% and 86.43% of HiFi‐derived k‐mers, respectively, and together captured 97.55%, highlighting both the high completeness of each haplotype assembly and their strong complementarity.

### 2.2 Comparative Genomics and Chromosome‐Scale Genome Architecture

Comparative analysis with several published chromosome‐level *Cannabis* genomes shows that our haplotype assemblies rank among the most continuous and complete currently available, particularly in terms of gap number and contig continuity (Figure 2a; Tables S17 and S18). Both haplotype assemblies are gap‐free and have contig N50 values of approximately 80 Mb, substantially higher than those of other assemblies, including the Pink genome, which contained 61 gaps and had a contig N50 of 23.50 Mb. In addition, the GCS and EH23 haplotypes show some of the highest LAI values among the available *Cannabis* assemblies, consistent with their high assembly quality in repeat‐rich regions. By contrast, the Cs10 assembly with relatively high contig N50 values still showed lower LAI and BUSCO scores, indicating that contiguity alone does not fully capture assembly completeness (Table S17).

**FIGURE 2:**
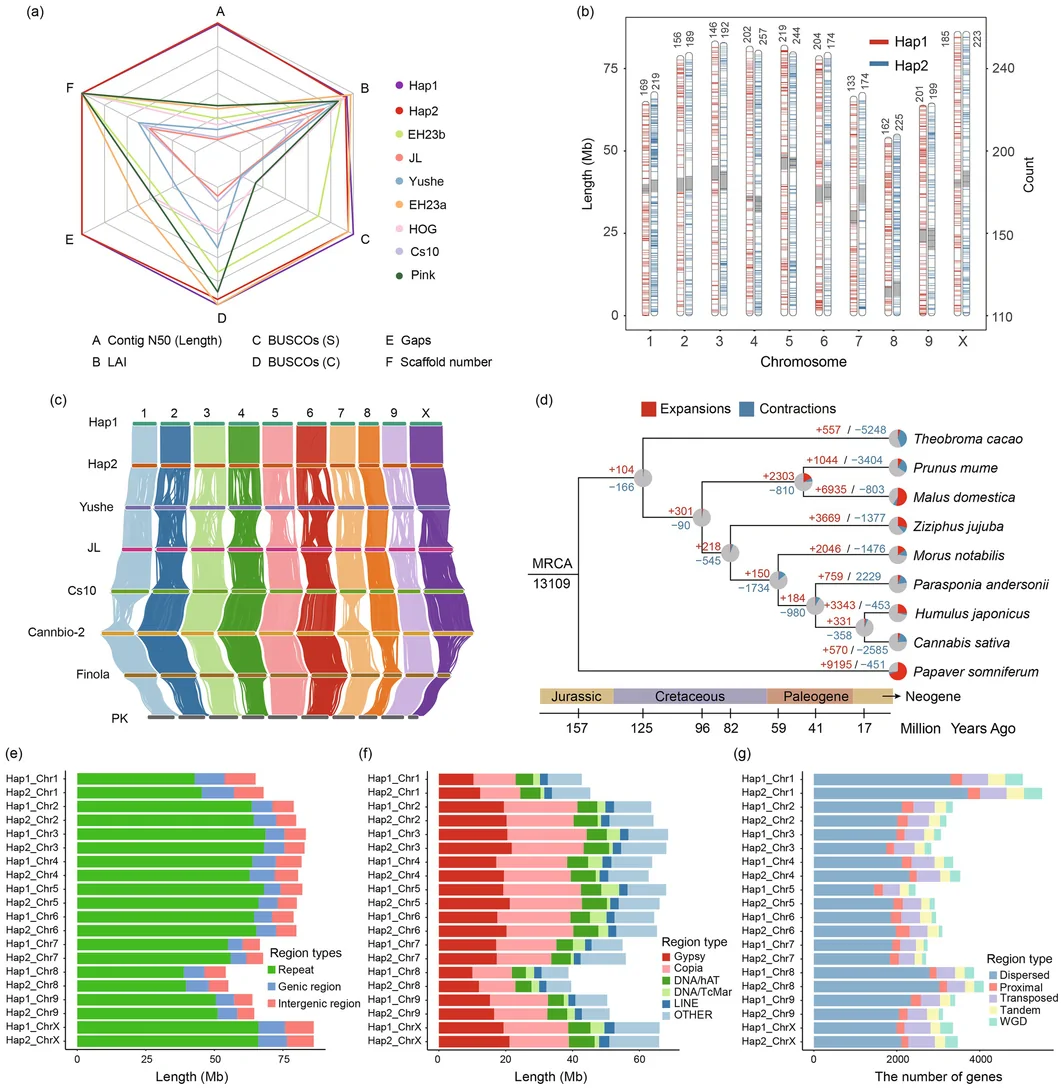
Genome architecture, collinearity, and evolutionary characteristics of *Cannabis* genomes. (a) Radar plot comparing quality metrics among selected *Cannabis* genomes, including contig N50, LAI, BUSCO single‐copy and complete scores, gap count, and scaffold number. For gap count and scaffold number, lower values are plotted closer to the outer edge, whereas for contig N50, LAI, and BUSCO scores, higher values are plotted closer to the outer edge. (b) Chromosomal distribution of haplotype‐specific genes in Hap1 and Hap2. Hap1‐specific and Hap2‐specific genes are shown in red and blue, respectively. Homologous chromosomes are aligned side by side, with grey blocks indicating putative centromeric regions. Numbers above each chromosome indicate the counts of haplotype‐specific genes in the two haplotypes. (c) Genome‐wide collinearity among representative *Cannabis* genomes. Chromosomes are ordered according to the corresponding chromosomes in the GCS assemblies, and collinear regions are connected by coloured ribbons. (d) Gene family expansion and contraction inferred across nine sampled species. Red and blue values indicate the numbers of expanded and contracted gene families, respectively, and pie charts show their relative proportions in each species. Divergence times are shown along the lower axis in millions of years (MYA), with major geological periods indicated below by coloured rectangles. The value at the root denotes the inferred gene family number in the most recent common ancestor (MRCA). (e) Chromosomal composition of the two haplotype assemblies, showing repetitive sequences, genic regions, and intergenic regions, represented in different colours. Homologous chromosomes from Hap1 and Hap2 are shown side by side for comparison. (f) Chromosomal composition of repeat elements in the two haplotype assemblies. Repeat sequences are classified into six major categories and shown in different colours. (g) Chromosomal distribution of identified duplicated genes in the two haplotype assemblies, with different colours indicating distinct duplication types.

We identified 25 619 allele gene pairs between the two haplotype assemblies of GCS and 3873 haplotype‐specific genes, including 1777 in Hap1 and 2096 in Hap2 (Figure 2b; Tables S19 and S20). These genes were unevenly distributed among chromosomes and between haplotypes. Their numbers also varied along individual chromosomes, with relatively few located in putative centromeric regions, indicating substantial divergence between the two haplotypes and reflecting the high heterozygosity of the *Cannabis* genome.

Collinear analysis across the *Cannabis* assemblies revealed strong genome‐wide synteny, with most chromosomes showing clear one‐to‐one correspondence among assemblies (Figure 2c). Despite this overall conservation, structural variations, including inversions and translocations, were detected on all chromosomes, including substantial regional divergence among assemblies. Such rearrangements were particularly frequent on Chr4, Chr5, Chr6 and ChrX.

Phylogenomic analysis based on 135 single‐copy orthologs recovered *Cannabis sativa* and *Humulus japonicus* as sister taxa, with the *Cannabis*–*Humulus* clade sister to *Parasponia andersonii* (Figure 2d; Figure S2d). Divergence times were estimated at ~17 MYA for the split between *Cannabis* and *Humulus* and ~41 MYA for the split between the *Cannabis*‐*Humulus* clade and *Parasponia*. The most recent common ancestor of the sampled species was dated to ~157 MYA and was inferred to contain 13 109 gene families. Compared with several other sampled lineages, *C. sativa* showed relatively limited gene‐family expansion and contraction, indicating comparatively stable genome evolution (Figure 2d; Table S21). Along the terminal *Cannabis* lineages, a subset of gene families nevertheless showed rapid expansion or contraction, and these were mainly enriched in secondary metabolite/glycoside biosynthesis and defence‐related functions, respectively (Figure S2e,f).

We next examined the genomic composition of the two haplotypes and the chromosomal distribution of duplicated genes (Figure 2e–g). Repetitive elements dominated both assemblies, accounting for 78.50% of each assembly, whereas gene bodies (start‐to‐stop codon regions, including introns but excluding UTRs) and intergenic regions each contributed only ~11% (Figure 2e). Among repeats, Gypsy‐ and Copia‐type LTR retrotransposons represented the major components on most chromosomes, and their overall chromosomal patterns were broadly similar between haplotypes (Figure 2f; Table S22). The composition of duplicated‐gene classes was likewise similar between the two assemblies, with dispersed duplicates forming the largest category, followed by tandem duplicates, whereas WGD‐derived duplicates were less abundant and more variable among chromosomes (Figure 2g).

### 2.3 Structural Features and Diversification of Putative Centromeric Regions

Although putative centromeric regions have been described in previous *Cannabis sativa* genome studies (Lynch et al. 2025), their structural features and diversification remain only partially resolved, particularly in a gap‐free, haplotype‐phased framework. Using the two haplotypes, we identified putative centromere positions by jointly evaluating six genomic and epigenomic features: gene density, TE (transposable element) distribution, 5mC methylation, chromatin immunoprecipitation followed by sequencing (ChIP‐seq) peaks for histone H3 lysine‐4 trimethylation (H3K4me3), GC content and sequencing depth (Figures S3 and S4). Representative examples from Hap1 Chr7 and Chr8 are shown in Figure 3a,b. In these examples, the inferred centromeric intervals correspond to regions with low gene density, reduced 5mC levels and Hi‐C contact‐mapped read depth, distinct TE distribution and elevated GC content; H3K4me3 peak levels were also uniformly low in the corresponding regions (Figures S3–S6). Because these features were used for centromere inference, they are not treated here as independent validation criteria.

**FIGURE 3:**
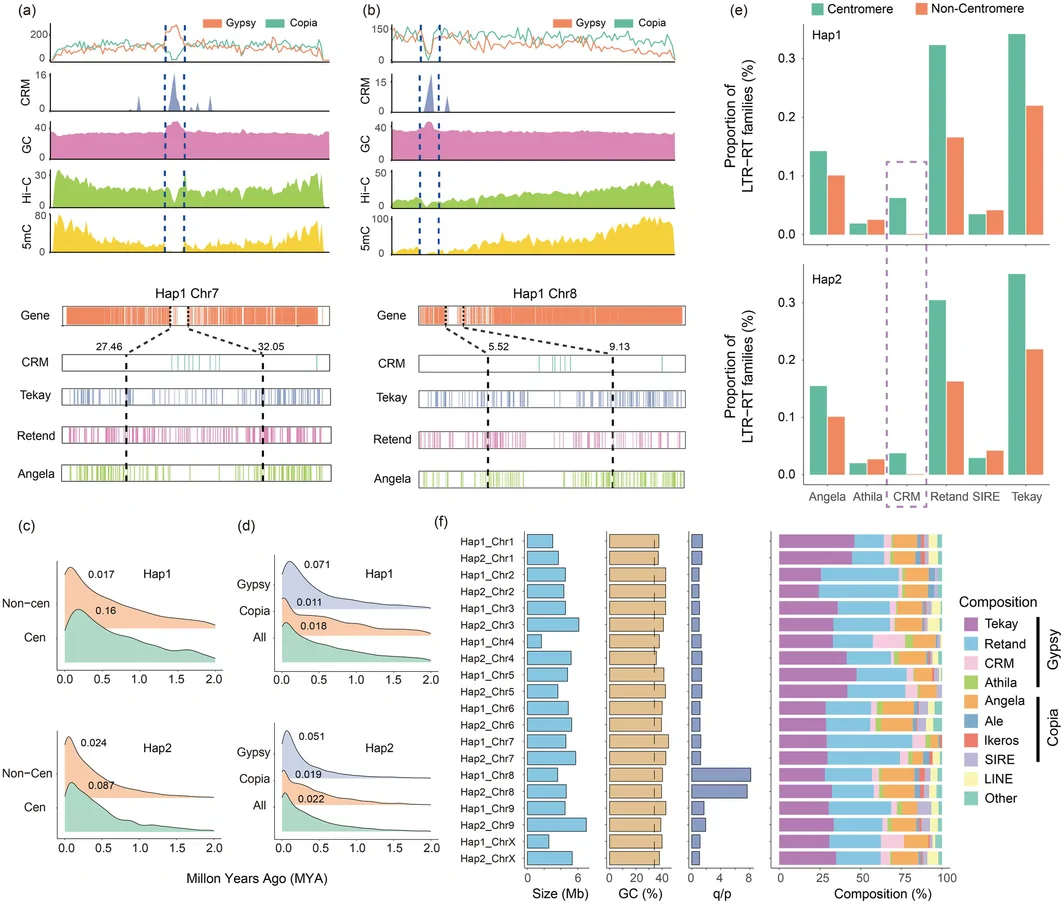
Characteristics of centromeric regions in the *Cannabis sativa* genome. (a) Representative genomic, epigenomic, and TE‐related features along Hap1 chromosome 7. Upper tracks show the densities of Gypsy and Copia LTR‐RTs, CRM elements, GC content, Hi‐C interaction intensity, and 5‐methylcytosine (5mC) levels in sliding windows. Lower tracks show the chromosome‐scale distributions of genes and CRM, Tekay, Retand and Angela LTR‐RTs along the same chromosome. Vertical dashed lines indicate the inferred centromeric boundaries. (b) Representative genomic, epigenomic and TE‐related features along Hap1 chromosome 8, shown as in panel (a). Vertical dashed lines indicate the inferred centromeric boundaries. (c) Distribution of intact LTR‐RT insertion times in centromeric (CEN) and non‐centromeric (non‐CEN) regions in Hap1 and Hap2, shown in million years ago (MYA). (d) Insertion‐time distributions of Gypsy, Copia, and all intact LTR‐RTs in Hap1 and Hap2. (e) Proportions of major LTR‐RT families in centromeric and non‐centromeric regions in Hap1 and Hap2. Values represent the proportion of each LTR‐RT family relative to the total length of centromeric or non‐centromeric regions, respectively. Major families shown include Angela, Athila, CRM, Retand, SIRE and Tekay. CRM elements are strongly enriched in centromeric regions. (f) Chromosome‐level features of the two haplotype‐phased assemblies, showing chromosome size (Mb), GC content (%), q/p arm length ratio and the composition of major repeat families.

These putative centromeric regions often overlapped Centromeric Retrotransposon of Maize (CRM)‐type TE insertions belonging to multiple CRM lineages. Gypsy elements were enriched in the centromeres of Chr2/3/5/7/9, whereas Copia elements showed lower densities across all haplotypic chromosomes. By contrast, Chr1 and Chr4 showed no consistent enrichment, and Chr6/8/X exhibited lower levels of both Gypsy and Copia elements (Figures S5 and S6). On the Hap1 copy of Chr7, the putative centromere exhibited pronounced Gypsy element enrichment, whereas the Hap1 copy of Chr8 showed markedly lower Gypsy abundance. Despite this contrast, both centromeres displayed elevated CRM content (Figure 3f). Genome‐wide analysis further confirmed that CRM elements were largely restricted to putative centromeric regions. Among LTR‐RT lineages, the Gypsy subfamilies Retand and Tekay, as well as the Copia subfamily Angela, were enriched above chromosomal averages, whereas the Copia subfamily SIRE and the Gypsy subfamily Athila were slightly decreased (Figure 3e).

We next examined the evolutionary history and quantitative properties of these centromeric regions. LTR insertions in centromeres were on average older than those in non‐centromeric regions, with Gypsy predating Copia (Figure 3c,d). Consistent with this age pattern, Gypsy elements predominated in centromeric regions (Figure 3e,f). Centromeric sizes ranged from 2 Mb (Chr4 in Hap1) to 7 Mb (Chr9 in Hap2), and they also showed locally elevated GC content, one of the features used for centromere inference. Most chromosomes showed balanced q/p arm ratios; the exception was Chr8 in both assemblies, which had a high q/p ratio (> 8), indicative of a near‐terminal centromere position (Figure 3f). Among Gypsy families, Tekay and Retand were dominant and together accounted for more than 50% of the centromeric content. Within Copia, Angela was the most abundant lineage in centromeric regions. The smallest putative centromere (Chr4 in Hap1) exhibited the highest CRM enrichment among all centromeres. When Tekay, Retand, and CRM were considered together, the centromeres on Chr5 and Chr7 in both haplotypes had the largest combined fractions. Overall, Copia remained less abundant than Gypsy in centromeric regions. Angela‐type Copia elements were the third most abundant, following Tekay and Retand. Among the less abundant elements (Athila, Ale, Ikeros, SIRE and LINE), only LINEs were consistently present across all chromosomes.

### 2.4 Shared Ancient Whole‐Genome Triplication and Genome Evolution in *Cannabis sativa*

#### 2.4.1 Evidence for a Shared Ancient Whole‐Genome Triplication

Genome‐wide intragenomic synteny analysis identified 995 and 992 syntenic blocks in Hap1 and Hap2, respectively, with similar block‐size distribution between the two phased assemblies (mean gene numbers per block: 18.77 in Hap1 and 17.56 in Hap2). This extensive and comparable intragenomic synteny is consistent with retention of a whole‐genome triplication signal in *C. sativa* (Figure S1e,f). Comparative analyses of synonymous substitutions (Ks) and fourfold‐degenerate transversions (4DTv) among *Cannabis sativa* , *Vitis vinifera* (a core eudicot sharing the ancestral *γ* event) and *Buxus austro‐yunnanensis* (an early‐diverging eudicot lacking this event) support a whole‐genome triplication (WGT) event in *C. sativa* . After lineage‐rate correction, the intragenomic Ks peaks were estimated at ~1.27 in *C. sativa* and *V. vinifera* , and at ~0.79 in *B. austro‐yunnanensis* (Figure 4a), a pattern mirrored by the 4DTv distributions (Figure 4b). Consistent with a fractionated WGT (Qiao et al. 2019), the retained blocks were discontinuous and interspersed with losses, consistent with extensive gene loss and rearrangement. In addition, most Ks values of retained block pairs exceed 1.2 (Figure S7a), consistent with the inferred WGT having occurred in the distant past. Furthermore, we analysed the synteny and Ks patterns between *H. japonicus* and *C. sativa* . High‐confidence 1:1 orthologs (defined as reciprocal best hits supported by syntenic anchors) were predominant, and syntenic depth was also dominated by a 1:1 pattern (Tables S23 and S24). Together with the uniformly low per‐chromosome BUSCO duplications (Table S7), these results support the interpretation that *C. sativa* shares the ancient WGT with core eudicots and did not show evidence of an additional lineage‐specific whole‐genome duplication (Figure 4a,b).

**FIGURE 4:**
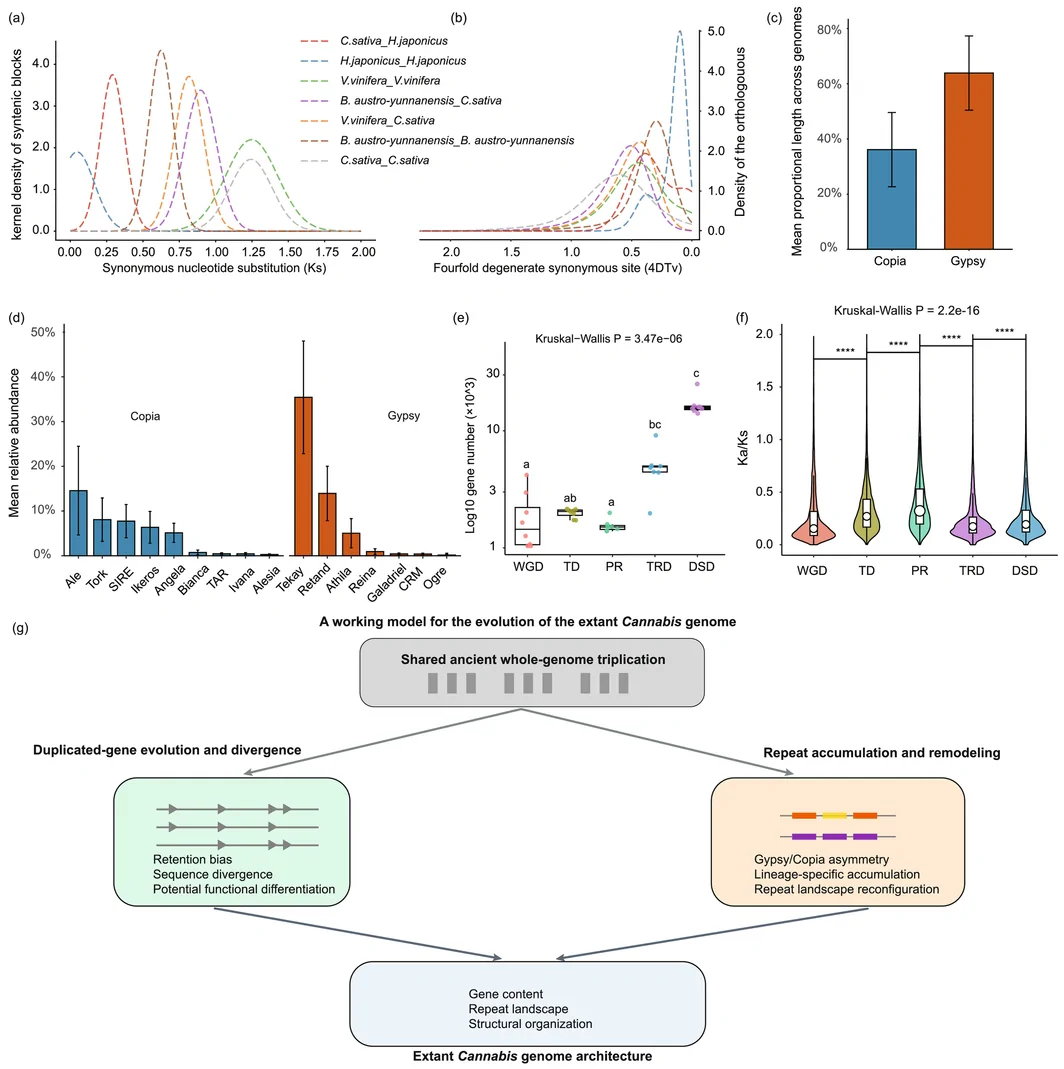
Ancient whole‐genome triplication, duplicate‐gene evolution, and repeat‐associated remodelling in the *Cannabis* genome. (a) Kernel density distributions of synonymous nucleotide substitution rates (Ks) for syntenic blocks from intra‐ and inter‐genomic comparisons among *Cannabis sativa* , *Humulus japonicus* , *Vitis vinifera* and *Buxus austro‐yunnanensis*. (b) Kernel density distributions of fourfold degenerate synonymous third‐codon transversion (4DTv) values for the same comparisons shown in (a). (c) Mean relative proportions of Copia and Gypsy LTR retrotransposons within total annotated intact LTR‐RT content across *Cannabis* assemblies. Bars represent means, and error bars represent standard deviation. (d) Mean relative abundances of major intact Copia and Gypsy lineages across *Cannabis* assemblies. Bars represent means, and error bars represent standard deviation. (e) Numbers of duplicated genes assigned to five duplication modes, including whole‐genome duplication (WGD), tandem duplication (TD), proximal duplication (PR), transposed duplication (TRD) and dispersed duplication (DSD). Values are shown on a log scale. Boxplots indicate the median (centre line), interquartile range (box), and 1.5 × interquartile range (whiskers), and points represent individual assemblies. Overall differences among duplication categories were assessed using the Kruskal‐Wallis test (*p* = 3.47 × 10^−6^), followed by Dunn's multiple‐comparison test with Benjamini–Hochberg correction. Different lowercase letters above boxes indicate significant groupings: Groups sharing at least one letter are not significantly different, whereas groups without a shared letter differ significantly. (f) Distributions of Ka/Ks ratios for duplicated gene pairs from the five duplication modes. Violin plots show the data distribution, and embedded boxplots indicate the median and interquartile range. Overall differences among groups were assessed using the Kruskal‐Wallis test (*p* = 2.2 × 10^−16^), followed by pairwise Wilcoxon rank‐sum tests. Horizontal brackets indicate the selected pairwise comparisons shown, and asterisks indicate significance levels (ns, not significant; *p < 0.05; **p < 0.01; ***p < 0.001; ****p < 0.0001). (g) A working model summarizing how a shared ancient whole‐genome triplication, together with duplicated‐gene evolution and repeat‐associated remodelling, contributed to the extant Cannabis genome architecture, including gene content, repeat landscape and structural organization.

Chromosome‐scale synteny between *C. sativa* and *V. vinifera* provided further support for the WGT in *C. sativa* (Figure S7b–g). We visualized chromosome‐scale synteny by plotting anchor gene pairs colour‐coded by Ks and observed an approximate 3:1 syntenic depth of the *C. sativa* genome relative to individual *V. vinifera* chromosomes, with large continuous collinear blocks separated by losses. Zoom‐in views showed three *C. sativa* segments that retain gene‐order collinearity with the same *V. vinifera* region, and anchor pairs exhibit consistent Ks signals across these blocks (Figure S7b–g). Together, these patterns of extensive segmental duplication followed by gene loss further support the inference of the shared WGT signal in *Cannabis*. Consistent patterns were also recovered in comparison with *B. austro‐yunnanensis* and its reconstructed ancestral karyotypes, providing independent support for the shared WGT signature retained in *C. sativa* (Figures S8 and S9).

#### 2.4.2 Genome‐Wide Distribution and Dynamics of LTR Retrotransposons

Genome‐wide analysis of intact LTR retrotransposons (LTR‐RTs) across *Cannabis* assemblies revealed a clear asymmetry in LTR composition. Within the total intact LTR‐RT fraction, Gypsy elements accounted for a larger proportion than Copia elements, representing approximately 60% and 40%, respectively (Figure 4c). At the lineage level, Tekay was the dominant Gypsy lineage, whereas Ale was the most abundant Copia lineage, with the remaining lineages contributing comparatively smaller fractions (Figure 4d). The divergence patterns of Copia and Gypsy across multiple assemblies were broadly similar, and no significant difference was detected between them (Figure S10a).

Raw LTR‐RT distributions in *Cannabis* assemblies, such as Hap1 showed a non‐uniform chromosomal pattern (Figure S11a). Principal component analysis (PCA) and phylogenetic reconstructions of intact LTR‐RTs revealed distinct composition patterns between Copia and Gypsy elements (Figure S11b,c). Copia elements were relatively evenly distributed across families, whereas Gypsy elements were dominated by the Tekay and Retand lineages, with few representatives from other families. The same pattern was observed in multiple assemblies (Table S25; Figures S12 and S13), except in the Cs10 assembly, in which Copia and Gypsy showed similar proportions, and the PCA did not show clear separation along PC1–PC3.

Divergence‐from‐consensus analysis of LTR‐RT showed that the divergence rate ranged from 10% to 20% (Figure S11d), with different patterns among the various types. Gypsy showed the highest divergence rates, followed by Unknown, whereas Copia was the lowest.

The intact LTR‐RT landscape showed similar patterns among various assemblies in terms of total length and counts of subfamilies (Figure S14a,b, Table S25). The ratio of intact/raw LTR‐RTs ranged from 14% to 25% (Table S26). In terms of total intact LTR‐RT content, Gypsy was dominant, followed by Unknown elements, whereas Copia contributed the least. The count of intact LTR‐RTs was also consistent with this pattern, except in the Cs10 and PK assemblies. The distribution of LAI among the chromosomes in different assemblies was uneven and generally declined in putative centromeric and telomeric regions (Figure S14c; Tables S17 and S25). Within intact LTR‐RT families, Tekay and Retand showed the highest relative abundance among Gypsy, whereas Ale was the most abundant lineage in Copia (Figure 4d). In terms of total length and count, Tekay and Retand were dominant, whereas mean length was greatest in the Ogre lineage, followed by Retand (Figure S14d–f). The number of raw LTR‐RTs showed a highly consistent pattern among most assemblies, with Unknown > Copia > Gypsy, except in the PK assemblies (Figure S15).

Together, these analyses show substantial differences in abundance and lineage composition among LTR‐RT categories and assemblies, as well as variation in divergence patterns.

#### 2.4.3 Genome‐Wide Patterns and Evolutionary Divergence of Duplicated Genes

We characterized duplicated genes in the *Cannabis* genomes according to five duplication modes. Significant differences were observed among categories (Figure 4e; Figure S16a; Table S27), with DSD genes representing the largest fraction, followed by TRD, whereas WGD‐derived genes were consistently the least abundant or among the least abundant classes across assemblies.

The number of homologous genes on each chromosome also differed substantially between the two haplotypic assemblies of GCS, whereas corresponding haplotype chromosomes retained broadly similar overall patterns. This pattern mirrors the chromosomal distribution of duplicated genes (Figure 2g) and together highlights the contribution of retained duplicated homologues to chromosomal gene content.

The evolutionary constraints acting on these duplicate classes also differed significantly (Figure 4f). In general, TD and PR pairs exhibited higher Ka/Ks values than WGD‐derived pairs, suggesting faster sequence divergence or relatively relaxed selective constraint in locally duplicated genes, whereas WGD‐derived duplicates were more conserved. Sequence divergence among duplicate‐gene classes, summarized using median Ka/Ks values, showed a similar pattern. Consistent with the overall distribution, PR and TD duplicates showed relatively elevated median Ka/Ks values, whereas WGD‐derived duplicates remained comparatively conserved (Figure 4f; Figure S16b). Pairwise comparisons of median differences and Cliff's delta further showed that the strongest contrasts involved PR relative to WGD, TRD and DSD, whereas the difference between TRD and DSD was small (Figure S16c,d). These results reinforce that locally duplicated genes tend to exhibit greater sequence divergence than WGD‐derived duplicates.

The Ka, Ks, and Ka/Ks values across the various assemblies also showed distinct patterns among duplication modes. WGD, DSD, and TRD pairs generally showed high Ks values (2.0–5.0), whereas their Ka/Ks values remained very low (< 0.3), indicating that these duplicated genes are evolutionarily ancient and have been subject to strong functional constraint and pervasive purifying selection. In Yushe and Cs10, WGD pairs displayed slightly lower Ks values than in the other assemblies, likely reflecting lower divergence among retained gamma‐derived paralogs rather than a lineage‐specific recent WGD event. Tandem and proximal duplication gene pairs exhibited low Ks values, consistent with recent expansion. The duplicated genes also showed different retention biases (Figure S16c), and the diversity of Ka and Ks values indicates potential functional diversification among duplication classes (Figure S16d,e).

Together, the duplicated‐gene analysis suggests that, after the shared WGT, DSD and TRD contributed substantially to the older duplicated‐gene fraction, whereas TD and PR were more associated with recent expansion. Together with LTR‐RT landscape remodelling, these patterns indicate an important role of duplicated‐gene evolution in the present genomic landscape of *Cannabis* (Figure 4g).

#### 2.4.4 Ancestral Origin and Lineage‐Specific Expansion of Cannabinoid Synthase Genes

An evolutionary framework for cannabinoid synthase genes was reconstructed by integrating orthogroup (OG) distributions across the complete cannabinoid biosynthesis pathway with phylogenetic analysis of proteins grouped by domain composition into FAD (proteins containing only the FAD (flavin adenine dinucleotide)‐binding_4 domain), BBE (BBE (berberine bridge enzyme)‐like proteins lacking a recognizable FAD‐binding_4 domain), BBE + FAD (canonical BBE‐like proteins containing both BBE‐like and FAD‐binding_4 domains) across representative eudicot assemblies (Figure 5a,b). Most OA‐ and GPP‐pathway OGs were broadly conserved among the sampled species, whereas the OG containing the characterized synthases *CBDAS*, *THCAS*, and *CBCAS* was detected only in *Cannabis sativa* and was absent from *Humulus* and the remaining sampled taxa. A similar restricted pattern was also observed for the OGs harbouring *OLS* and *aPT4*, whereas the other OA‐ and GPP‐pathway genes remained broadly conserved (Figure 5a). Thus, unlike the more conserved upstream pathway genes, the cannabinoid‐specific branch of the pathway showed a highly restricted distribution and a distinct copy‐number pattern in *Cannabis*. Homology searches requiring the conserved catalytic residues His292 and Tyr417 (Shoyama et al. 2005) likewise detected canonical cannabinoid synthase homologues only in *Cannabis* in our sampling (Table S28; Figure S17a,b). These findings are consistent with the hypothesis that cannabinoid‐synthase genes originated from duplication and subsequent functional innovation within the BBE‐like lineage, driven by functional innovation (Van Velzen and Schranz 2021).

**FIGURE 5:**
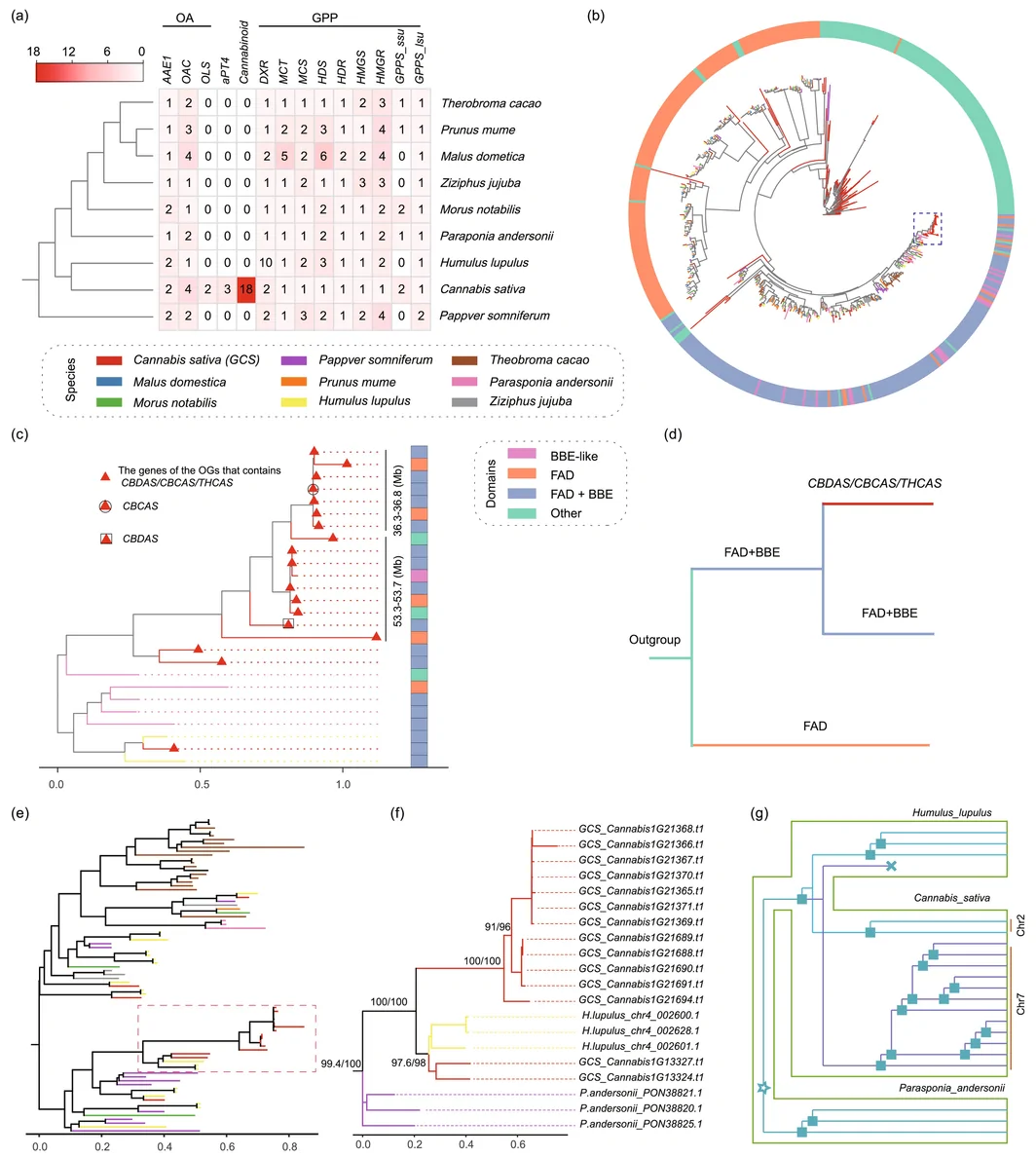
Evolutionary conservation and lineage‐specific expansion of cannabinoid synthase genes in *Cannabis sativa* . (a) Copy number matrix of OGs across nine eudicot species. Genes from the OA branch and the upstream GPP pathway, together with the cannabinoid synthase genes *CBDAS*, *CBCAS* and *THCAS*, are labelled above the heatmap. The species phylogeny is shown on the left. Numbers within boxes indicate gene counts for each species in the corresponding OG, and colour intensity ranges from white (low) to red (high). (b) Phylogenetic tree of BBE‐like proteins across nine eudicot species, rooted with non‐BBE FAD oxidoreductase outgroups. Branch colours denote species, consistent with panels (c), (e), and (f), and the outer ring indicates domain architectures. Red triangles mark genes belonging to the OG containing *CBDAS*, *THCAS* and *CBCAS*. The subtree containing this OG is highlighted by a dashed box and shown in detail in panel (c). (c) Magnified view of the cannabinoid synthase‐containing clade. *CBDAS* and *CBCAS* are specifically indicated, domain architectures are shown to the right of the tree, and the corresponding genomic intervals on chromosome 7 are indicated alongside the tree. (d) Conceptual model for the evolution of cannabinoid synthases within the FAD‐dependent BBE‐like family. Outgroup sequences (other FAD‐dependent, non‐BBE oxidoreductases) were used to root the tree. (e) Maximum‐likelihood phylogeny of FAD‐dependent BBE‐like genes with high structural similarity to *CBDAS*, *CBCAS*, and *THCAS*. (f) Enlarged view of the dashed‐box clade in panel (e) used for hypothesis testing. The AU‐supported topology is shown with homologues of *Parasponia andersonii* as the outgroup, and internal node labels indicate SH‐aLRT and UFBoot support values. (g) Reconciliation of the AU‐supported topology with the rooted species tree. The star, square, and cross represent family origin, duplication and loss, respectively.

Comparing domain architectures across multiple species, phylogenetic analysis resolved three well‐supported clades that correspond to domain composition. In this tree, the cannabinoid‐synthase OG genes formed a discrete subclade within the BBE‐like (FAD‐dependent) lineage rather than among other FAD‐dependent non‐BBE oxidoreductases (Figure 5b). We further examined the subtree containing these cannabinoid‐synthase OG genes and found that it comprised 26 sequences in total, including 24 from *C. sativa* and two from *H. japonicus* (Figure 5c). Within this subtree, 17 of 18 OGs containing cannabinoid‐synthase genes clustered tightly together, whereas the remaining OG grouped with the *Humulus* sequences. This pattern is consistent with lineage‐specific expansion of the cannabinoid‐synthase OG in *Cannabis*. In our assemblies, only a minority of paralogs encoded intact oxidocyclases that catalyse the terminal step of cannabinoid biosynthesis, whereas the majority carried frameshifts or premature stop codons, consistent with extensive pseudogenization within the expanded synthase regions (Lynch et al. 2025).

*CBDAS* and *CBCAS* resided at separate loci on Chr7 (53.3–53.7 Mb and 36.25–36.75 Mb, respectively), consistent with their placement in two distinct phylogenetic subclades. Based on domain architecture and phylogenetic evidence, FAD‐binding proteins lacking a BBE domain provide the outgroup context, whereas the family core consisted of the BBE‐like lineage bearing both FAD and BBE domains. In *C. sativa* , this BBE‐like lineage underwent duplication and divergence, giving rise to the cannabinoid synthases *CBDAS*, *THCAS* and *CBCAS* which catalyse the terminal steps of cannabinoid biosynthesis (Figure 5c). *GCS_Cannabis1G21694.t1* was annotated as a putative *CBDAS* homologue based on eggNOG, InterProScan, and NT/NR annotations. Consistent with this annotation, phylogenetic analysis of BBE‐like sequences across multiple species placed it within the *CBDAS*‐associated lineage, where it occupied a relatively basal position among the *CBDAS*‐related copies. *Cannabis* copies clustered together, whereas homologues from related taxa fell outside this cluster (Figure S17). Phylogenetic relationships further indicated that *CBCAS* and *THCAS* are closely related, whereas the extant *CBDAS* lineage occupied a relatively basal position within this clade (Figure 5d). Together with the conserved FAD + BBE domain architecture, these patterns are consistent with duplication and subsequent divergence.

To clarify the origin of cannabinoid oxidocyclase genes, we retrieved a set of *CBDAS*/*CBCAS*/*THCAS* homologues and retained only sequences in which both the FAD‐binding_4 and BBE‐like domains were present, with each domain covering ≥ 70% of the amino acid sequence. A maximum‐likelihood phylogeny based on this curated alignment recovered a well‐supported clade containing *Cannabis sativa* and *Humulus japonicus* copies (Figure 5e). We then evaluated three mutually exclusive evolutionary hypotheses for this clade using *Parasponia andersonii* as the outgroup: T1, H,(C2,(C7…)); T2, H,(C7,(C2…)); and T3, (H,C2),C7…. Approximately Unbiased (AU) topology testing strongly favoured T3 (p‐AU = 0.967) and rejected T1 and T2 (p‐AU ≈ 0.03; Figure 5f); key relationships were strongly supported by SH‐aLRT/UFBoot. Reconciling the AU‐supported topology with the rooted species tree placed the first duplication in the common *Cannabis*–*Humulus* ancestor, followed by loss on the *Humulus* lineage and subsequent duplications within *Cannabis* (Figure 5g). These lineage‐specific duplications are consistent with the Chr7 tandem cluster in *Cannabis*, whereas the Chr2 copy may retain a relatively ancestral phylogenetic position within this gene family. The topology test and reconciliation jointly support the idea that an ancestral duplication, followed by differential retention and expansion, is more likely than a single recent intragenomic transposition between *Cannabis* chromosomes.

### 2.5 Structural Conservation and Functional Differentiation of Cannabinoid Synthase Genes

In the cannabinoid biosynthetic pathway leading to both CBDA and THCA, GPP and OA serve as shared precursors. GPP is a common precursor for terpenoid synthesis, whereas OA is derived from fatty acids via the hexanoate pathway. OA is prenylated by GPP through *aPT4* to produce CBGA, which is subsequently oxidocyclized into THCA, CBDA, and CBCA by *THCAS, CBDAS* and *CBCAS*, respectively (Figure 6a). Although *THCAS* and *CBDAS* are absent from some *Cannabis* accessions, PAML analysis of the coding sequences identified in our assemblies revealed no evidence of positive selection (Table S29). Among accessions that retain them, these genes show a signature of strong purifying selection, consistent with the selective maintenance of their role in cannabinoid biosynthesis.

**FIGURE 6:**
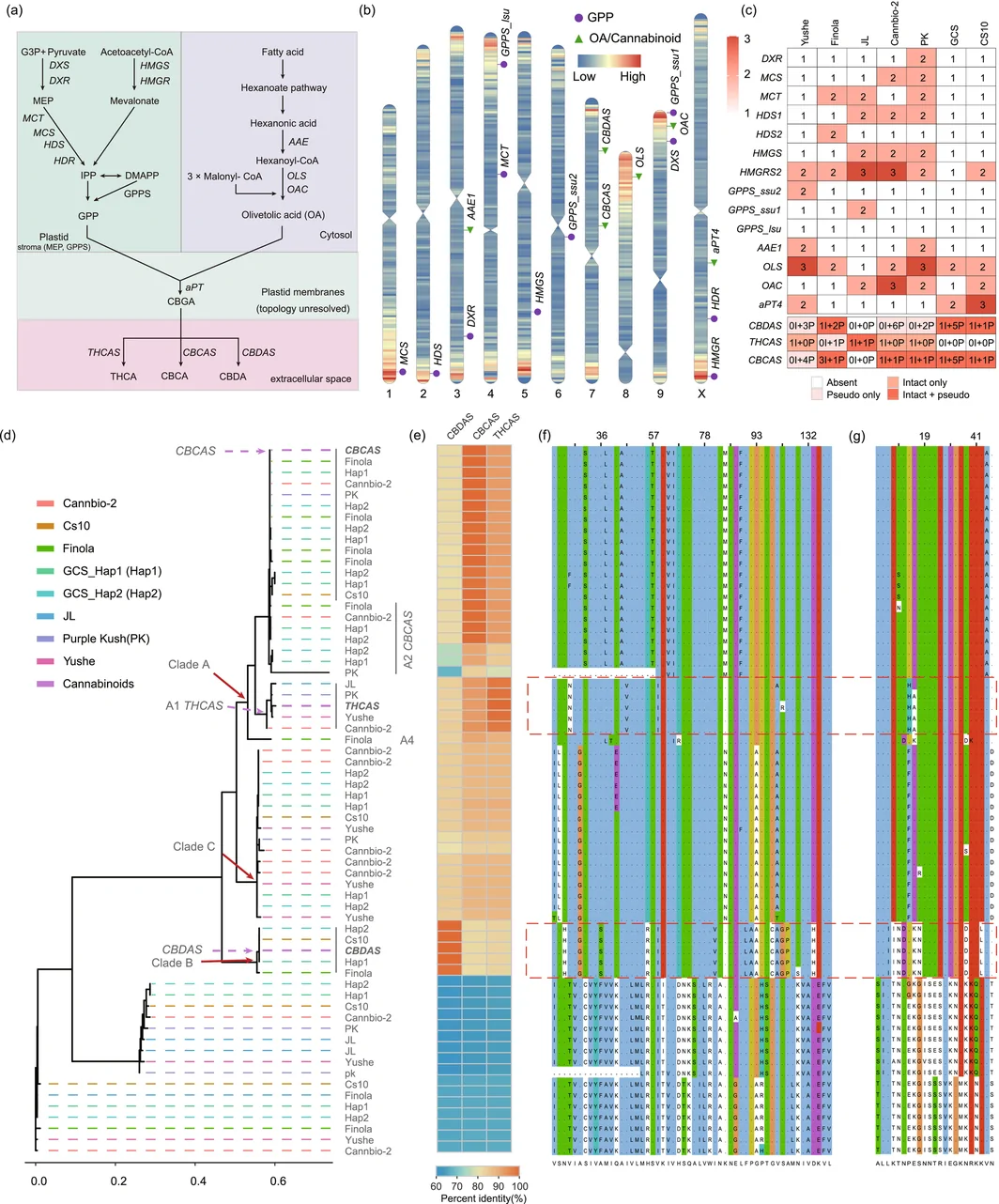
Structural variation and sequence divergence of cannabinoid synthase genes in *Cannabis sativa* . (a) Overview of the cannabinoid biosynthetic pathway, highlighting contributions from the plastid‐localized MEP pathway and the cytosolic hexanoate pathway. Different subcellular compartments are indicated by distinct colours. (b) Chromosomal distribution of cannabinoid‐biosynthetic pathway genes in GCS_Hap1 overlaid on the backbone‐relative sequence variation landscape across eight *Cannabis sativa* accessions. Background colours indicate regional variation density from low (blue) to high (red), providing genomic context for pathway loci at the sequence level. Genes of the GPP branch are marked by purple circles, whereas genes of the OA/cannabinoid branch are marked by green triangles. Collapsed intervals indicate centromeric regions. (c) Copy number variation of cannabinoid‐biosynthetic genes across *C. sativa* accessions. The upper panel shows copy numbers of orthogroups corresponding to core pathway genes. The lower panel summarizes the status of the three cannabinoid synthase groups (*CBDAS*, *THCAS* and *CBCAS*), with each cell indicating the numbers of intact (I) and non‐intact/pseudogene‐like (P) copies detected in the corresponding OG in each accession. Colour intensity in the upper panel ranges from white to red, indicating low to high copy number. (d) Maximum‐likelihood phylogeny of proteins containing both FAD and BBE domains from eight *C. sativa* accessions. Accession identities are indicated by coloured lines adjacent to the tips. The cannabinoid synthase genes *CBDAS*, *THCAS* and *CBCAS* form distinct clades, each indicated by dashed arrows. Accession names are shown on the right. Branches corresponding to publicly available reference sequences of *THCAS*, *CBCAS* and *CBDAS* are shown in bold. (e) Heatmap showing pairwise percent identity among *CBDAS*, *THCAS, CBCAS*, and their homologous protein sequences used in the phylogenetic analysis. Identity values range from 60 to 100, with a colour‐coded scale from low to high. Reference enzyme protein IDs are shown at the top of the heatmap. (f, g) Visualization of non‐conserved regions within the FAD (f) and BBE (g) domains. Amino acid residues are colour‐coded based on their conservation and physicochemical properties. Red dashed boxes highlight sequence regions of *THCAS* and *CBDAS*. White cells with ‘–’ indicate missing residues. The top axis shows amino acid positions, and the bottom row shows the consensus residue at each position.

To place cannabinoid pathway genes in a broader structural‐variation context, we mapped them onto the chromosome‐scale PAV landscape constructed from the eight *Cannabis* assemblies (Figure 6b). Pathway genes were distributed across regions with contrasting PAV densities, with some located in PAV‐enriched intervals and others in relatively conserved chromosomal regions, indicating that different branches of the pathway are embedded in distinct variation backgrounds. Consistent with this pattern, copy‐number variation among most non‐terminal synthase pathway genes was generally limited, although modest expansion was detected in several assemblies (Figure 6c; Table S30). In contrast, the three key cannabinoid synthase genes, *CBDAS*, *THCAS* and *CBCAS*, showed much stronger between‐genome variation in copy state, including intact copies, pseudogene and complete absence within the corresponding OG categories (Figure 6c). Notably, no more than one intact *CBDAS* or *THCAS* copy was detected in any haploid assembly, consistent with the previous reports (Lynch et al. 2025).

To confirm the evolutionary trajectory of cannabinoid synthase genes, we reconstructed a phylogeny of FAD‐ and BBE‐domain proteins across representative *C. sativa* assemblies. The tree shows that *CBDAS*, *THCAS* and *CBCAS* form a monophyletic clade within the FAD + BBE lineage and are separate from Chr2 homologues. *THCAS* and *CBDAS* in this phylogeny were located in the subclade A1 and B (Figure 6d). These subclades corresponded to the intact cannabinoid synthase genes and were consistent with the distribution shown in Figure 6c. Protein sequence similarity analysis supports this evolutionary trajectory: cannabinoid synthases and their homologous copies exhibit high intra‐clade identity. Notably, the basal‐positioned *CBDAS*‐like copy clusters more closely with the *CBCAS*‐ and *THCAS*‐associated sequences than with the extant *CBDAS* lineage, consistent with differential divergence of paralogous copies after duplication (Figure 6e). Multiple sequence alignments of the FAD and BBE domains identified branch‐specific residues unique to *CBDAS* and *THCAS*, including H, S, V and C residues in the FAD domain of *CBDAS*, and N, V and I residues in *THCAS*, which are highlighted by a red dashed box (Figure 6f,g), consistent with domain‐mediated functional differentiation.

Together, these results support a model in which cannabinoid synthase genes in *Cannabis* derive from a BBE‐like ancestral duplication on the *Cannabis*–*Humulus* stem lineage. In this scenario, the genes on Chr2 likely represent retained ancestral duplicate copies, whereas a derived lineage underwent tandem duplication and divergence on Chr7, giving rise to the *CBDAS*, *THCAS* and *CBCAS* clade at two separate loci. This evolutionary framework further clarifies the origin and functional specialization of the key enzymes that catalyse the terminal steps of cannabinoid biosynthesis.

### 2.6 Genomic Organization and Expression of Cannabinoid Pathway Genes

Most cannabinoid biosynthesis–related genes are largely retained as intact copies across *C. sativa* assemblies, although their chromosomal positions vary (Figure 7a). *AAE1* maps to Chr3 in all accessions except for Finola, where it lies on Chr4. *OLS*, *OAC* and *aPT4* reside on Chr8, Chr9 and ChrX, respectively; *aPT4* is absent in Finola due to the incomplete assembly of ChrX. *THCAS* and *CBDAS* are located on Chr7 in most assemblies but on Chr4 in JL. In PK, the *THCAS* genes remain on unanchored contigs (AGQN03010731.1, AGQN03010271.1), and in Finola, they are located on scaffolds QKVJ02001794.1 and QKVJ02004887.1. Cannabinoid synthase‐associated regions were defined according to syntenic block context. Blocks containing *THCAS* or *CBDAS* were designated as the *THCAS*‐region and *CBDAS*‐region, respectively, and the corresponding syntenic blocks in other genomes were classified into the same regional categories even when no cannabinoid synthase gene was retained within the block. Synteny analysis shows that cannabinoid synthase loci occur in distinct *CBDAS*‐associated and *THCAS*‐associated blocks in Hap1, Hap2, and Yushe, whereas they fall within the same block in other assemblies (Figure 7a). This pattern is consistent with lineage–specific rearrangements in the Hap1, Hap2 and Yushe lineages.

**FIGURE 7:**
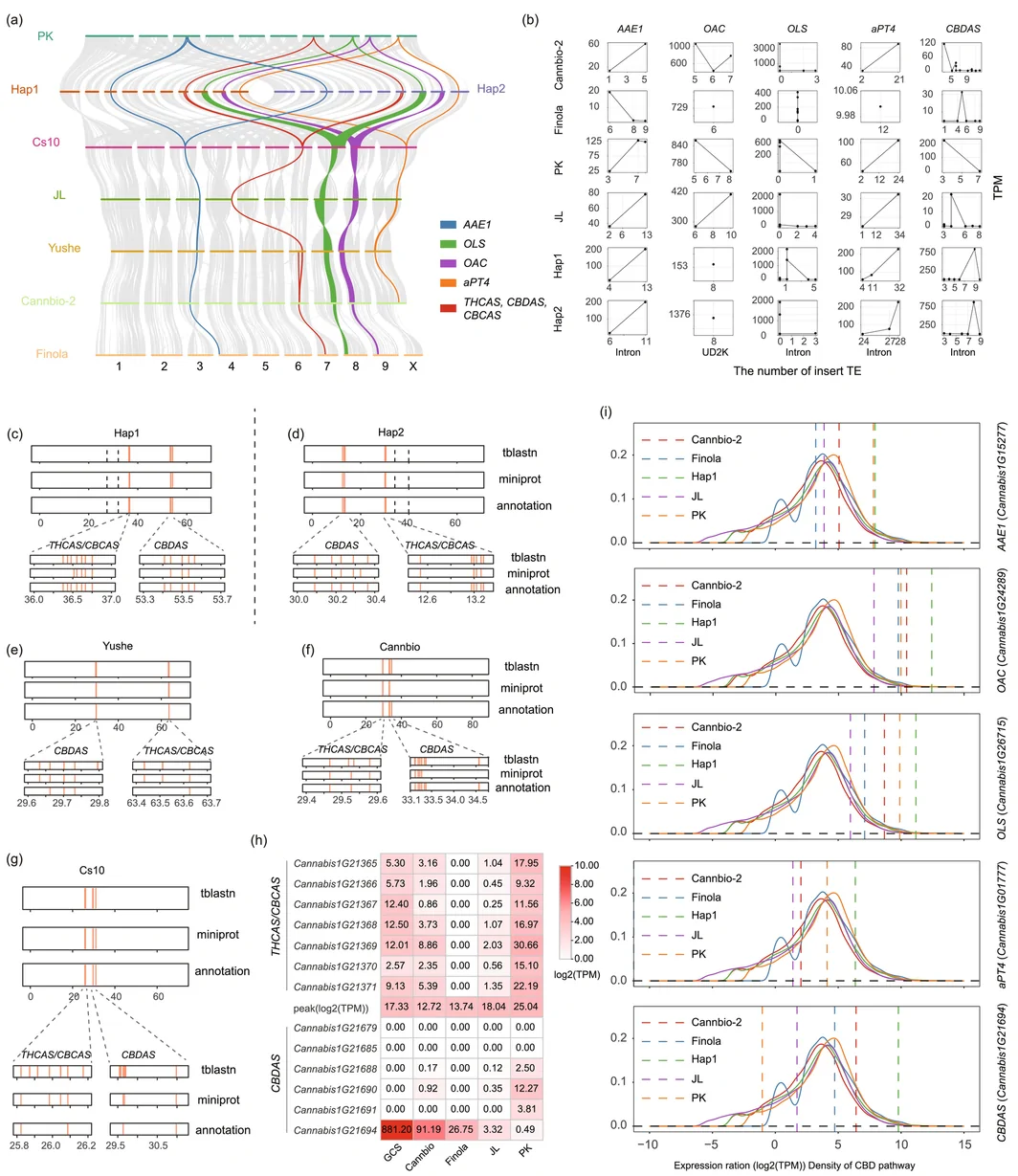
Cannabinoid biosynthetic pathway, genomic distribution, and comparative genomics in *Cannabis sativa* . (a) Chromosome‐level synteny analysis across eight *C. sativa* assemblies. Syntenic blocks containing key cannabinoid biosynthetic genes are highlighted, including *AAE1* (blue), *OLS* (green), *OAC* (purple), *aPT4* (orange) and *THCAS/CBDAS/CBCAS* (red). Different assemblies are represented by separate chromosome sets, with accession names on the left (Hap2 on the right) and corresponding chromosome IDs from the GCS reference along the bottom. GCS_Hap1 and GCS_Hap2 are shown separately to preserve haplotype‐phased syntenic context around the focal synthase‐associated block, which would be obscured if the two highly collinear haplotypes were collapsed into a single track. (b) Relationship between the expression levels of selected cannabinoid pathway genes and nearby TE insertions across accessions. TE insertions located in exons, introns and within 2 kb upstream or downstream (UD2K) regions were analysed. Expression levels (TPM) of homologous genes were compared among accessions. TE insertion counts and categories associated with each homologous gene are shown. Genes lacking homologues in a given accession are represented as dots. Accession names are shown on the left, with TPM values indicated to the left of each expression panel. (c–g) Chromosomal regions homologous to GCS chromosome 7 across five representative *C. sativa* assemblies: Hap1, Hap2, Yushe, Cannbio‐2 and Cs10. Coloured vertical lines indicate different cannabinoid synthase‐related classes: Orange, *CBDAS*‐region‐like genes; pink, *CBDAS*; teal green, *THCAS*/*CBCAS*‐region‐like genes; purple, *CBCAS*; and blue, *THCAS*. In panels (c) and (d), dashed lines indicate approximate centromeric regions. Each panel includes three evidence tracks: TBLASTN hits, miniprot‐inferred loci and gene models from assembly‐specific annotations. Inserts provide enlarged views of the *THCAS*/*CBCAS* and *CBDAS* regions, highlighting their local genomic organization. (h) Expression matrix of homologous genes located in the *THCAS/CBCAS‐* and *CBDAS*‐associated regions across five *C. sativa* accessions. Gene coordinates are based on the Hap1 reference genome. The row labelled ‘peak(log_2_(TPM))’ gives, for each accession, the peak of the genome‐wide log_2_(TPM) distribution, used as a reference to compare expression across transcriptomes. The heatmap shows relative expression levels, ranging from white (low) to red (high). Rows indicate gene names and their associated genomic region. (i) Density plots of log_2_(TPM) values for all genes in the cannabinoid biosynthetic pathway across five *Cannabis* accessions. Vertical dashed lines indicate the expression levels of five key biosynthetic genes (*AAE1*, *OAC*, *OLS*, *aPT4* and *CBDAS*) in each accession. Colours represent different accessions. Gene names and their corresponding Hap1 gene IDs are shown on the right.

We further examined the potential impact of TE insertions on gene expression in the gene families to which the cannabinoid biosynthetic pathway genes belong (Table S31). For several gene families encoding enzymes involved in the upstream pathway steps, such as *AAE1* and *aPT4*, expression levels were positively correlated with intronic TE insertions, whereas genes encoding *OLS* showed a negative correlation. This pattern suggests that TE insertions are associated with gene‐specific regulatory responses. In contrast, cannabinoid synthase genes displayed a distinctive pattern: their expression peaked at a specific number of TE insertions and declined when the counts were either higher or lower (Figure 7b). These results reveal a nonlinear relationship between TE insertion number and cannabinoid pathway gene expression consistent with a dosage‐sensitive regulatory effect across the examined accessions.

In high‐CBDA accessions Cannbio‐2 and GCS, expression profiling during flowering confirmed *CBDAS* expression. Additional expression data from Finola, PK, and JL were also included for comparison. Although *THCAS* and *CBDAS* share 82% protein sequence similarity, their expression levels vary widely (Figure 6e; Figure S18). Among upstream genes, *AAE1* exhibited the highest expression in GCS and PK, whereas *OAC* expression decreased progressively from GCS to Cannbio‐2, PK and Finola. *OLS* peaked in GCS and was next highest in PK; *aPT4* likewise displayed its highest expression levels in GCS and PK. *CBDAS* reached maximal expression in GCS but was undetectable in PK due to the absence of the gene (Figure 7i).

Across the higher‐quality assemblies, cannabinoid oxidocyclase genes assigned to subclades A1 (*THCAS*), A2 (*CBCAS*) and B1 (*CDBAS*) showed distinct genomic distributions. In the selected assemblies, these genes are located on Chr7 within divergent tandem arrays (Figure 7c–g). The genomic distances between the *THCAS* and *CBDAS* arrays ranged from 10 to 30 Mb in Hap1, Hap2 and Yushe, but were reduced to 4–5 Mb in Cannbio‐2 and Cs10. Despite the lower overall quality of the Cs10 assembly, its linkage‐map–anchored scaffolds likewise revealed a shortened *THCAS*–*CBDAS* interval. The inferred gene positions agree with the annotations in Hap1 and Hap2, but diverge in Yushe, Cannbio‐2 and Cs10, likely due to incorrectly scaffolded regions.

Gene expression profiling of the tandem repeat region on Chr7, using Hap1 as a reference, showed that none of the *THCAS/CBCAS* loci reached the global peak of log_2_(Transcripts Per Million (TPM)) expression observed in blooming flowers (Figure 7h). Conversely, the *CBDAS* gene (*Cannabis1G21694*) showed strong expression in GCS, Cannbio‐2 and Finola. Although a weak transcriptional signal was observed over the orthologous region in JL and PK, these accessions do not contain an intact *CBDAS* gene; therefore, this signal was not interpreted as intact *CBDAS* expression. The remaining tandem arrayed genes were either weakly expressed or transcriptionally silent. Notably, the expression of *Cannabis1G21371* (*CBCAS*) did not approach the global peak and remained clearly below that of *CBDAS*, indicating that *CBCAS* is expressed at relatively low levels during blooming.

## 3 Discussion

Over the past two decades, *Cannabis* genomics has progressed from fragmented short‐read assemblies to chromosome‐scale references based on long‐read sequencing. These advances have substantially improved genome continuity and overall structural resolution, but accurate sequence placement and orientation in highly heterozygous, repeat‐rich regions have remained challenging. Although several high‐quality *C. sativa* assemblies are now available (Lynch et al. 2025; Wei et al. 2023), most still contain residual gaps (Table S17), and some chromosomes were ordered or oriented using reference‐guided scaffolding. The gap‐free, haplotype‐phased assemblies generated here provide a more complete framework for re‐evaluating the polyploidy history of *Cannabis*, reconstructing the origin and chromosomal partitioning of cannabinoid synthases, and characterizing the structural and regulatory conservation of the cannabinoid biosynthetic pathway.

Our analyses place the polyploidy history of *C. sativa* within the broader framework of core‐eudicot evolution. Earlier studies, based mainly on Ks or 4DTv peak patterns derived from gap‐containing chromosome‐scale assemblies, interpreted wild and seed‐type *Cannabis* as having undergone three rounds of whole‐genome duplication (Gao et al. 2020; Wei et al. 2023). By contrast, a recent *Cannabis* pangenome study, after excluding TE‐associated genes, supported a single whole‐genome triplication event and interpreted the more recent signal mainly in relation to transposable element activity and tandem duplication (Lynch et al. 2025). Our gap‐free assemblies, together with synteny‐based paralog detection and cross‐species comparisons, likewise recovered only a single WGT signal consistent with the shared core‐eudicot *γ* event, while providing no evidence for an additional younger WGD in *C. sativa* . This interpretation is also consistent with other lines of evidence supporting a diploid‐like genomic state rather than a recent genome‐wide duplication (Tables S7, S23 and S24). At the same time, our analyses highlight one important point of difference: whereas the pangenome inference was based on chromosome‐scale and contig‐level assemblies, our comparisons based on uniformly gap‐free *Cannabis* genomes recovered only the ancient triplication‐associated signal and did not reveal a distinct recent peak indicative of an additional lineage‐specific large‐scale duplication event. We therefore suggest that at least part of the discrepancy among studies may reflect differences in assembly continuity and analytical framework. In a genome such as *Cannabis*, with high heterozygosity and abundant repeats, gap‐containing assemblies may complicate the placement and orientation of highly similar sequences and thereby influence the apparent distribution of recent duplicate signals.

The younger peak observed among WGD‐classified duplicate genes (Figure S16e) is therefore better interpreted as a secondary gene‐pair‐level signal that arose after the shared ancient WGT, rather than as evidence for an additional whole‐genome duplication event. This distinction likely reflects the different analytical scales of the methods used. In our study, the polyploidy signal was inferred from block‐level collinearity and median Ks values of syntenic regions, whereas the WGD category in DupGen_finder (Qiao et al. 2019) refers to collinear or segmental duplicate gene pairs defined by syntenic relationships and genomic positions. These pairwise classifications are not equivalent to event‐level estimates of WGD number or timing, which may explain why DupGen_finder‐derived WGD pairs can show bimodal or multimodal Ks distributions, whereas WGDI (Sun et al. 2022) recovers a single dominant block‐level signal consistent with one ancient WGT. We also applied WGDI to the hemp and wild *Cannabis* genomes analysed in previous studies (Gao et al. 2020; Wei et al. 2023), but their Ks distributions were difficult to fit robustly. This may partly explain why those studies relied more heavily on 4DTv patterns when inferring duplication events.

A similarly repeat‐rich genomic architecture (~79%) was observed in our study, consistent with previous reports (Gao et al. 2020; Grassa et al. 2021; Laverty et al. 2019; Lynch et al. 2025; Wei et al. 2023). The recent *Cannabis* pangenome study interpreted this genomic landscape mainly in relation to recent TE activity and its contribution to genome expansion and structural diversification (Lynch et al. 2025). By contrast, our analyses based on gap‐free assemblies suggest that the present genomic landscape is better understood as the combined outcome of the fractionated legacy of the shared *γ* whole‐genome triplication, subsequent small‐scale duplications, and continued repeat‐mediated remodelling (Figure 4g). Moreover, whereas the pangenome study emphasized the overall role of TE activity in shaping the *Cannabis* pangenome, our results indicate a more differentiated pattern of LTR‐RT evolution, with Gypsy lineages showing signals of earlier proliferation and Copia elements showing relatively more recent proliferation (Figure S11). Over longer evolutionary timescales, some early‐expanded lineages may have lost distinguishing features and were therefore classified as ‘Unknown’. Together, these LTR‐RT patterns further support an important contribution of repeat dynamics to the genomic landscape of *Cannabis*.

Comparison of expression estimates obtained by using different assemblies as references showed that apparent expression profiles of *CBDAS*, *THCAS* and *CBCAS* are highly sensitive to reference choice, largely because these loci differ among assemblies in presence, completeness, and structural continuity (Figure S18; Tables S32–S36). This problem is further exacerbated by cross‐mapping among the highly similar synthase regions on chromosome 7 (Laverty et al. 2019). For example, in Cannbio‐2, two highly expressed *CBDAS*‐like genes flanking a sequence gap likely represent a fragmented *CBDAS* locus rather than two independent genes (Figure S18d). These observations indicate that expression‐based inference for cannabinoid synthase genes and graph‐based PAV calls at these TE‐rich tandem loci should be interpreted cautiously, with gene‐centred curation providing a more reliable assessment. The GCS genome, for example, does not provide a reliable basis for evaluating *THCAS* or Y‐linked gene expression because it does not fully represent these loci, although it does resolve the CBDA‐associated region and should support future studies of its regulation and application. The failure to recover major cannabinoid synthase loci in the graph‐derived PAV set should therefore be interpreted cautiously, because these loci reside in TE‐rich tandem arrays that are inherently difficult to resolve reliably using graph‐based PAV inference.

Beyond these assembly‐dependent effects, our phylogenomic and structural analyses also refine the evolutionary interpretation of cannabinoid synthase genes. Previous studies proposed that cannabinoid oxidocyclase genes originated through *Cannabis*‐specific duplication within the BBE‐like family, followed by functional innovation (Van Velzen and Schranz 2021). Our results are consistent with this general framework and further refine it by indicating that a precursor duplication occurred before the split between *Cannabis* and *Humulus*, with one lineage retained on Chr2 and the other subsequently expanding as the Chr7 synthase cluster in *Cannabis*. In *C. sativa* , BBE‐like genes in the two Chr7 synthase regions form dense clusters and share more than 80% amino acid identity with canonical *CBDAS*, *THCAS* and *CBCAS*, whereas Chr2 BBE‐like copies share only about 60% identity, and no canonical synthase candidates were detected on Chr2. Together, these patterns are more consistent with substantial divergence between the two lineages and local expansion of the Chr7 cluster than with a simple recent origin of the Chr7 synthase cluster through inter‐chromosomal duplication from Chr2.

This interpretation is also consistent with previous studies showing that cannabinoid oxidocyclase genes are located within two large syntenic blocks and that *THCAS* and *CBDAS* exhibit deep divergence rather than a simple one‐to‐one orthologous relationship (van Velzen et al. 2018). It is likewise consistent with reports that cannabinoid synthase genes retain relatively low sequence diversity despite residing in a structurally variable genomic region (Lynch et al. 2025). Given that each assembly contains at most one intact copy of *CBDAS* or *THCAS*, whereas *CBCAS* was present in 56% of the assemblies (Figure 6c) (Lynch et al. 2025), gene regions in our study were assigned to *CBDAS*‐ or *THCAS*/*CBCAS*‐associated loci based on structural similarity, domain composition, chromosomal location and phylogenetic relationships among all candidate synthase genes. Together with the phylogenetic placement of *CBDAS* in subclade B1 and *CBCAS*/*THCAS* in the more closely related subclades A2/A1, these patterns are more consistent with local duplication and divergence within the Chr7 cluster than with a simple recent inter‐chromosomal duplication between Chr2 and Chr7. The additional truncated BBE‐like copies in the Chr7 regions further suggest that tandem duplication in this cluster generated a mixture of intact synthases and pseudogenized or partially diverged copies. These findings are also compatible with a scenario in which ancestral cannabinoid oxidocyclases were initially promiscuous, and subsequent duplication and divergence in the *Cannabis* lineage promoted progressive functional specialization into extant cannabinoid synthases (Villard et al. 2025).

More broadly, the GGUR assemblies provide a useful framework for linking structural variation to cannabinoid phenotypes and for dissecting pathway regulation. Future studies integrating gap‐free references with transcriptome, metabolite and functional data should help clarify how genomic variation shapes cannabinoid biosynthesis and may facilitate both breeding and synthetic‐biology applications. The analytical framework developed here may also be useful for other highly heterozygous, repeat‐rich and chemically diverse plant genomes.

## 4 Methods

### 4.1 Plant Material and Genome Sequencing

The sequencing material used in this study was a female GCS accession of *C. sativa* , developed through hybrid breeding from local Chinese cultivars by the Institute of Bast Fibre Crops, Chinese Academy of Agricultural Sciences (Changsha, China). This accession was selected because female inflorescences are the major site of cannabinoid accumulation. In contrast, male plants senesce rapidly after flowering and are therefore less suitable for cannabinoid‐focused metabolite and expression analyses. GCS is a CBDA‐dominant industrial hemp cultivar, containing approximately 4% CBD and no detectable THC under our assay conditions, and is among the higher‐CBDA accessions identified in China to date. The material was propagated hydroponically via clonal cuttings in the institute's greenhouse to ensure consistent genomic material for Hi‐C, PacBio HiFi and CLR, and BGI sequencing. All sequencing samples were derived from clonal progeny of the same female material. The plants were maintained in a greenhouse acclimation room at 25°C under a 16‐h light and 8‐h dark photoperiod.

High‐molecular‐weight genomic DNA was extracted and subsequently fragmented and ligated to platform‐specific adapters to construct sequencing libraries. For long‐read sequencing, both CLR and HiFi sequencing were performed on the PacBio RSII platform. Circular consensus sequencing (CCS) was used to generate HiFi reads, whereas CLR libraries were constructed using a continuous sequencing mode optimized for read length.

To capture full‐length transcript isoforms, Iso‐Seq was performed. The workflow included total RNA extraction, reverse transcription into cDNA, and library construction from full‐length cDNA, enabling comprehensive characterization of transcript diversity and splicing variants. For chromosome‐scale scaffolding, Hi‐C libraries were prepared to preserve three‐dimensional chromatin interactions. Hi‐C data were generated on the BGI M6000 platform.

### 4.2 Estimation of Genome Size and Complexity

To estimate the genome size, heterozygosity and repetitive content of the high‐CBD *Cannabis* cultivar GCS, a genome survey analysis was performed using HiFi reads. Traditional genome size estimation based on short‐read next‐generation sequencing data can be unreliable for the GCS cultivar due to its high repeat content and high heterozygosity resulting from extensive outcrossing. To overcome this limitation, a k‐mer–based approach leveraging HiFi reads was used.

K‐mer frequency distributions were generated using Jellyfish v1.1.12 (Marçais and Kingsford 2011) with multiple k‐mer sizes, including 17‐, 19‐, 21‐ and 23‐mers. The resulting frequency histograms were analysed using GenomeScope2 v2.0.1 (Ranallo‐Benavidez et al. 2020) and findGSE v3.3.1 (Sun et al. 2018) to provide robust and reference‐free estimates of genome size and genome‐wide complexity.

### 4.3 Genome Assembly Pipeline

Genome assembly followed a multi‐step integration strategy. HiFi and Hi‐C reads were first assembled using HiFiasm v0.16.0 (Cheng et al. 2021), which partitions heterozygous sequences paths in the assembly graph to generate two phased outputs. Redundant sequences were then removed with purge_dups v1.2.6 (Guan et al. 2020), yielding two high‐quality draft backbone assemblies. In this framework, haplotype assignment is expected to be most reliable at the level of locally phased contig/phase blocks, where separation is directly supported by read‐level heterozygous information. However, because no trio‐binning or parental sequencing data were available, the long‐range assignment of all phased blocks across scaffolds and chromosomes to complete biological parental haplotypes could not be independently validated. Therefore, the two outputs are interpreted here as phased assemblies (pseudo‐haplotype assemblies) rather than definitive parental genomes.

The detail of genome assembly in this manuscript was as followed. First, we assembled PacBio HiFi and BGI Hi‐C data into two haplotype‐phased backbone assemblies. Chromosome anchoring and manual curation with Hi‐C contact matrices yielded chromosome‐scale scaffolds with only 124 and 144 residual gaps (Figure 1a; Table S3). Second, we generated a genome graph from PacBio CLR data. In total, 102.11 Gb of CLR reads were corrected and trimmed and then assembled, resulting in a 985.43 Mb genome graph with a contig N50 of 1.89 Mb (Figure 1b). Third, we identified breakpoints and validated candidate graph paths for gap filling. This process was categorized into two scenarios (Figure 1c): (i) when contigs flanking a gap were located on the same assembly graph, the gap could be filled directly; (ii) when contigs flanking a gap lay in different graph components, Bandage v0.9.0 (Wick et al. 2015) was employed to traverse the graph, identify a path spanning the gap, and reconstruct the intervening sequence. If a gap spanned multiple candidate paths, contigs absent from the backbone assemblies were assigned to the optimal path through the assembly graph, based on the alignment scores from graph‐associated sequences. Finally, Hi‐C heatmaps were used to validate the gap‐filled assemblies, confirming their accuracy and structural integrity. Gap‐filled regions supported by Hi‐C contact matrices were retained, whereas those lacking Hi‐C support underwent an additional round of gap filling until all gaps were resolved (Figure 1d; Figure S1c,d; Table S4).

The CLR reads were corrected and trimmed using Canu v2.2 (Koren et al. 2017) and then assembled de novo with Flye v2.9.1 (Kolmogorov et al. 2019) to complement and extend regions that were not fully resolved in the HiFi assembly (Bi et al. 2024), thereby improving assembly continuity. Hi‐C data were processed using Juicer v1.6 (Durand et al. 2016) to generate genome‐wide chromatin contact maps, which were subsequently used as input for 3D‐DNA v180922 (Dudchenko et al. 2017) to anchor and scaffold the initial contigs into chromosome‐scale assemblies. Scaffold orientation and ordering were manually curated using Juicebox Assembly Tools through interactive visualization of Hi‐C contact matrices. Gap filling was manually performed using UGENE v52.1 (Rose et al. 2019). Finally, the assembly was polished using NextPolish2 v0.2.1 (Hu et al. 2023) by integrating both long and short sequencing reads to improve base‐level accuracy and overall genome completeness.

### 4.4 Assembly Quality Assessment

The completeness, accuracy, and contiguity of the genome assembly were evaluated using a combination of metrics, including contig N50, scaffold N50, number of gaps and LAI. BUSCO v6.0.0 (Simão et al. 2015) analysis was performed using the core eukaryotic gene dataset (eukaryota_odb10) to assess gene and annotation completeness. The LAI (Ou et al. 2018) was calculated to quantify the completeness and abundance of intact LTR‐RTs, a key indicator of assembly quality in plant genomes.

Standard assembly metrics such as contig N50 and total assembly size were computed using the assembly‐stats toolkit (https://github.com/MikeTrizna/assembly_stats). In addition, Merqury v1.3 (Rhie et al. 2020) was employed for k‐mer‐based, reference‐free evaluation of assembly quality, completeness, consensus accuracy and phasing resolution using a k‐mer size of 21.

To assess the accuracy and completeness of the assembled assemblies, both long reads (PacBio HiFi and CLR) and short reads (generated by the BGI platform) were aligned to the assemblies using minimap2 v2.30 (Li 2021) and BWA‐MEM v0.7.19 (Li and Durbin 2010), respectively. Mapping statistics, including overall alignment rate, effective mapping rate, and genome coverage, were calculated using samtools v1.22 (Danecek et al. 2021).

Assembly continuity and basic metrics, including contig N50, total genome size, and GC content, were evaluated with QUAST v5.3.0 (Gurevich et al. 2013). Gene‐space completeness was examined using BUSCO v6.0.0 (Simão et al. 2015) against the embryophyta_odb10 dataset under both ‘‐‐genome’ and ‘‐‐protein’ modes.

### 4.5 Genome Annotation and Haplotype‐Specific Genes

Genome annotation was performed using a comprehensive multi‐step pipeline (Bi et al. 2024; Xiong et al. 2021). Repetitive sequences were identified using RepeatModeler v2.0.4 (Flynn et al. 2020) and masked using RepeatMasker v4.1.5 (Tarailo‐Graovac and Chen 2009) to generate a hard‐masked genome assembly for subsequent gene prediction. Ab initio gene prediction was carried out on the masked genome using Augustus v3.5.0 (Stanke et al. 2008), SNAP v2.0.5 (Korf 2004), and GeneMark‐EP+ v4.72 (Brůna et al. 2020). Homology‐based annotations were generated using GTH v2.7.1 (https://github.com/chenlianfu/geta), exonerate v2.4.0 (https://github.com/nathanweeks/exonerate) and Miniprot v0.18‐r281 (Li 2023).

Transcriptome‐based annotation was enriched with Iso‐Seq data processed using CCS v6.4.0 (https://github.com/PacificBiosciences/ccs). Additionally, 100 publicly available *Cannabis* transcriptome datasets were retrieved from the NCBI database (https://www.ncbi.nlm.nih.gov/) and assembled using StringTie2 v2.2.2 (Kovaka et al. 2019), generating additional biologically informative transcripts.

All annotation evidence was integrated using EvidenceModeler v2.1.0 (Haas et al. 2008), with weights assigned based on the reliability of each data source. Gene models were further refined using Funannotate v1.8.15 (Palmer and Stajich 2020), incorporating Iso‐Seq data for correction.

In addition, independent annotations of both haplotype assemblies were generated using Helixer v0.3.4 (Holst et al. 2023), and the resulting files were manually curated in IGV v2.16.0 (Robinson et al. 2023) alongside EVM‐derived annotations. Erroneous or implausible gene models were manually reviewed and corrected to ensure the accuracy and completeness of annotation.

Functional annotation of the predicted genes was performed using multiple public databases, including Clusters of Orthologous Groups (COG), Kyoto Encyclopaedia of Genes and Genomes (KEGG), Eukaryotic Orthologous Groups (KOG), the NCBI nonredundant Database (NR), the NCBI nucleotide database (NT) and the Protein Families Database (Pfam).

To identify haplotype‐specific genes and their chromosomal distribution, a genome‐wide homology‐based approach was employed. Pairwise BLAST comparisons were conducted between the two protein sets from the haplotype assemblies, and gene pairs with ≥ 90% sequence identity and reciprocal coverage exceeding 80% of the full‐length protein sequences were defined as allelic. Genes failing to meet these criteria were classified as haplotype‐specific. Here, ‘haplotype‐specific’ genes are defined as annotated genes without an assigned allelic counterpart on the other haplotype under our synteny‐/homology‐based allelic pairing criteria (see Methods).

### 4.6 Telomere and rDNA Identification

Telomeric repeats were identified using Tandem Repeats Finder (TRF) v4.09.1 (Benson 1999) with parameters ‘2 7 7 80 10 50 500’. For each chromosome, 100‐kb windows were defined at both ends, and TRF hits were restricted to these end windows. Adjacent hits separated by ≤ 7 bp were merged to form contiguous arrays of the canonical 7‐bp unit. For each end, the length of the longest array was taken as the telomere length estimate. Ends with an array length ≥ 1000 bp were classified as captured telomeres.

The rDNA loci were identified with Infernal v1.1.5 (Nawrocki and Eddy 2013) using covariance models from the Rfam database (Kalvari et al. 2021): RF01960 (18S/SSU), RF00002 (5.8S), RF02543 (28S/LSU) and RF00001 (5S). Models were retrieved with cmfetch and combined into a single CM file. Each assembly was scanned with cmscan using gathering thresholds (‐‐cut_ga ‐‐rfam ‐‐noali). Hits on the same chromosome and strand were collapsed into loci by merging intervals within 1 kb. Loci containing 18S + 5.8S + 28S were classified as 45S loci; those matching RF00001 were classified as 5S loci. For 45S loci, completeness was classified as Complete (all three components in order), Partial (subset present), or None. For each locus, we reported 1‐based start and end coordinates, locus length (bp), the number of contributing model hits (N_hits), majority strand and majority percentage. Per‐chromosome summaries (total number of arrays, counts of 45S and 5S, total span, and maximum array length) were derived from the locus set and are provided in Table S10.

### 4.7 Epigenomic and Hi‐C Profiling for Putative Centromeric Inference

ChIP‐seq was performed to profile the H3K4me3 modification in *Cannabis sativa* . Fresh young leaves were collected and fixed in 1% formaldehyde under vacuum for 10 min to crosslink protein–DNA interactions, followed by quenching with 0.125 M glycine. Nuclei were isolated and chromatin was sheared to an average size of 200–500 bp using sonication. Immunoprecipitation was carried out using an anti‐H3K4me3 antibody according to the manufacturer's protocol. DNA was purified and libraries were prepared and sequenced on an Illumina platform with 150‐bp paired‐end reads. Clean reads were mapped to the reference genome using Bowtie2 v2.4.2 (Langmead and Salzberg 2012), and peaks were called using MACS2 v2.2.7.1 (Zhang et al. 2008) with default parameters. Genome‐wide enrichment profiles were visualized using IGV v2.16.0 (Robinson et al. 2023) or deepTools v3.5.5 (Ramírez et al. 2014).

Genomic DNA was extracted from young leaves of GCS using a modified CTAB method. DNA quality and quantity were assessed by agarose gel electrophoresis and Qubit fluorometry. For bisulfite conversion, 500 ng of genomic DNA was treated with bisulfite solution. Bisulfite‐converted DNA was used to construct libraries with the TruSeq DNA Methylation Kit (Illumina), and paired‐end sequencing (150 bp) was performed on an Illumina NovaSeq 6000 platform.

Raw reads were quality‐filtered using fastp v1.0.1 (Chen et al. 2018), and clean reads were mapped to the reference genome using Bismark v0.22.3 (Krueger and Andrews 2011) with default parameters. Duplicated reads were removed, and genome‐wide DNA methylation profiles were visualized using IGV v2.16.0 and R v4.3.0 (R Core Team 2019).

Hi‐C sequencing data were quality‐filtered using fastp v1.0.1 (Chen et al. 2018), and high‐quality clean reads were retained for subsequent analyses. The filtered reads were then aligned to both haplotype assemblies using Sentieon BWA‐MEM (Kendig et al. 2019). After removal of PCR duplicates, the resulting BAM files were used for genome‐wide quantification of Hi‐C read coverage. Bedtools coverage (Quinlan and Hall 2010) was used to compute Hi‐C read counts in 500‐kb windows using a 100‐kb sliding step, providing a genome‐wide overview of chromatin contact density.

### 4.8 Genome Collinearity and Genomic Feature Analysis

Syntenic relationships among multiple assemblies were analysed and visualized using NGenomeSyn v1.41 (He et al. 2023). Homology searches were conducted using DIAMOND v0.9.21 in Sensitive mode (Buchfink et al. 2021) to align CDSs and generate pairwise homologous gene sets. The resulting homologous gene pairs and genome annotations (GFF3 and FASTA) were then used as input for NGenomeSyn, with customized parameters (e.g., minimum number of genes per syntenic block and maximum intergenic distance) to define conserved collinear regions and generate synteny plots.

GC content of the genome sequences was calculated using SeqKit v2.10.0 (Shen et al. 2016) through a sliding window analysis with a window size of 500 kb and a step size of 100 kb. This approach enabled genome‐wide quantification of GC content and identification of regional variations in nucleotide composition.

Genome‐wide visualization of structural features was performed using CIRCOS v0.69‐9 (Krzywinski et al. 2009), including chromosome architecture, gene density, TE distribution (specifically Gypsy and Copia elements), GC content, and intergenomic synteny. Gene density and the distribution of Gypsy and Copia elements were calculated using a 500 kb sliding window with a 100 kb step size. Syntenic relationships among assemblies were analysed using MCScanX v1.0.0 (Wang et al. 2024).

In addition, circular plots were independently generated for both haplotype assemblies to display gene density, Gypsy and Copia TE distribution, GC content, synteny, CRM elements, overall TE content, Hi‐C contact maps and LAI, providing an overview of genome architecture and structural variation.

Divergence times among species were estimated using a Bayesian relaxed molecular clock approach, implemented in the MCMCtree program within the PAML package (dos Reis et al. 2019). This method integrates phylogenetic information from single‐copy gene trees with molecular clock models to infer divergence times. Calibration constraints were set with reference to divergence‐time estimates reported in the TimeTree database (TimeTree.org); the crown node of *Malus domestica* and *Prunus mume* was constrained to 34.4–67.2 MYA in the same scale used in our analysis.

### 4.9 Whole‐Genome Duplication and Divergence‐Time Estimation

The whole‐genome duplication history of *Cannabis* was analysed using WGDI v3.12 (Sun et al. 2022). First, species including *Vitis vinifera* (NCBI RefSeq assembly GCF_030704535) and *Buxus austro‐yunnanensis* (NCBI BioProject accession PRJNA808839), together with their reconstructed ancestral karyotypes, were selected. *Humulus japonicus* (PRJDB17941 in the DDBJ database), a close relative of *Cannabis* within *Cannabaceae*, was further used to validate the inference of a single whole‐genome triplication event in *Cannabis*. DIAMOND v0.9.21 (Buchfink et al. 2021) was used for all‐versus‐all BLASTP (Altschul et al. 1997) searches on the longest protein sequence per gene for each genome. The resulting alignments were processed using WGDI, following the standard workflow and default parameters. For estimating WGD and divergence times, we used the block Ks median. This metric is generated by WGDI and helps avoid biases caused by paralogs, providing more robust results. The Ks values for both *Cannabis sativa* (GCS) and *Buxus austro‐yunnanensis* were corrected, following the approach of a previous study (Feng et al. 2024; Song et al. 2021). The correction process was as follows. Although *Cannabis sativa* and *Vitis vinifera* share the same ancestral eudicot polyploidization event, but the Ks peak position for *Cannabis sativa* was shifted to a greater value than that of *Vitis vinifera* , reflecting lineage‐specific rate variation. Therefore, we corrected Ks values before comparing the positions of peaks. The correction factor for *Cannabis* Ks median is defined as λ_cannabis_ = μ_vitis_/μ_cannabis_, where λ_cannabis_ is the scaling factor used for Ks correction, and μ_vitis_ and μ_cannabis_ are the Ks peak positions corresponding to the shared ancestral eudicot polyploidization event in *Vitis vinifera* and *Cannabis sativa* , respectively. For each genome, the central position of the intragenomic block‐Ks peak was estimated by fitting the distribution of block‐wise median Ks values. The fitted central peak positions for *V. vinifera* and *C. sativa* were denoted as μ_vitis_ and μ_cannabis_, respectively. The inference that *V. vinifera* and *C. sativa* share the ancestral eudicot polyploidization event is based on the prior phylogenetic and comparative genomic evidence; the Ks correction was used only to normalize lineage‐specific rate differences for comparative analysis. For Buxus, the Ks median does not show a peak corresponding to the *γ* event. The correction factor for *Buxus* Ks median is λ_Buxus_ = (μ_vitis,cannabis_ × λ_cannabis_)/μ_vitis,Buxus_, where μ values for *Cannabis*‐*Vitis* and *Buxus*‐*Vitis* Ks medians were used to calculate the correction. Additionally, corrected Ks values were computed as Ks_vitis,cannabis_corrected_ = Ks_vitis,cannabis_ × λ_cannabis_ and Ks_Buxus,vitis_corrected_ = Ks_Buxus,cannabis_ × λ_cannabis_ × λ_Buxu*s*_. Ks correction was applied only in the direct comparison among *Vitis vinifera* with *Cannabis sativa* and *Buxus auxtro‐yunnanensis*, because this step was used to account for lineage‐specific substitution‐rate variation when interpreting the focal WGT‐related Ks peaks. Other taxa were retained as reference comparisons and were not included in this pairwise correction procedure.

PacBio HiFi reads were aligned to the corresponding haplotype assemblies (Hap1 and Hap2) using minimap2 v2.30 (Li 2021) with the map‐hifi preset (‐x map‐hifi). Genome coverage was summarized in non‐overlapping 50‐kb windows using a depth profiler, reporting the mean depth per window. Windows with zero depth (e.g., masked or ambiguous regions) were omitted from summary statistics. For each chromosome, we computed the median of its window depths (MedianDepth). To facilitate cross‐chromosome comparison within a haplotype, MedianDepth was normalized by the genome‐wide median window depth of that haplotype, yielding the normalized coverage metric RelToGenome: RelToGenome = MedianDepth_chr_/MedianDepth_genome_.

### 4.10 Gene Family Evolution and Duplication Classification

Comparative genomic analysis was performed with OrthoFinder v2.5.5 (Emms and Kelly 2019) to identify homologous gene clusters across the selected species. The related species were *Ziziphus Jujuba* (NCBI RefSeq assembly GCF_031755915.1) (Yang et al. 2023), *Theobroma Cacao* (GenBank assembly GCA_036851125.1) (Marin Recinos et al. 2024), *Prunus Mume* (NCBI RefSeq assembly GCF_000346735.1) (Zhang et al. 2012), *Parasponia Andersonii* (GenBank assembly GCA_002914805.1) (van Velzen et al. 2018), *Papaver Somniferum* (NCBI RefSeq assembly GCF_003573695.1) (Guo et al. 2018), *Morus Notabilis* (NCBI RefSeq assembly GCF_000414095.1) (He et al. 2013), *Malus Domestica* (NCBI RefSeq assembly GCF_002114115.1) (Daccord et al. 2017), and *Humulus japonicus* (PRJDB17941 in the DDBJ database) (Akagi et al. 2025). Phylogenetic trees were constructed using ASTRAL v5.7.1 (Rabiee et al. 2019) based on single‐copy orthologs, and gene family evolution was modelled using CAFE5 v1.1 (Mendes et al. 2020). Gene families showing significant expansion or contraction (*p* < 0.01) were further analysed for their functional and evolutionary implications.

To classify different types of gene duplications, DupGen_Finder (Qiao et al. 2019) was used to apply using the annotated protein‐coding gene set of each genome. Specifically, the longest protein sequence for each gene, the corresponding chromosomal position information extracted from the GFF3 annotation file, and the all‐versus‐all BLASTP results within each genome were used as input. *Papaver somniferum* was used as an outgroup species for the identification of transposed duplicates. Based on these data, duplicated genes were classified into tandem, proximal, transposed, dispersed and WGD‐derived duplicates. Selection pressure among collinear gene pairs was assessed by calculating Ka/Ks ratios (nonsynonymous/synonymous substitution rates) using KaKs_Calculator v3.0 (Wang et al. 2010). Additionally, the 4DTv correction script (calculate_4DTV_correction.pl.; https://github.com/JinfengChen/Scripts/blob/master/FFgenome/03.evolution/distance_kaks_4dtv/bin/) was used to estimate 4DTv values. These values serve as indicators of neutral mutation accumulation and provide insights into long‐term genome evolution and polyploidization history.

Positive selection analysis was performed on single‐copy orthologs using the branch‐site model implemented in codeml from the PAML package v4.10.7 (Yang 2007). Single‐copy ortholog genes identified by OrthoFinder were used to extract the corresponding CDS sequences from each species. These CDS sequences were aligned and converted into codon‐based alignments, which together with the species phylogenetic tree, were used as input for codeml. In the branch‐site model, the *Cannabis sativa* lineage was designated as the foregroup branch, whereas all remaining branches were treated as background branches. Likelihood ratio tests (LRTs) were performed by comparing the alternative model, which allows a class of sites under positive selection on the foreground branch, with the null model, in which the corresponding site class was fixed at ω = 1. The test statistic was calculated as 2ΔlnL = 2 × (lnL_alternative__−lnL_null_), and *p* values were obtained based on a mixed distribution of 0 and *χ* ^2^ with one degree of freedom. Genes with significant LRT results were considered candidate positively selected genes. Bayes Empirical Bayes (BEB) analysis was further used to identify specific codon sites under positive selection.

### 4.11 LTR‐RT Identification and Classification

In this study, putative LTR‐RTs were systematically identified across the genome using two de novo structure‐based tools: LTR_FINDER v1.3 (Ou and Jiang 2019) and LTRharvest v1.6.7 (Ellinghaus et al. 2008). These programs scanned the assembled genome to detect candidate LTR elements, producing SCN files that record the genomic coordinates, sequence lengths, and structural features of predicted LTR‐RTs. The SCN outputs were subsequently processed by LTR_retriever v3.0.4 (Ou et al. 2018), which integrates and refines candidate predictions to identify high‐confidence intact LTR‐RTs.

Following LTR‐RT identification, intact elements were extracted and classified using TEsorter v1.4.7 (Zhang et al. 2022), which assigns LTR‐RTs to specific lineages based on conserved protein domains. This domain‐based classification allowed the systematic categorization of intact LTR‐RT elements into major superfamilies and subclades.

The *Cannabis* genome assemblies used in this study were retrieved from public databases. The Yushe genome was obtained from the China National Center for Bioinformation (CNCB; https://bigd.big.ac.cn/gwh) under accession number GWHCAYE00000000. The Cs10 (GCF_900626175.2), JL (GCA_013030365.1), Cannbio‐2 (GCA_016165845.1), Purple Kush (GCA_000230575.5), Pink Pepper (GCA_029168945.1), ANC‐1 (GCA_051911675.1), ANC‐2 (GCA_051911655.1), Hawthorne OG‐1 (GCA_051911705.1), Hawthorne OG‐2 (GCA_051911645.1), Sri Lankan (GCA_051911735.1) and Finola (GCA_003417725.2) genomes were downloaded from the NCBI database.

The genomic distribution of Gypsy and Copia transposable elements was characterized based on annotations generated by RepeatMasker v4.1.5 (Tarailo‐Graovac and Chen 2009). For each chromosome, the abundance of elements was quantified using a sliding window approach with a window size of 500 kb and a step size of 100 kb. This analysis allowed the identification of both local enrichment patterns and large‐scale distribution trends of LTR retrotransposons across the genome.

### 4.12 Intact LTR Phylogenetic and PCA Analysis

The classified intact LTR‐RT sequences were then retrieved using the SeqKit subseq command and aligned with MAFFT v7.526 (Katoh and Standley 2013) to generate multiple sequence alignments. Phylogenetic reconstruction was performed using IQ‐TREE v2.10.0 (Minh et al. 2020), and the resulting trees were visualized with FigTree v1.4.2 (Rambaut 2018) and the ggtree (Xu et al. 2022) package in R. PCA was further conducted using the adegenet package (Jombart 2008) to explore the genetic variation and clustering patterns among intact LTR‐RTs.

### 4.13 Orthogroup Identification and Domain‐Based Phylogenetic Analysis of Cannabinoid Synthases

Genes containing the protein domains FAD_binding_4 (PF01565) and BBE_like (IPR012951) were identified from InterProScan v5.7.3 (Jones et al. 2014) annotations of nine eudicot species, including the eight species used in the gene family evolution analysis and *Humulus japonicus* . Orthogroups were computed using OrthoFinder v2.5.5 (Emms and Kelly 2019), and domain composition was mapped using custom scripts. Orthogroups containing these domains were extracted, and all members were retained for further analysis. A maximum‐likelihood tree was constructed with IQ‐TREE v2.10.0 (Minh et al. 2020) using aligned protein sequences and was annotated by domain class and species. Chromosomal positions of *Cannabis* genes were extracted from the GCS reference genome to assess genomic colocalization and duplication patterns.

Based on domain annotations in the eight *Cannabis sativa* assemblies, gene models carrying both an FAD‐binding domain and a BBE‐typical domain were identified for subsequent analyses. For each retained gene, the regions corresponding to the FAD‐binding and BBE‐typical domains were extracted and concatenated, and the resulting protein sequences were aligned using MAFFT with default parameters and trimmed with trimAl v1.5.rev0 (Capella‐Gutiérrez et al. 2009) to remove poorly aligned sites. A maximum likelihood phylogenetic tree was then inferred using IQ‐TREE with automatic model selection and 1000 ultrafast bootstrap replicates. Multiple sequence alignments were visualized in Jalview v1.8.3 (Waterhouse et al. 2009), which was also used to examine non‐conserved residues within the FAD‐binding and BBE‐typical domain regions.

### 4.14 Identification of *CBCAS* / *CBDAS* / *THCAS* Homologues and Genomic Spacing

Putative homologous sequences of *CBDAS*, *CBCAS* and *THCAS* were identified through protein‐guided homology searches against each assembled genome using Miniprot v0.18‐r28135 and TBLASTN v2.13.0. Candidate loci were curated based on conserved domain architecture and sequence completeness. Assemblies with low contiguity and annotation quality (PK, Finola and JL) were excluded from this analysis. The Cs10 assembly, although of lower overall quality, was retained because its scaffolds are anchored by a linkage map, allowing approximate estimation of intergene distances between the *CBDAS* and *THCAS* arrays.

### 4.15 Topology Specification and Tree‐Species Tree Reconciliation

To formalize the alternative evolutionary relationships among the oxidocyclase lineages, we defined a compact, Newick‐style shorthand for ingroup topologies. Parentheses denote clades, commas separate sister lineages, and an ellipsis (‘…’) indicates additional paralogs not enumerated individually. We use H for the *Humulus japonicus* copy, C2 for the *Cannabis sativa* copy on chromosome 2, and C7 for the *Cannabis* tandem cluster on chromosome 7 (potentially comprising multiple paralogs; when referred to collectively, we write C7…). All topologies are rooted with *Parasponia andersonii* as the outgroup, which is omitted from the shorthand for brevity. Under this notation, the three mutually exclusive hypotheses considered for the ingroup are:

$$\text{T1}=\left(\left(\text{C2},\left(\text{C7}\ldots \right)\right),H\right)$$

$$\text{T2}=\left(\left(\text{C7}\ldots ,\left(\text{C2}\right)\right),H\right)$$

$$\text{T3}=\left(\left(H,\text{C2}\right),\text{C7}\ldots \right)$$

In words, T1 and T2 place *Humulus* as sister to a combined *Cannabis* clade, with C2 and C7… as each other's sisters; T3 groups *Humulus* with C2, with C7… sister to that pair. Note that T1 and T2 are identical as ingroup topologies; they differ only as alternative mechanistic scenarios (i.e., ‘2 → 7’ versus ‘7 → 2’ intragenomic copying) that map to the same topology (H,(C2,C7…)). Accordingly, the Approximately Unbiased (AU) topology test effectively compares T3 against the topology shared by T1 and T2 using site‐wise log‐likelihoods computed from the curated alignment. *P. andersonii* is included in likelihood calculations solely to fix the root.

For clarity in the main text and figure captions, we retain the above Newick strings and the symbol definitions (H, C2, C7…) at their first occurrence, and thereafter refer to the hypotheses as T1, T2 and T3.

We performed gene‐tree/species‐tree reconciliation with Treerecs v1.2 (Comte et al. 2020) under a duplication‐loss (DTL) framework using the rooted species tree and the AU‐supported gene tree, whose root was fixed a priori with *Parasponia andersonii* as the outgroup. Treerecs was run without re‐rooting or branch‐support collapsing so that the reconciliation would not alter the inferred topology or support of the input gene tree.

### 4.16 Graph‐Based Pan‐Genome Construction and PAV Identification

To construct a graph‐based pan‐genome and identify PAV in *Cannabis sativa* , we selected seven representative accessions: GCS (Hap1, Hap2), Cannbio‐2, Cs10, Finola, JL, Purple Kush and Yushe. Whole‐genome assemblies of each accession were aligned using minigraph v0.21‐r606 (Li et al. 2020) with the ‘‐x asm’ preset to construct a variation‐aware reference graph, using GCS_Hap1 as the initial backbone. Assemblies were iteratively integrated into the graph in an order determined by assembly quality and contiguity.

Following graph construction, we performed pairwise mapping of each accession's assembly back to the graph using minigraph's ‘‐cx asm’ mode to extract the presence/absence status of each variant segment. The resulting PAV set was filtered to retain only nonredundant segments ≥ 50 bp in length that did not overlap annotated centromeric or pericentromeric regions. PAV coordinates were mapped back to GCS_Hap1 for visualization along reference chromosomes. A pangenome graph was constructed using the assembled genomes, and graph segments present in the GCS_Hap1 backbone but absent in one or more other accessions were summarized as backbone‐relative sequence variation along the reference chromosomes. Because this metric is reference‐dependent, it was used only to describe regional sequence variation in the genomic context of pathway loci, and was not used to infer gene‐level presence/absence of cannabinoid synthase genes.

For most cannabinoid‐biosynthetic pathway genes, copy number variation was summarized from the number of genes assigned to the corresponding OrthoFinder orthogroups. For the orthogroups associated with *CBDAS*, *THCAS* and *CBCAS*, the OrthoFinder‐identified member genes were further curated in each assembly to assess coding‐sequence integrity. In this step, we distinguished intact copies from non‐intact/pseudogene‐like copies, while the lack of an orthogroup member in a given assembly was treated as absence. As sequences lacking an initiation codon had already been excluded before OrthoFinder analysis, this evaluation was focused primarily on intactness among the retained synthase homologues.

### 4.17 Transcriptome Analysis and TE‐Expression Integration

To investigate the expression patterns of cannabinoid biosynthesis‐related genes, we analysed transcriptome data from floral‐stage leaves of four *Cannabis sativa* varieties: PK (SRR352195, SRR352196), JL (SRR10193436, SRR10193437, SRR10193438, SRR10193439), Cannbio‐2 (SRR10600874, SRR10600875) and Finola (SRR351932, SRR351933). These RNA‐seq datasets were retrieved from the NCBI Sequence Read Archive. In addition, transcriptome sequencing of floral‐stage tissues was performed for the GCS variety at peak flowering. We focused on seven key enzyme‐coding genes involved in the cannabinoid biosynthetic pathway: *OAC*, *OLS*, *AAE1*, *aPT4*, C*BDAS*, *THCAS* and *CBCAS*. RNA‐seq reads were aligned to different reference genomes using HISAT2 (Kim et al. 2019), and transcript abundance was quantified with StringTie2 (Kovaka et al. 2019). Gene expression levels were normalized and reported as TPM.

To enable cross‐cultivar comparisons, TPM values were log_2_‐transformed, and density curves were plotted using the ggplot2 (Gómez‐Rubio 2017) package in R. The peak value of each distribution was used as a reference for normalization, and the expression levels of the target genes were compared by evaluating their relative position and fold change against the peak. This approach was used to assess expression differences across RNA‐seq datasets derived from distinct genetic backgrounds or mapped to different reference genomes.

Additionally, to more accurately evaluate the expression of homologous genes encoding cannabinoid pathway enzymes, transcriptome reads from each cultivar were aligned to their respective reference genomes. TPM values were recalculated accordingly to provide more precise and comparable quantification of homologous gene expression across genotypes.

To investigate the relationship between the expression of genes in the cannabinoid biosynthetic pathway and TE insertion, we first catalogued all homologues of the pathway genes. We then integrated Extensive de‐novo TE Annotator (EDTA) v2.21 (Ou et al. 2019) annotations to quantify the number of TE insertions associated with each homologue and, together with gene expression data, to evaluate how TE insertion burden correlates with transcript abundance.

## Author Contributions

J.Y., D.L., L.L. designed the research, J.Y. supervised the research. L.Z. provided the plant material for sequencing. J.Z. conducted the bioinformatic analysis. J.Z., C.Q., and Z.L. organized the data and graph. J.Z. and C.Q. wrote the manuscript. J.Y., L.Z., A.B., T.L., M.L., and J.Z. revised the manuscript.

## Funding

This work was supported by Guangdong S&T Program (Grant No. 2024B1111160007), the National Natural Science Foundation of China (Grants No. 32425011, 32488302 and 31871674), the Agricultural Science and Technology Innovation Program (ASTIP, No. CAAS‐CSIAF‐202302), and the New Cornerstone Science Foundation through the XPLORER PRIZE.

## Disclosure

Code availability: The scripts used in this study are freely available on GitHub at https://github.com/Jiangjiangzhang6/cannabis‐sativa‐genome.

## Conflicts of Interest

The authors declare no conflicts of interest.

## Supporting information


**Table S1:** Summary statistics of *Cannabis* genome generated GCS sequencing data used in the genome assembly.
**Table S2:** Evaluation of genome size and heterozygosity.
**Table S3:** Number of gaps per chromosome in the scaffolded GCS haplotype genomes.
**Table S4:** Summary statistics for scaffolding and gap‐filling of the two GCS haplotype genomes.
**Table S5:** Summary of the two haplotype genomes.
**Table S6:** Evaluation of genome completeness using BUSCO.
**Table S7:** Chromosome‐wise distribution of duplicated BUSCOs in the two haplotype assemblies.
**Table S8:** Summary of telomeric arrays at chromosome ends in the two haplotypes.
**Table S9:** Genomic distribution and characteristics of 45S and 5S rDNA arrays in the haplotype‐phased *Cannabis sativa* assemblies.
**Table S10:** Haplotype‐phased chromosome‐level statistics for 45S/5S rDNA array counts and lengths.
**Table S11:** Statistics of the HiFi reads mapped to the two haplotype genomes.
**Table S12:** Statistics of the CLR reads mapped to the two haplotype genomes.
**Table S13:** Statistics of the NGS reads mapped to the two haplotype genomes.
**Table S14:** Chromosome‐level HiFi coverage of the two haplotype assemblies: median read depth and normalized coverage.
**Table S15:** HiFi reads‐based k‐mer estimates for chromosomes of two haplotype genomes.
**Table S16:** HiFi reads‐based estimates for the two *Cannabis sativa* haplotype genomes.
**Table S17:** Comparative analysis of assembly metrics across diverse *Cannabis* genomes.
**Table S18:** Intact LTR‐RT content and LAI in two haplotype genomes.
**Table S19:** Haplotype‐specific genes and one‐to‐one allelic gene pairs between the two haplotype genomes.
**Table S20:** Distribution of haplotype‐specific (unique) genes across chromosomes in the two haplotype genomes.
**Table S21:** Genes with rapidly changing copy numbers.
**Table S22:** Summary of repetitive sequences, duplicated genes and lengths of repetitive, genic and intergenic regions on different chromosomes.
**Table S23:** Syntenic depth distribution between *Cannabis sativa* and *Humulus japonicus*.
**Table S24:** Syntenic classification of Non‐1:1 gene pairs between *Cannabis sativa* and *Humulus japonicus* (tandem vs non‐tandem).
**Table S25:** Raw and intact LTR‐RTs numbers across different *Cannabis sativa* genomes.
**Table S26:** Raw and intact LTR‐RT lengths in different *Cannabis sativa* genomes.
**Table S27:** Numbers of various duplicated genes in different *Cannabis sativa* genomes.
**Table S28:** HMMER‐detected *CBDAS*, *THCAS* and *CBCAS* homologues showing amino acid residues at positions corresponding to His292 and Tyr417.
**Table S29:** Positively selected genes in *Cannabis sativa*.
**Table S30:** OGs of the cannabinoid pathway in seven Cannabis genomes and the number of PAV insertions.
**Table S31:** Transposable element insertion counts in intronic and 2‐kb flanking regions, and corresponding expression levels of *CBDAS*, *THCAS* and *CBCAS* across seven *Cannabis sativa* accessions.
**Table S32:** Expression of *CBDAS*/*THCAS* tandemly duplicated genes in the Hap1 reference genome.
**Table S33:** Expression of *CBDAS*/*THCAS* tandemly duplicated genes in the Hap2 reference genome.
**Table S34:** Expression of *CBDAS*/*THCAS* tandemly duplicated genes in the Yushe reference genome.
**Table S35:** Expression of *CBDAS*/*THCAS* tandemly duplicated genes in the Cannbio‐2 reference genome.
**Table S36:** Expression of *CBDAS*/*THCAS* tandemly duplicated genes in the Cs10 reference genome.
**Table S37:** TE ratio of eight *Cannabis sativa* genomes and seven related species.
**Table S38:** Distribution of secondary metabolite biosynthetic gene clusters in the two haplotype‐phased GCS genomes.

**Figure S1:** Assembly and genomic landscape of two haplotype‐phased *Cannabis sativa* genomes. (a) GenomeScope k‐mer distribution of 21‐mers for *C. sativa*. The x‐axis represents k‐mer coverage (log scale), and the y‐axis indicates the k‐mer frequency (log scale). The observed k‐mer spectrum (blue) is fitted by the full model (black), with separate curves representing unique (yellow) and erroneous (orange) k‐mers. Vertical dashed lines mark key model parameters, including the heterozygous peak, and homozygous peak, low‐copy repeat peak, and high‐copy repeat peak, respectively; the red dashed line marks the model‐defined coverage threshold for filtering high‐frequency or aberrant k‐mers. The model estimates a genome size of 738.08 Mb with a heterozygosity of 1.08% and a sequencing error of 0.26%. (b) K‐mer frequency distribution of 21‐mers analysed using findGSE. The observed k‐mer distribution (grey) was fitted using a genome size estimation model (red), with a secondary curve (blue) representing an intermediate fitting step. The dashed cyan line indicates the theoretical peak for diploid homozygous regions. The model estimated a genome size of 738.45 Mb with a corrected k‐mer coverage of 23.17×, a heterozygosity rate of 6.4%, and a repetitive content of 387.84 Mb (53% of the genome). A fitted genome size based solely on filtered k‐mers yielded 452.84 Mb at 34.94× k‐mer coverage. (c, d) Final Hi‐C contact heatmaps of *Cannabis sativa* assemblies for Haplotype 1 (c) and Haplotype 2 (d). Chromosome identifiers are shown along both axes, corresponding to scaffolded pseudochromosomes. Interaction intensity is colour‐coded on a log scale from yellow (strong) to purple (weak), with prominent diagonal blocks indicating consistent intra‐chromosomal contacts and supporting the structural accuracy of the final assemblies. (e, f) Circular representation of genomic and epigenomic features for *Cannabis sativa* Haplotype 1 (e) and Haplotype 2 (f). Tracks from outer to inner represent: I, chromosomes; II, gene density; III, Gypsy LTR retrotransposons; IV, Copia LTR retrotransposons; V, GC content; VI, CRM elements; VII, overall TE density; VIII, Hi‐C contact intensity; IX, LTR Assembly Index (LAI). All features are calculated in sliding windows along each chromosome.
**Figure S2:** Functional annotation and evolutionary analysis of gene families in the haplotype‐phased *Cannabis sativa* genomes. (a, b) UpSet plots show the distribution of genes functionally annotated in eight databases for Haplotype 1 (a) and Haplotype 2 (b), respectively. Databases include NR, KOG, KEGG, NT, GO, Swiss‐Prot, Pfam and COG. The upper bar plot shows the number of genes annotated in each specific combination of databases, with the corresponding database combinations in the matrix below. (c) Summary of gene annotation across multiple databases for Haplotype 1 and Haplotype 2. Stacked bar plots depict the number of genes annotated by one to eight functional databases in Haplotype 1 (left) and Haplotype 2 (right). Each colour represents the number of databases supporting a given gene annotation, from dark blue (one database) to orange (eight databases). Segment heights are scaled to the number of genes, and the totals are labelled above the bars. (d) Phylogenetic tree inferred from single‐copy orthologs among *C. sativa* and seven other species. Red numbers denote the divergence‐time range for each node, while green numbers represent the mean divergence time (MYA). The scale bar (10.0) represents 10 million years. (e, f) Gene Ontology (GO) enrichment analysis of significantly expanded (e) and contracted (f) gene families in *C. sativa*.
**Figure S3:** Centromere identification across the Haplotype 1 genome based on integrated genomic and epigenomic features. (a) Genomic landscape of ten *Cannabis sativa* chromosomes showing gene density, Gypsy and Copia transposable element density, CRM element distribution, GC content, and H3K4me3 ChIP‐seq signal. Gene density and TE content were calculated in non‐overlapping 500‐kb windows. H3K4me3 enrichment and CRM elements were shown as genome tracks. Brown dashed lines indicate the putative centromeric region, inferred by comparative analysis of gene density, GC content, and the distribution of Gypsy, Copia, and CRM elements. (b–k), Detailed views of centromeric regions on each chromosome (Chr01 to Chr0X), marked by black dashed lines. Centromeres were identified by local enrichment of CRM and Gypsy elements, reduced gene density, increased GC content, and depletion of H3K4me3 signal. These features collectively support the structural and epigenetic delineation of centromere positions across the *C. sativa* genome.
**Figure S4:** Centromere identification across the Haplotype 2 genome based on integrated genomic and epigenomic features. (a) Genomic landscape of ten *Cannabis sativa* chromosomes showing gene density, Gypsy and Copia transposable element density, CRM element distribution, GC content and H3K4me3 ChIP‐seq signal. Gene density and TE content were calculated in non‐overlapping 500‐kb windows. H3K4me3 enrichment and CRM elements were shown as genome tracks. Brown dashed lines indicate the putative centromeric region, inferred by comparative analysis of gene density, GC content, and the distribution of Gypsy, Copia, and CRM elements. (b–k) Detailed views of centromeric regions on each chromosome (Chr01 to Chr0X), marked by black dashed lines. Centromeres were identified by local enrichment of CRM and Gypsy elements, reduced gene density, increased GC content, and depletion of H3K4me3 signal. These features collectively support the structural and epigenetic delineation of centromere positions across the Haplotype 2 genome of *C. sativa*.
**Figure S5:** Multi‐feature genomic landscape and centromere identification across chromosomes of *Cannabis sativa* Haplotype 1. (a–j) Line plots showing the distribution of genomic and epigenomic features along each chromosome of *C. sativa* Haplotype 1. Features include gene density, H3K4me3 histone modifications, GC content, and transposable elements (Copia, Gypsy, and centromeric retrotransposons, CRM). Putative centromeric regions were inferred based on peaks in CRM density; reductions in gene and H3K4me3 signals, and localized shifts in GC content, and are marked by vertical dashed lines. Chromosomes Chr01 to Chr0X are shown in panels (a)–(j), respectively. All features were calculated in 500‐kb sliding windows with a 100‐kb step size across chromosomes.
**Figure S6:** Multi‐feature genomic landscape and centromere identification across chromosomes of *Cannabis sativa* Haplotype 2. (a–j) Line plots illustrating the distribution of genomic and epigenomic features along each chromosome of *Cannabis sativa* Haplotype 2. Features include gene density, H3K4me3 histone modifications, GC content, and transposable elements (Copia, Gypsy, and centromeric retrotransposons, CRM). Putative centromeric regions were inferred based on peaks in CRM density, reductions in gene and H3K4me3 signals, and local changes in GC content; they are marked by vertical dashed lines. Chromosomes Chr01 to Chr0X are shown in panels (a)–(j), respectively. All features were calculated in 500‐kb sliding windows with a 100‐kb step size across chromosomes.
**Figure S7:** Macrosyntenic correspondence between *Cannabis sativa* and *Vitis vinifera*. (a) Genome‐wide macrosyntenic comparison between *C. sativa* and *V. vinifera*. Coloured short line segments indicate collinear gene pairs, with colours representing synonymous substitution rates (Ks) of the corresponding anchors. Pink shaded blocks highlight the major chromosome‐level syntenic correspondences emphasized for visualization. (b–g) Enlarged views of representative syntenic regions highlighted in panel (a), showing repeated chromosome‐scale correspondences between the two genomes. These local and genome‐wide patterns are consistent with the retention of ancient polyploid‐derived syntenic relationships in *C. sativa*.
**Figure S8:** Macrosynteny between *Cannabis sativa* and *Buxus austro‐yunnanensis*. (a) A Ks‐coloured macrosynteny heatmap showing homologous relationships between *C. sativa* chromosomes and *B. austro‐yunnanensis* linkage groups (LGs). Pink‐shaded blocks represent regions of conserved synteny. (b–g) Enlarged views of selected syntenic blocks highlighted in (a), displaying dot plots of homologous gene pairs between specific chromosome–LG pairs. Each dot is coloured by synonymous substitution rate (Ks).
**Figure S9:** Synteny analysis and whole‐genome duplication (WGD) signals in *Cannabis sativa*. (a) Dot plot showing self‐synteny among the ten *Cannabis sativa* chromosomes. Homologous gene pairs are coloured by synonymous substitution rate (Ks), with red indicating recent duplications and purple representing ancient events. Red‐shaded blocks indicate high‐confidence syntenic regions. Red arrows mark representative duplication events: (a) intrachromosomal duplications within Chr05 and Chr01; (b) interchromosomal syntenic blocks between Chr01 and Chr0X, and between Chr01 and Chr06; (c) collinear blocks between Chr08 and Chr03, as well as between Chr08 and Chr04; (d) segmental duplications involving Chr04 and Chr08, as well as Chr04 and Chr03. The Ks distribution reveals evidence of both ancient wholegenome duplication remnants and more recent, localized duplication events. (b) Chromosome‐scale synteny between *Cannabis sativa* and *Humulus lupulus* coloured by synonymous divergence. Each dot represents a syntenic anchor gene pair plotted according to its chromosomal coordinates in *C. sativa* (vertical axis) and *H. lupulus* (horizontal axis). Grey grid lines indicate chromosome boundaries. Dot colours show Ks values as indicated by the scale bar, highlighting largely 1:1 collinear relationship between chromosomes of the two species. (c, d) Dot plots illustrating synteny between *Cannabis sativa* (Y‐axis) and ancestral genomes reconstructed from *Vitis vinifera* and *Buxus austro‐yunnanensis* (X‐axis), respectively. Each dot represents a homologous gene pair, with darker regions marking duplicated ancestral segments retained in the *Cannabis* genome. Red indicates high syntenic conservation, while blue denotes lower homology. These plots highlight conserved ancestral genomic blocks, extensive chromosomal rearrangements, and signatures of ancient polyploidy events.
**Figure S10:** Comparative divergence patterns among duplicate‐gene classes and major LTR superfamilies. (a) Comparison of among‐genome divergence, expressed as coefficient of variation (CV, %), between the Copia and Gypsy superfamilies across *Cannabis* genomes. Each point represents one genome. Boxplots show the median and IQR, and whiskers extend to 1.5 × IQR. The *p* value was calculated using a two‐sided Wilcoxon rank‐sum test. (b) Median Ka/Ks values of duplicate gene pairs classified by duplication mode, with vertical bars indicating the interquartile range (IQR). Numbers above each point indicate the number of gene pairs in each category. DSD, dispersed duplicates; PR, proximal duplicates; TD, tandem duplicates; TRD, transposed duplicates; WGD, whole‐genome‐duplication‐derived duplicates. (c) Pairwise differences in median Ka/Ks among duplication classes. Each cell shows Δmedian, calculated as the median Ka/Ks of the column group minus that of the row group. (d) Cliff's delta effect sizes for the same pairwise comparisons shown in panel (b). Positive values indicate relatively higher Ka/Ks values in the column group than in the row group, whereas negative values indicate the opposite. Qualitative effect‐size categories are shown within cells.
**Figure S11:** Genomic distribution, classification, and divergence profiles of LTR retrotransposons across eight *Cannabis sativa* genomes. (a) Chromosomal distributions of genes and transposable elements (TEs) along Hap1 chromosomes. Gypsy and Copia superfamilies are shown in the upper and middle panels, respectively, and DNA elements are shown in the lower panel. The names of individual TE subfamilies are labelled on the right. Chromosome identifiers are displayed at the top, and dashed vertical lines indicate inferred centromere positions. (b) Principal component analysis (PCA) of intact LTR retrotransposon (LTR‐RT) sequences from the Hap1 genome. Subfamilies are colour‐coded. Gypsy and Copia elements are distinguished by shape (triangles and circles, respectively). Ellipses represent 95% confidence intervals for each superfamily. (c) Maximum‐likelihood phylogenetic tree of intact LTR‐RTs from the Hap1 genome. Subfamilies are colour‐coded and annotated along the x‐axis. The Gypsy and Copia superfamily regions are indicated below the tree. (d) Boxplots showing sequence divergence from consensus (%) across Gypsy, Copia, and Unknown LTR‐RT superfamilies in eight *C. sativa* assemblies. Subfamily classifications are shown in consistent colours across panels, while in the final group (‘All’), divergence values across all LTR‐RTs are coloured corresponding to individual genomes.
**Figure S12:** Phylogenetic analysis of intact LTR retrotransposons across *Cannabis sativa* genomes. (a–g) Maximum‐likelihood phylogenetic trees of intact LTR‐RTs (Gypsy and Copia) for seven *C. sativa* assemblies: (a) Hap2, (b) Yushe, (c) Pink, (d) Cs10, (e) PK, (f) Hawthorne and (g) EH23a. Clades are colour‐coded by LTR‐RT lineage, classified based on conserved domain annotation. The distribution and structure of these clades reflect the diversity and amplification histories of retrotransposons within each genome.
**Figure S13:** Principal component analysis of intact LTR‐RT elements across multiple *Cannabis sativa* genomes. (a–g) PCA plots of intact long terminal repeat retrotransposons (LTR‐RTs) from seven *Cannabis sativa* assemblies. For each panel genome, the left subplot shows PC1 versus PC2 and the right subplot shows PC1 versus PC3. Data points represent individual intact LTR‐RT insertions, coloured by transposable element subfamily as indicated in the legend. Ellipses denote clusters of related elements. The paired presentation of PC1‐PC2 and PC1‐PC3 highlights genome‐specific differences in intact LTR‐RT differentiation and shows that the convergence patterns of Gypsy and Copia elements vary among genomes. Genome names are indicated next to each pair of plots, and colours correspond to LTR‐RT subfamilies as shown in the common legend.
**Figure S14:** Comparative genomic characterization of intact LTR retrotransposons across eight *Cannabis* genomes. (a) Total lengths of intact (red) and raw (blue) LTR retrotransposons (LTR‐RTs) across genomes. For each genome, LTR‐RT length is shown for various categories including all LTRs, unknown LTRs, Copia LTRs and Gypsy LTRs. The number within the blue bars indicates the total (raw) LTR‐RT length, and the number to the right of each bar indicates the length of intact LTRs. The value in parentheses indicates the ratio of intact to total LTR‐RT length. Different genome assemblies are represented by bars in distinct colours. (b) Distribution of intact LTR‐RT counts across different genomes. Categories include total intact LTRs, unknown intact LTRs, intact Copia LTRs and intact Gypsy LTRs. Numbers to the right of each bar indicate the count within each category. Genome assemblies are represented by different colours. (c) Genome‐wide chromosomal distribution of LAI values for eight *C. sativa* genomes. LAI was calculated using a 3 Mb window with a 300 kb step size. Chromosomes are aligned based on homologous groups defined by the GCS reference, and original chromosome numbering from other assemblies is ignored. Different line colours represent different *C. sativa* assemblies. (d–f) Distribution of intact LTR‐RT elements by subfamily across the eight genomes (ordered from left to right within each subfamily: Hap1, Hap2, Yushe, Pink, Cs10, PK, Hawthorne, EH23a). Different colours represent distinct LTR subfamilies. Panel (d) shows the number of intact elements per subfamily; due to large differences in copy number, y‐axis ranges differ among subfamilies (250–300, 800–1200 and 1200–2000). The panel (e) displays the total length of intact LTRs per subfamily. Panel (f) shows the average length of intact LTRs per subfamily. Missing bars indicate the absence of the corresponding subfamily in a given genome.
**Figure S15:** Variation in the number of LTR retrotransposons across *Cannabis sativa* genomes. Bar plots depicting the total number of raw LTR‐RT annotated in eight *Cannabis sativa* genomes: Hap1, Hap2, Yushe, EH23a, Cs10, Pink Hawthorne and PK. For each genome, separate bars show the total number of LTRs (‘All’) and the counts for unclassified (Unknown), Copia and Gypsy subfamilies. Genomes are colour‐coded based on haplotype or variety, with total LTR counts (All) indicated at the end of each bar.
**Figure S16:** Gene duplication patterns and chromosomal homology across different *Cannabis sativa* genomes. (a) Counts of genes assigned to whole‐genome duplication (WGD), tandem duplication (TD), proximal duplication (PR), dispersed duplication (DSD), and transposed duplication (TRD) in Hap1, Hap2, Cs10, PK, Yushe, EH23a, Pink and Hawthorne2 genomes. Bars are colour‐coded by assembly. (b) Gene numbers for homologous chromosome pairs of the two GCS haplotypes. Within each chromosome‐specific colour group, four adjacent bars represent Hap1 homologous genes, Hap2 homologous genes, Hap1 genes and Hap2 genes from left to right. (c–e) Violin plots showing, for the five duplication categories in each genome, the distributions of Ka/Ks ratios (c), Ka values (d) and Ks values (e). Different duplication categories are colour‐coded, and each genome is displayed in a separate plot.
**Figure S17:** Phylogenetic tree of homologues of *CBDAS*, *THCAS* and *CBCAS* across representative plant species. (a) Maximum‐likelihood phylogeny of protein sequences homologous to *CBDAS*, *THCAS* and *CBCAS* from representative plant species. Branches are coloured by species. The red dashed box indicates the clade containing cannabinoid synthase homologus genes. (b), Subtree corresponding to the clade in (a), with gene identifiers shown for *CBDAS*, *THCAS*, *CBCAS* and related homologues from *Cannabis sativa*.
**Figure S18:** Expression profiles of cannabinoid synthase genes and associated pathways across different *Cannabis sativa* genomes. (a–e), Heat maps of log_2_(TPM) expression for homologous genes in the *CBDAS* and *THCAS/CBCAS* regions using the Hap1 (a), Hap2 (b), Yushe (c), Cannbio‐2 (d) and Cs10 (e) as reference assemblies. Reference‐assembly IDs are shown above each matrix. Accession IDs are plotted at the bottom of each corresponding column. Gene IDs are listed on the left. The row labelled ‘log_2_(TPM)’ gives the TPM value at the peak of the kernel‐density curve of log_2_(TPM) for all genes in that accession. Rows above ‘log_2_(TPM)’ contain homologous genes from the *THCAS/CBCAS* region, whereas rows below contain homologous genes from the *CBDAS* region. Cell is coloured according to log_2_(TPM), from white (low) to red (high). The heat map to the right converts each gene's TPM value to a ratio relative to the TPM at the ‘log_2_(TPM)’ peak, with values coloured from white (low) to blue (high). (f–i), Kernel‐density plots of log_2_(TPM) for key cannabinoid‐biosynthesis genes using Hap2, Yushe, Cannbio‐2 and Cs10 as reference assemblies (Hap2 lacks *THCAS* gene). Each gene‐expression panel shows density curves of log_2_(TPM) values for all accessions, with the log_2_(TPM) value of that gene in each accession indicated by a vertical dashed line; curves and markers are colour‐coded by accession. Comparing these values with the peak positions provides an indirect measure of relative expression among accessions.
**Figure S19:** Phylogenetic relationships and repeat element compositions among *Cannabis sativa* genomes and related species. (a) A maximum‐likelihood phylogenetic tree was constructed from 16 single‐copy orthologous genes for 15 plant genomes, including eight *C. sativa* assemblies (Hap1, Hap2, Cs10, PK, Cannbio‐2, JL, Finola and Yushe) and seven other species (*Parasponia andersonii*, *Morus notabilis*, *Ziziphus jujuba*, *Malus domestica*, *Prunus mume*, *Theobroma cacao* and *Papaver somniferum*), with *Papaver somniferum* used as the outgroup. Within *C. sativa*, Hap1, Hap2 and Cs10 represent cannabidiol (CBDA)‐producing cultivars and form a tight cluster; THCA‐producing cultivars Cannbio‐2 and PK group into a distinct clade; oilseed cultivar Yushe and fibre cultivar Finola cluster closely together, while JL, a wild‐type accession, occupies an independent branch. (b) Stacked bar plots illustrating the relative abundance of major TE categories: Gypsy (orange), Copia (purple), DNA transposons (green) and other repeats (blue), together with non‐transposable element (non‐TE) sequences (red) in the gene‐flanking regions of diverse plant genomes and *Cannabis* accessions.
**Figure S20:** Distribution and conservation of secondary metabolite biosynthetic gene clusters in *Cannabis sativa* genomes. (a) Chromosomal distribution of secondary metabolite biosynthetic gene clusters in the two haplotype‐phased genomes. Coloured blocks denote gene clusters involved in distinct pathways, including alkaloid, lignan, polyketide, saccharide, terpene, and their combinatorial biosynthetic routes. In each chromosome pair, the left and right arms correspond to Hap1 and Hap2, respectively. (b) Synteny of geranyl pyrophosphate (GPP) biosynthetic pathway genes across eight *C. sativa* genomes. Ribbon links indicate conserved orthologs of core pathway genes, including *DXR*, *DXS*, *MCT*, *MCS*, *HDR*, *HDS*, *IDI*, *HMGR*, *HMGS* and *GPPS*, revealing both shared and lineage‐specific retention patterns among cultivars and haplotypes. GCS is shown as two phased haploid assemblies to preserve haplotype‐specific synteny, whereas the other accessions are shown as single collapsed or chimeric assemblies.

## Data Availability

All raw sequencing reads have been submitted to the China National Center for Bioinformation (CNCB; https://ngdc.cncb.ac.cn/) under project number PRJCA041008. The assembled nuclear genome files are publicly accessible at CNCB under the accession numbers GWHGEPJ00000000.1 and GWHGEPK00000000.1. The annotation files are available at Figshare (https://figshare.com/s/46bfd5dd718fbb15153d). All data are provided in the main text or Supporting Information. Additional information is available from the corresponding author upon reasonable request (jianbinlab@caas.cn).
