# Supplementary material for: Gap‐Free Cannabis sativa Assemblies Reveal Post‐Polyploid Genome Remodelling and Refine the Evolution of Cannabinoid Synthase Genes
Source: Plant Biotechnol J. 2026 Oct 22:10.1111/pbi.70766. Online ahead of print. doi: 10.1111/pbi.70766 (PMC13691442; doi:10.1111/pbi.70766)
Supplement: Supplementary file 1 [file PBI-9999-0-s001.pdf]

Supplementary Materials for

Gap-free *Cannabis sativa* assemblies reveal post-polyploid genome remodeling and refine the evolutionary history of cannabinoid synthase genes

Jiangjiang Zhang *et al*.

*Corresponding author. Email: jianbinlab@caas.cn (YJ), lidefang@caas.cn (LD), hzaulilin@mail.hzau.edu.cn (LN).

Part I: Supplementary Methods 4
Estimation of genome size and complexity 4
Assembly quality assessment 4
Epigenomic and Hi-C profiling for putative centromeric inference 4
Genome collinearity and genomic feature analysis 5
Intact LTR phylogenetic and PCA analysis 6
Analysis of biosynthetic gene clusters 6
Part II: Supplementary Results 7
Quality assessment of the genome assemblies 7
The gene-family evolution analysis 7
The whole-genome LTR diversity in *Cannabis sativa* 8
Comparative quality assessment across *Cannabis* genome assemblies 8
Reference choice biases cannabinoid synthase expression analyses 8
Relationships among *C. sativa* accessions and related species with contrasting repeat content 9
Dispersed cannabinoid pathway gene organization across chromosomes 9
Allelic-level selection and centromeric TE enrichment (Tekay/Retand) 9
Minimal ASE and conserved cannabinoid pathway between haplotypes 10
Part III: Supplemental Figures 10

Supplementary FIGURE 1 | Assembly and genomic landscape of two haplotype-phased *Cannabis sativa* genomes. 11
Supplementary FIGURE 2 | Functional annotation and evolutionary analysis of gene families in the haplotype-phased *Cannabis sativa* genomes. 12
Supplementary FIGURE 3 | Centromere identification across the Haplotype 1 genome based on integrated genomic and epigenomic features. 14
Supplementary FIGURE 4 | Centromere identification across the Haplotype 2 genome based on integrated genomic and epigenomic features 16
Supplementary FIGURE 5 | Multi-feature genomic landscape and centromere identification across chromosomes of *Cannabis sativa* Haplotype 1. 17
Supplementary FIGURE 6 | Multi-feature genomic landscape and centromere identification across chromosomes of *Cannabis sativa* Haplotype 2. 18
Supplementary Figure 7 | Macrosyntenic correspondence between *Cannabis sativa* and *Vitis vinifera*. 19
Supplementary FIGURE 8 | Macrosynteny between *Cannabis sativa* and *Buxus austro-yunnanensis*. 20
Supplementary FIGURE 9 | Synteny analysis and whole-genome duplication (WGD) signals in *Cannabis sativa*. 21
Supplementary FIGURE 10 | Comparative divergence patterns among duplicate-gene classes and major LTR superfamilies. 23
Supplementary FIGURE 11 | Genomic distribution, classification, and divergence profiles of LTR retrotransposons across eight *Cannabis sativa* genomes. 24
Supplementary FIGURE 12 | Phylogenetic analysis of intact LTR retrotransposons across *Cannabis sativa* genomes. 26
Supplementary FIGURE 13 | Principal component analysis of intact LTR-RT elements across multiple *Cannabis sativa* genomes. 27
Supplementary FIGURE 14 | Comparative genomic characterization of intact LTR retrotransposons across eight *Cannabis* genomes. 28
Supplementary FIGURE 15 | Variation in the number of LTR retrotransposons across *Cannabis sativa* genomes. 29

Supplementary FIGURE 16 | Gene duplication patterns and chromosomal homology across different *Cannabis sativa* genomes. 30
Supplementary FIGURE 17 | Phylogenetic tree of homologues of *CBDAS*, *THCAS* and *CBCAS* across representative plant species. 31
Supplementary FIGURE 18 | Expression profiles of cannabinoid synthase genes and associated pathways across different *Cannabis sativa* genomes. 33
Supplementary FIGURE 19 | Phylogenetic relationships and repeat element compositions among *Cannabis sativa* genomes and related species. 34
Supplementary FIGURE 20 | Distribution and conservation of secondary metabolite biosynthetic gene clusters in *Cannabis sativa* genomes. 35
Part IV: Tables 36
References 38

Part I: Supplementary Methods

Estimation of genome size and complexity

To estimate the genome size, heterozygosity, and repetitive content of the high-CBD *Cannabis* cultivar GCS, a genome survey analysis was performed using HiFi reads. Traditional genome size estimation based on short-read next-generation sequencing data can be unreliable for the GCS cultivar due to its high repeat content and high heterozygosity resulting from extensive outcrossing. To overcome this limitation, a k-mer–based approach leveraging HiFi reads was used.

K-mer frequency distributions were generated using Jellyfish v1.1.12 (Marçais and Kingsford, 2011) with multiple k-mer sizes, including 17-, 19-, 21-, and 23-mers. The resulting frequency histograms were analyzed using GenomeScope2 v2.0.1 (Ranallo-Benavidez *et al.*, 2020) and findGSE v3.3.1 (Sun *et al.*, 2018) to provide robust and reference-free estimates of genome size and genome-wide complexity.

Assembly quality assessment

The completeness, accuracy, and contiguity of the genome assembly were evaluated using a combination of metrics, including contig N50, scaffold N50, number of gaps, and LAI. BUSCO v6.0.0 (Simão *et al.*, 2015) analysis was performed using the core eukaryotic gene dataset (eukaryota_odb10) to assess gene and annotation completeness. The LAI (Ou *et al.*, 2018) was calculated to quantify the completeness and abundance of intact LTR-RTs, a key indicator of assembly quality in plant genomes.

Standard assembly metrics such as contig N50 and total assembly size were computed using the assembly-stats toolkit (https://github.com/MikeTrizna/assembly_stats). In addition, Merqury v1.3 (Rhie *et al.*, 2020) was employed for k-mer-based, reference-free evaluation of assembly quality, completeness, consensus accuracy, and phasing resolution using a k-mer size of 21.

To assess the accuracy and completeness of the assembled genomes, both long reads (PacBio HiFi and CLR) and short reads (generated by the BGI platform) were aligned to the assemblies using minimap2 v2.30 (Li, 2021) and BWA-MEM v0.7.19 (Li and Durbin, 2010), respectively. Mapping statistics, including overall alignment rate, effective mapping rate, and genome coverage, were calculated using samtools v1.22 (Danecek *et al.*, 2021).

Assembly continuity and basic metrics, including contig N50, total genome size, and GC content, were evaluated with QUAST v5.3.0 (Gurevich *et al.*, 2013). Gene-space completeness was examined using BUSCO v6.0.0 (Simão *et al.*, 2015) against the embryophyta_odb10 dataset under both "--genome" and "--protein" modes.

Epigenomic and Hi-C profiling for putative centromeric inference

ChIP-seq was performed to profile the H3K4me3 modification in *Cannabis sativa*. Fresh young leaves were collected and fixed in 1% formaldehyde under vacuum for 10 min to crosslink protein–DNA interactions, followed by quenching with 0.125 M glycine. Nuclei were isolated and chromatin was sheared to an average

size of 200–500 bp using sonication. Immunoprecipitation was carried out using an anti-H3K4me3 antibody according to the manufacturer's protocol. DNA was purified and libraries were prepared and sequenced on an Illumina platform with 150-bp paired-end reads. Clean reads were mapped to the reference genome using Bowtie2 v2.4.2 (Langmead and Salzberg, 2012), and peaks were called using MACS2 v2.2.7.1 (Zhang *et al.*, 2008) with default parameters. Genome-wide enrichment profiles were visualized using IGV v2.16.0 (Robinson *et al.*, 2023) or deepTools v3.5.5 (Ramírez *et al.*, 2014).

Genomic DNA was extracted from young leaves of GCS using a modified CTAB method. DNA quality and quantity were assessed by agarose gel electrophoresis and Qubit fluorometry. For bisulfite conversion, 500 ng of genomic DNA was treated with bisulfite solution. Bisulfite-converted DNA was used to construct libraries with the TruSeq DNA Methylation Kit (Illumina), and paired-end sequencing (150 bp) was performed on an Illumina NovaSeq 6000 platform.

Raw reads were quality-filtered using fastp v1.0.1 (Chen *et al.*, 2018), and clean reads were mapped to the reference genome using Bismark v0.22.3 (Krueger and Andrews, 2011) with default parameters. Duplicated reads were removed, and genome-wide DNA methylation profiles were visualized using IGV v2.16.0 and R v4.3.0 (R Core Team, 2019).

Hi-C sequencing data were quality-filtered using fastp v1.0.1 (Chen *et al.*, 2018), and high-quality clean reads were retained for subsequent analyses. The filtered reads were then aligned to both haplotype assemblies using Sentieon BWA-MEM (Kendig *et al.*, 2019). After removal of PCR duplicates, the resulting BAM files were used for genome-wide quantification of Hi-C read coverage. Bedtools coverage (Quinlan and Hall, 2010) was used to compute Hi-C read counts in 500-kb windows using a 100-kb sliding step, providing a genome-wide overview of chromatin contact density.

Genome collinearity and genomic feature analysis

Syntenic relationships among multiple genomes were analyzed and visualized using NGenomeSyn v1.41 (He *et al.*, 2023). Homology searches were conducted using DIAMOND v0.9.21 in Sensitive mode (Buchfink *et al.*, 2021) to align CDSs and generate pairwise homologous gene sets. The resulting homologous gene pairs and genome annotations (GFF3 and FASTA) were then used as input for NGenomeSyn, with customized parameters (e.g., minimum number of genes per syntenic block and maximum intergenic distance) to define conserved collinear regions and generate synteny plots.

GC content of the genome sequences was calculated using SeqKit v2.10.0 (Shen *et al.*, 2016) through a sliding window analysis with a window size of 500 kb and a step size of 100 kb. This approach enabled genome-wide quantification of GC content and identification of regional variations in nucleotide composition.

Genome-wide visualization of structural features was performed using CIRCOS v0.69-9 (Krzywinski *et al.*, 2009), including chromosome architecture, gene density, TE distribution (specifically Gypsy and Copia elements), GC content, and intergenomic synteny. Gene density and the distribution of Gypsy and Copia

elements were calculated using a sliding window of 500 kb with a step size of 100 kb. Syntenic relationships among genomes were analyzed using MCScanX v1.0.0 (Wang *et al.*, 2024).

In addition, circular plots were independently generated for both haplotype assemblies to display gene density, Gypsy and Copia TE distribution, GC content, synteny, CRM elements, overall TE content, Hi-C contact maps, and LAI, providing an overview of genome architecture and structural variation.

Divergence times among species were estimated using a Bayesian relaxed molecular clock approach, implemented in the MCMCtree program within the PAML package (dos Reis and Yang, 2019). This method integrates phylogenetic information from single-copy gene trees with molecular clock models to infer divergence times. Fossil calibration points were selected based on the TimeTree database (TimeTree.org), and statistical tests were conducted to evaluate the robustness of the time estimates.

Intact LTR phylogenetic and PCA analysis

The classified intact LTR-RT sequences were then retrieved using the SeqKit subseq command and aligned with MAFFT v7.526 (Katoh and Standley, 2013) to generate multiple sequence alignments. Phylogenetic reconstruction was performed using IQ-TREE v2.10.0 (Minh *et al.*, 2020), and the resulting trees were visualized with FigTree v1.4.2 (Rambaut, 2018) and the ggtree (Xu *et al.*, 2022) package in R. PCA was further conducted using the adegenet package (Jombart, 2008) to explore the genetic variation and clustering patterns among intact LTR-RTs.

Analysis of biosynthetic gene clusters

We identified biosynthetic gene clusters (BGCs) in each haplotype-phased genome using plantiSMASH v2.0.1(Kautsar *et al.*, 2017) with default settings. For each haplotype, we provided the chromosome-level assembly (FASTA) and the corresponding gene annotation (GFF3) as input and obtained the genomic coordinates and gene content of all predicted BGCs. We then compared BGCs between the two haplotypes by intersecting cluster intervals and orthologous gene sets and classified clusters as conserved when they shared most of their genes and genomic span, or as haplotype-specific when they lacked a corresponding cluster in the other haplotype. The resulting BGC sets were used to summarize and compare the repertoire and organization of secondary metabolic gene clusters between haplotypes.

Part II: Supplementary Results

Quality assessment of the genome assemblies

HiFi and CLR reads mapped at 99.99% to both haplotype assemblies, whereas BGI short reads mapped at 99.30% and 99.08% for Hap1 and Hap2, respectively (Tables S11 to S13). HiFi coverage exceeded 50× on all chromosomes, with maxima on Chr8 (81.20× in Hap1; 85.07× in Hap2). Summaries in non-overlapping 50-kb windows showed per-chromosome median depths close to the genome-wide median (RelToGenome ≈ 1×; Table S14). CLR alignments showed broad coverage, with six chromosomes in Hap1 and seven in Hap2 reaching 100% breadth and depths of ~100–120×, peaking on Chr8 (168.90× in Hap1; 196.24× in Hap2). BGI coverage ranged from 68 to 143×, again highest on Chr8 (133.20× in Hap1; 143.02× in Hap2). Together, the near-complete mapping rates and consistently high per-chromosome depths from three independent sequencing platforms provide strong support for the accuracy and completeness of our chromosome-level assemblies.

Base-level error rates across individual chromosomes ranged from 3.6×10^{-7} (QV 64.5 on ChrX) to 3.1×10^{-6} (QV 55.1 on Chr8) in Hap1 and from 4.7×10^{-7} (QV 63.3 on Chr5) to 3.2×10^{-6} (QV 54.9 on Chr7) in Hap2. Hap2 consistently shows lower error rates and higher QVs on every homology chromosome (Table S15). Weighting error counts by chromosome length yielded genome-wide error rates of 1.47×10^{-6} for Hap1 and 1.16×10^{-6} for Hap2, corresponding to QVs of 58.33 and 59.36, respectively. The two assemblies produced an overall error rate of 1.31×10^{-6} (QV 58.82) (Table S16), representing a ~17% reduction in base errors for Hap2 relative to Hap1. Consistent with these metrics, k-mer analysis showed that the two assemblies individually recovered 86.38% (Hap1) and 86.43% (Hap2) of HiFi-derived k-mers, while together they captured 97.55%, underscoring the high completeness of each haplotype and their strong complementarity.

The gene-family evolution analysis

We performed gene-family expansion and contraction analyses across the sampled species. Overall, gene-family turnover varied substantially among lineages, indicating heterogeneous evolutionary trajectories across the phylogeny. Among all sampled taxa, *Papaver somniferum* showed the most pronounced expansion signal, with 9,195 expanded and 451 contracted gene families. Strong lineage-specific changes were also observed in several other species, including extensive expansion in *Malus domestica* (6,935 expanded and 803 contracted gene families), whereas marked contraction was observed in *Theobroma cacao* (557 expanded and 5,248 contracted gene families). These results indicate that gene-family evolution differed markedly among lineages.

In comparison, *Cannabis sativa* exhibited a relatively limited pattern of gene-family expansion and contraction, with 570 expanded and 2,585 contracted gene families. Within the *Cannabis*-*Humulus* clade, the ancestral branch showed limited change overall, with 331 expanded and 358 contracted gene families. However, the two terminal lineages displayed distinct patterns of gene-family evolution. In particular, *Humulus japonicus*

showed substantially stronger expansion, with 3,343 expanded and 453 contracted gene families, whereas C. sativa was characterized by comparatively fewer expansions and more contractions. Analyses of rapidly evolving gene families further supported this contrast: in C. sativa, 36 gene families were significantly expanded and 255 were significantly contracted, whereas in *H. japonicus*, 540 gene families were significantly expanded and 17 were significantly contracted. These results suggest that, although *Cannabis* and *Humulus* are sister taxa, they followed distinct trajectories of gene-family evolution after their divergence.

The whole-genome LTR diversity in *Cannabis sativa*

Hap1, Hap2, Yushe, EH23, Sri Lankan, Pink, and HOG achieved gold-standard LAI values, whereas Cs10 had the lowest LAI (12.91%) with only 67.55 Mb of intact LTR-RT sequence. Across all assemblies, the total length of intact LTR-RTs was greater for Gypsy than for Copia elements, whereas, low-LAI genomes (Cs10, Cannbio-2 and Finola) were dominated by intact elements classified as unknown. For intact LTR-RTs, Gypsy exceeded Copia in both total length and copy number in the majority of genomes, except Cs10 (Figure S12, A and B). Chromosome-level LAI profiles highlighted variation among assemblies: Hap1 and Hap2 showed uniformly high values with expected reductions near centromeres and telomeres, whereas Cs10 and PK were consistently the lowest, and the remaining genomes showed intermediate, irregular patterns (Figure S12c; Tables S17, S25).

Within the Gypsy superfamily, Tekay was consistently dominant across genomes except in Cs10, underscoring its dominant contribution to LTR-RT content in these assemblies. Retand was the second most abundant Gypsy subfamily in all genomes except Cs10. Among Copia elements, Sire and Ale were the most abundant, whereas in Cs10, Ale and Ikeros were predominant. Raw LTR element copy-number analyses showed that unknown elements were the most abundant class, with Copia second and Gypsy the lowest, except in Finola, where Gypsy outnumbered Copia (Figure S13a, Table S26).

Comparative quality assessment across *Cannabis* genome assemblies

Both haplotype assemblies are gap-free, with contig N50 values of approximately 80 Mb, dramatically exceeding the second-ranking Pink genome, which contained 61 gaps and has a contig N50 of just 23.50 Mb. Furthermore, GCS and EH23 possess the highest LAI values (23.54 and 23.24, 24.38 and 22.31), followed by Pink (21.61), Hawthorne (22.89), Sri Lankan (21.58), and HOG (23.07), which show comparable assembly quality. Other genomes have LAI scores ranging from 10.97 to 19.64 (Table S17). Despite having a contig N50 of 1.96 Mb, the Cs10 genome had the second-lowest LAI (14.07) and showed lower BUSCO completeness than several assemblies with shorter contig N50, indicating that high contiguity alone does not guarantee structural and functional completeness.

Reference choice biases cannabinoid synthase expression analyses

Reference choice biases expression analyses: switching among GCS, Cs10, Yushe, and Cannbio-2 as reference genomes led to strikingly different apparent expression profiles for *CBDAS*, *THCAS,* and *CBCAS* across

accessions, largely reflecting the presence, absence, or incomplete assembly of these genes in each reference (Figure S16; Tables S31-S36). For example, Cannbio-2 can produce both THCA and CBDA yet lacks an intact annotated *CBDAS*, and two highly expressed *CBDAS*-like genes flanking a sequence gap most likely represent a single *CBDAS* gene fragmented by assembly error (Figure S16d). Moreover, the high sequence similarity among *THCAS*, *CBCAS,* and *CBDAS* regions on chromosome 7 favours read misalignment and cross-mapping, further distorting expression estimates. Together, these observations indicate that cannabinoid pathway gene expression is highly sensitive to reference choice and that robust inferences require structurally continuous assemblies with complete, correctly assembled copies of the target synthase and pathway genes.

Relationships among *C. sativa* accessions and related species with contrasting repeat content

Phylogenetic analysis based on eight representative *C. sativa* accessions and related species recovered relationships broadly consistent with previous classifications, while also resolving chemotype- and use-related structure: GCS (Hap1, Hap2) and Cs10 clustered as high-CBDA varieties, Yushe and Finola grouped as seed- and hemp-type accessions, PK and Cannbio-2 formed a high-THC clade, and JL occupied an isolated position, consistent with its distinct genetic background (Figure S19a). Across the same panel, *Cannabis* genomes showed consistently higher repeat content than non-*Cannabis* relatives, with substantial variation among accessions and elevated levels also observed in the closest relative *Parasponia andersonii* within Cannabaceae (Figure S19B, Table S37). These patterns indicate that both chemotype diversification and repeat proliferation have contributed to the genomic differentiation of *C. sativa* within Cannabaceae.

Dispersed cannabinoid pathway gene organization across chromosomes

We analyzed the biosynthetic gene clusters of secondary metabolites in the two haplotype-phased genomes. Comparison of the two haplotype-phased assemblies revealed both conserved and divergent features, highlighting intraspecific genomic diversity (Figure S20a; Table S36). The genes involved in cannabinoid biosynthesis were found to be distributed across all chromosomes (Figure 7b, Figure S20b). Notably, Chr4 (*MCT*, *DXS*, *GPPS*), Chr6 (*DXS*, *GPPS*), Chr9 (*DXS*, *GPPS*, *OAC*), and ChrX (*HMGR*, *HDR*, *aPT4*) contained multiple biosynthetic genes, whereas the other chromosomes each carried only one pathway gene. These findings suggest that cannabinoid biosynthesis genes are not confined to a single genomic cluster, but instead show a conserved yet dispersed genomic distribution.

Allelic-level selection and centromeric TE enrichment (Tekay/Retand)

We identified 155 genes under positive selection (Table S29). These are enriched almost exclusively for endonuclease activity and do not overlap with rapidly expanding or contracting gene families, indicating that they are subject to evolutionary pressures distinct from those driving gene family size changes. Among the positively selected genes, 98.1% were shared alleles present in both haplotypes, while only three were haplotype-specific: *Cannabis1G15546* in Hap1 and *Cannabis2G03488* and *Cannabis2G11611* in Hap2. This

pattern suggests that adaptive evolution in *C. sativa* predominantly acts at the allelic level, with haplotype-specific genes potentially regulating unique phenotypes or functions. Additionally, these haplotype-specific genes showed little association with rapid expansion or contraction. Putative centromeric regions in *C. sativa* were enriched in Tekay and Retand elements, which are among the most active, and this enrichment pattern is consistent with findings from a pangenome study (Lynch *et al.*, 2025). These regions exhibited complex variation patterns across chromosomes, characterized by distinct transposon compositions and marker distributions.

Minimal ASE and conserved cannabinoid pathway between haplotypes

We also analyzed allelic gene expression within the two haplotype genomes of GCS. Among 17,838 strict one-to-one allelic gene pairs, only 625 genes showed significant allele-specific expression (ASE), accounting for less than 4% of the total. Gene Ontology (GO) enrichment analysis of these ASE genes showed significant associations with chromatin structure, chromosome organization, DNA replication, and cell cycle control. This suggests that allelic divergence is enriched in genes involved in genome structural dynamics. The limited number of ASE genes suggests high sequence similarity and regulatory conservation between the two haplotypes. Notably, the cannabinoid biosynthetic genes *AAE1*, *OAC*, *CBCAS*, and *CBDAS* (but not *OLS* or *aPT4*) were identified as strict one-to-one allelic pairs across the two phased haplotypes. Their expression levels were highly consistent between haplotypes, with no evidence of allele-specific expression. These findings suggest that the cannabinoid biosynthetic pathway is both structurally and transcriptionally conserved between the haplotypes of GCS, reflecting its evolutionary stability and potential dosage sensitivity.

Part III: Supplemental Figures

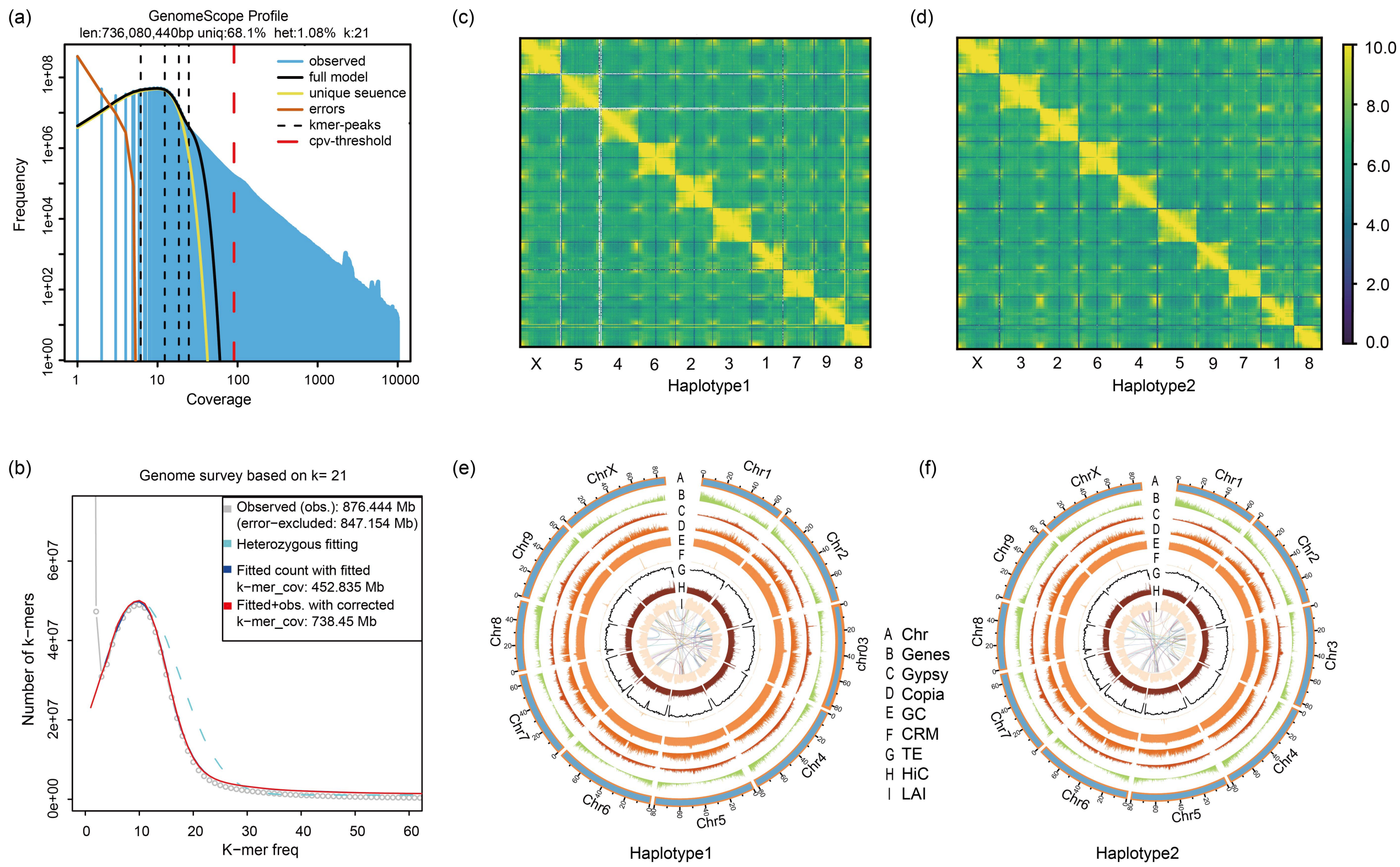

Supplementary FIGURE 1 | Assembly and genomic landscape of two haplotype-phased *Cannabis sativa* genomes. (a) GenomeScope k-mer distribution of 21-mers for *C. sativa*. The x-axis represents k-mer coverage (log scale), and the y-axis indicates the k-mer frequency (log scale). The observed k-mer spectrum (blue) is fitted by the full model (black), with separate curves representing unique (yellow) and erroneous (orange) k-mers. Vertical dashed lines mark key model parameters, including the heterozygous peak, and homozygous peak, low-copy repeat peak, and high-copy repeat peak, respectively; the red dashed line marks the model-defined coverage threshold for filtering high-frequency or aberrant k-mers. The model estimates a genome size of 738.08 Mb with a heterozygosity of 1.08% and a sequencing error of 0.26%. (b) K-mer frequency distribution of 21-mers analyzed using findGSE. The observed k-mer distribution (gray) was fitted using a genome size estimation model (red), with a secondary curve (blue) representing an intermediate fitting step. The dashed cyan line indicates the theoretical peak for diploid homozygous regions. The model estimated a genome size of 738.45 Mb with a corrected k-mer coverage of 23.17×, a heterozygosity rate of 6.4%, and a repetitive content of 387.84 Mb (53% of the genome). A fitted genome size based solely on filtered k-mers yielded 452.84 Mb at 34.94× k-mer coverage. (c, d) Final Hi-C contact heatmaps of *Cannabis sativa* assemblies for Haplotype 1 (c) and Haplotype 2 (d). Chromosome identifiers are shown along both axes, corresponding to scaffolded pseudochromosomes. Interaction intensity is color-coded on a log scale from yellow (strong) to purple (weak), with prominent diagonal blocks indicating consistent intra-chromosomal contacts and supporting the structural accuracy of the final assemblies. (e, f) Circular representation of genomic and epigenomic features for *Cannabis sativa* Haplotype 1 (e) and Haplotype 2 (f). Tracks from outer to inner represent: I, chromosomes; II, gene density; III, Gypsy LTR retrotransposons; IV, Copia LTR retrotransposons; V, GC content; VI, CRM elements; VII, overall TE density; VIII, Hi-C contact intensity; IX, LTR Assembly Index (LAI). All features are calculated in sliding windows along each chromosome.

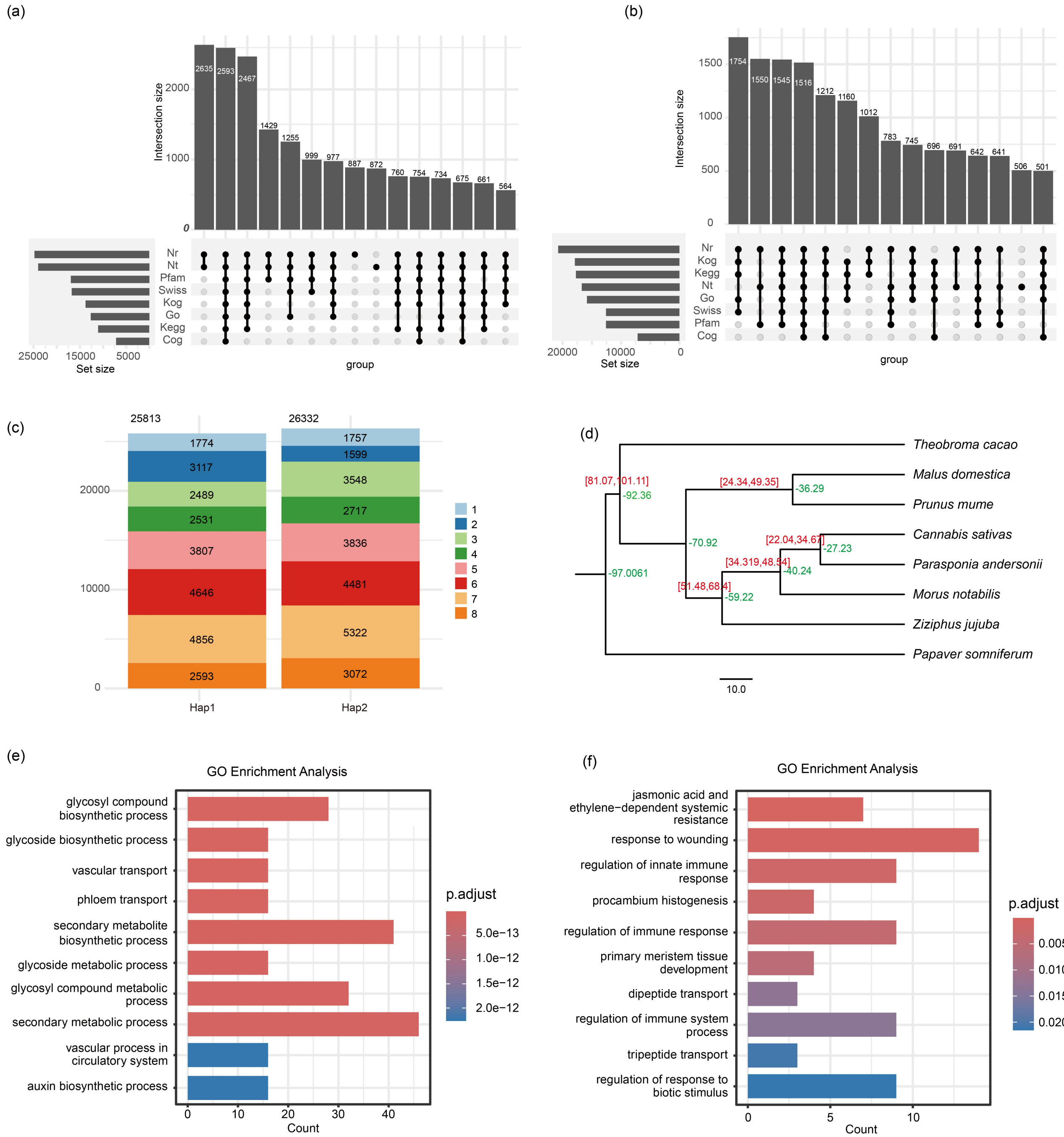

Supplementary FIGURE 2 | Functional annotation and evolutionary analysis of gene families in the haplotype-phased *Cannabis sativa* genomes. (a, b) UpSet plots show the distribution of genes functionally annotated in eight databases for Haplotype 1 (a) and Haplotype 2 (b), respectively. Databases include NR, KOG, KEGG, NT, GO, Swiss-Prot, Pfam, and COG. The upper bar plot shows the number of genes annotated in each specific combination of databases, with the corresponding database combinations in the matrix below. (c) Summary of gene annotation across multiple databases for Haplotype 1 and Haplotype 2. Stacked bar plots depict the number of genes annotated by one to eight functional databases in Haplotype 1 (left) and Haplotype 2 (right). Each colour represents the number of databases supporting a given gene annotation, from dark blue (one database) to orange (eight databases). Segment heights are scaled to the number of genes, and the totals are labelled above the bars. (d) Phylogenetic tree inferred from single-copy orthologs among *C. sativa* and seven other species. Red numbers denote the divergence-time range for each node, while green numbers

represent the mean divergence time (MYA). The scale bar (10.0) represents 10 million years. (e, f) Gene Ontology (GO) enrichment analysis of significantly expanded (e) and contracted (f) gene families in *C. sativa*.

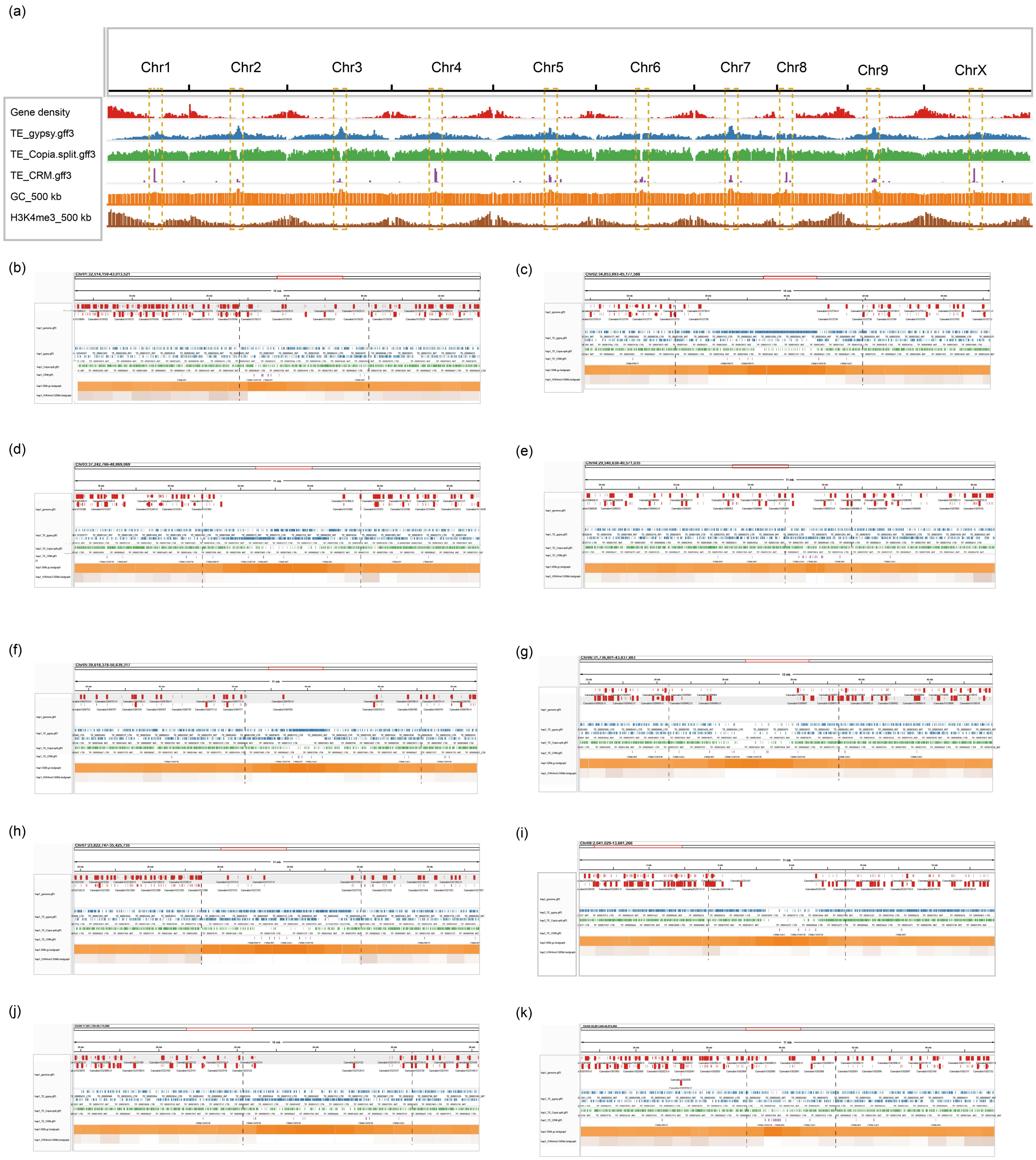

Supplementary FIGURE 3 | Centromere identification across the Haplotype 1 genome based on integrated genomic and epigenomic features. (a) Genomic landscape of ten *Cannabis sativa* chromosomes showing gene density, Gypsy and Copia transposable element density, CRM element distribution, GC content, and H3K4me3 ChIP-seq signal. Gene density and TE content were calculated in non-overlapping 500-kb windows. H3K4me3 enrichment and CRM elements were shown as genome tracks. Brown dashed lines indicate the putative centromeric region, inferred by comparative analysis of gene density, GC content, and the distribution of Gypsy, Copia, and CRM elements. (b–k), Detailed views of centromeric regions on each chromosome (Chr01 to Chr0X), marked by black dashed lines. Centromeres were identified by local enrichment of CRM and Gypsy elements, reduced gene density, increased GC content, and depletion of

H3K4me3 signal. These features collectively support the structural and epigenetic delineation of centromere positions across the *C. sativa* genome.

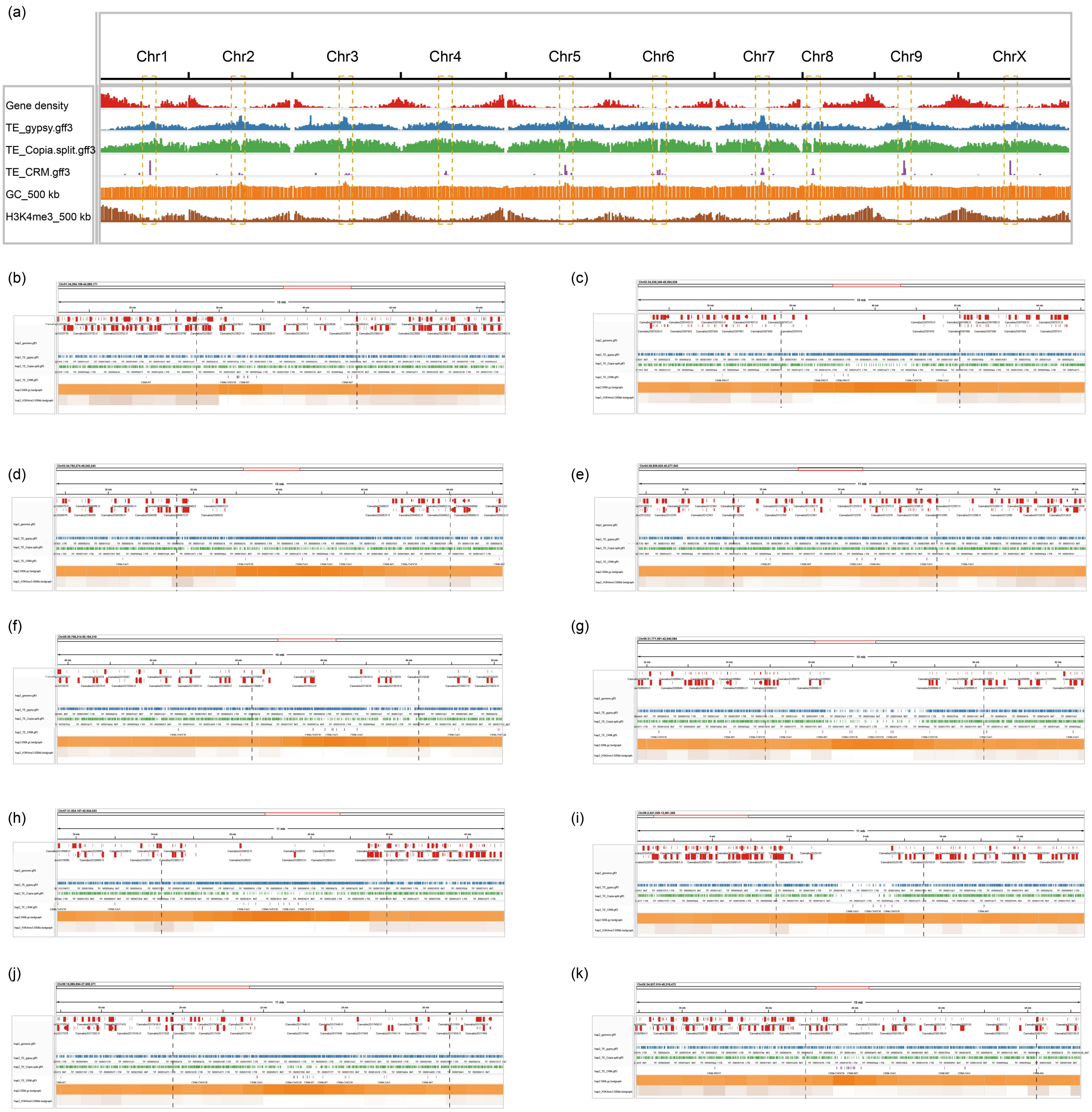

Supplementary FIGURE 4 | Centromere identification across the Haplotype 2 genome based on integrated genomic and epigenomic features. (a) Genomic landscape of ten *Cannabis sativa* chromosomes showing gene density, Gypsy and Copia transposable element density, CRM element distribution, GC content, and H3K4me3 ChIP-seq signal. Gene density and TE content were calculated in non-overlapping 500-kb windows. H3K4me3 enrichment and CRM elements were shown as genome tracks. Brown dashed lines indicate the putative centromeric region, inferred by comparative analysis of gene density, GC content, and the distribution of Gypsy, Copia, and CRM elements. (b–k) Detailed views of centromeric regions on each chromosome (Chr01 to Chr0X), marked by black dashed lines. Centromeres were identified by local enrichment of CRM and Gypsy elements, reduced gene density, increased GC content, and depletion of H3K4me3 signal. These features collectively support the structural and epigenetic delineation of centromere positions across the Haplotype 2 genome of *C. sativa*.

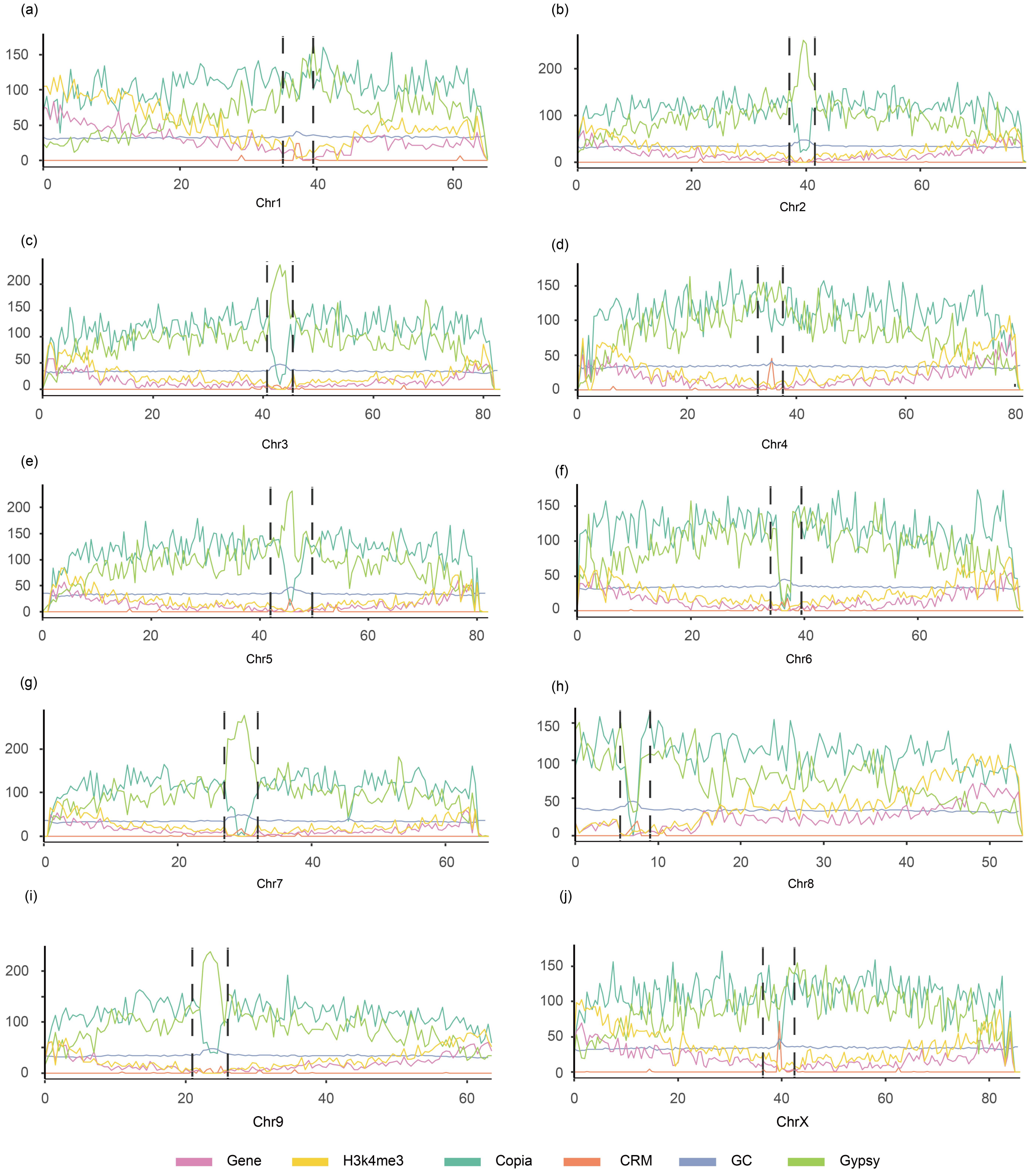

Supplementary FIGURE 5 | Multi-feature genomic landscape and centromere identification across chromosomes of *Cannabis sativa* Haplotype 1. (a–j) Line plots showing the distribution of genomic and epigenomic features along each chromosome of *C. sativa* Haplotype 1. Features include gene density, H3K4me3 histone modifications, GC content, and transposable elements (Copia, Gypsy, and centromeric retrotransposons, CRM). Putative centromeric regions were inferred based on peaks in CRM density; reductions in gene and H3K4me3 signals, and localized shifts in GC content, and are marked by vertical dashed lines. Chromosomes Chr01 to Chr0X are shown in panels (a)–(j), respectively. All features were calculated in 500-kb sliding windows with a 100-kb step size across chromosomes.

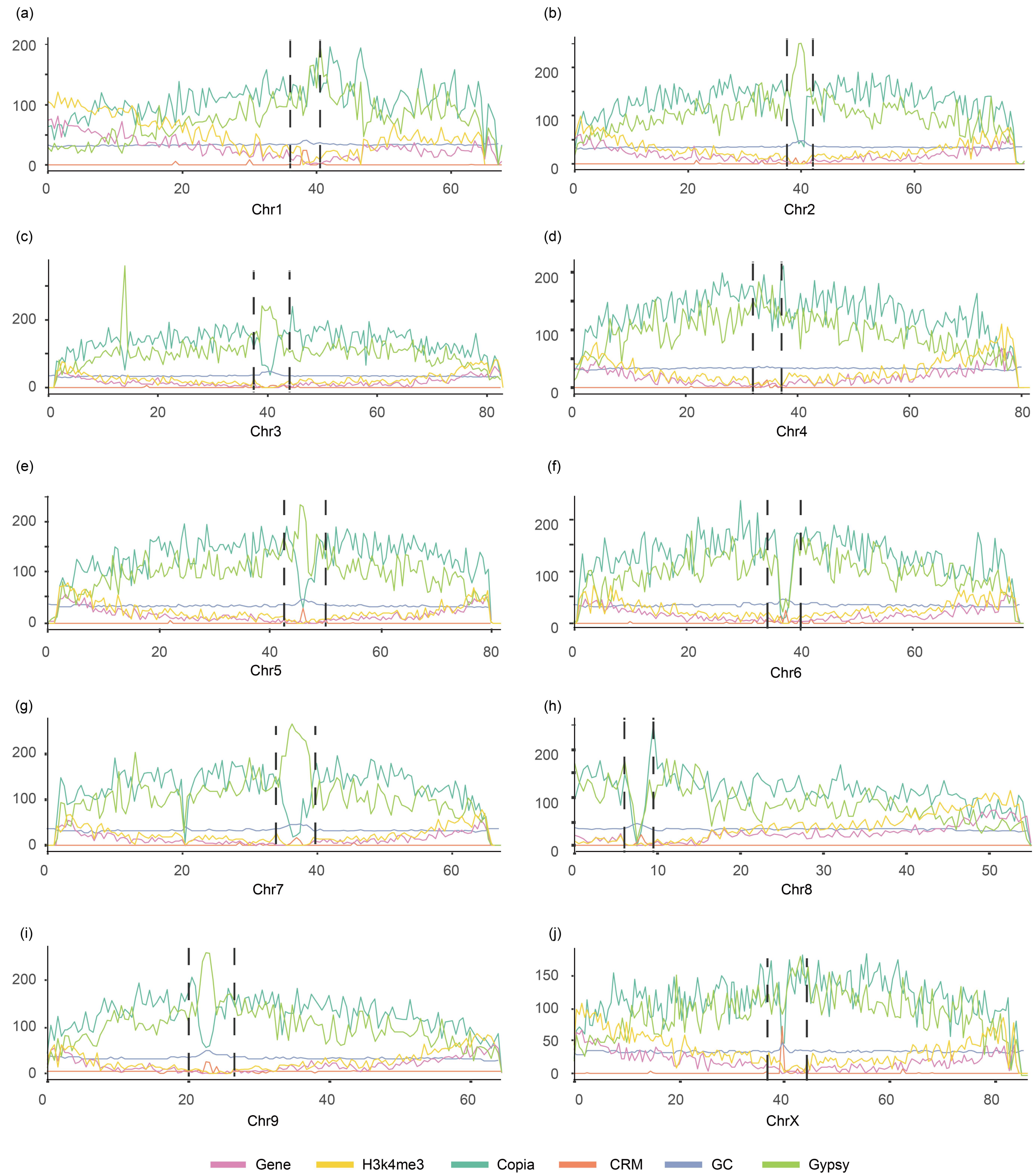

Supplementary FIGURE 6 | Multi-feature genomic landscape and centromere identification across chromosomes of *Cannabis sativa* Haplotype 2. (a–j) Line plots illustrating the distribution of genomic and epigenomic features along each chromosome of *Cannabis sativa* Haplotype 2. Features include gene density, H3K4me3 histone modifications, GC content, and transposable elements (Copia, Gypsy, and centromeric retrotransposons, CRM). Putative centromeric regions were inferred based on peaks in CRM density, reductions in gene and H3K4me3 signals, and local changes in GC content; they are marked by vertical dashed lines. Chromosomes Chr01 to Chr0X are shown in panels (a)–(j), respectively. All features were calculated in 500-kb sliding windows with a 100-kb step size across chromosomes.

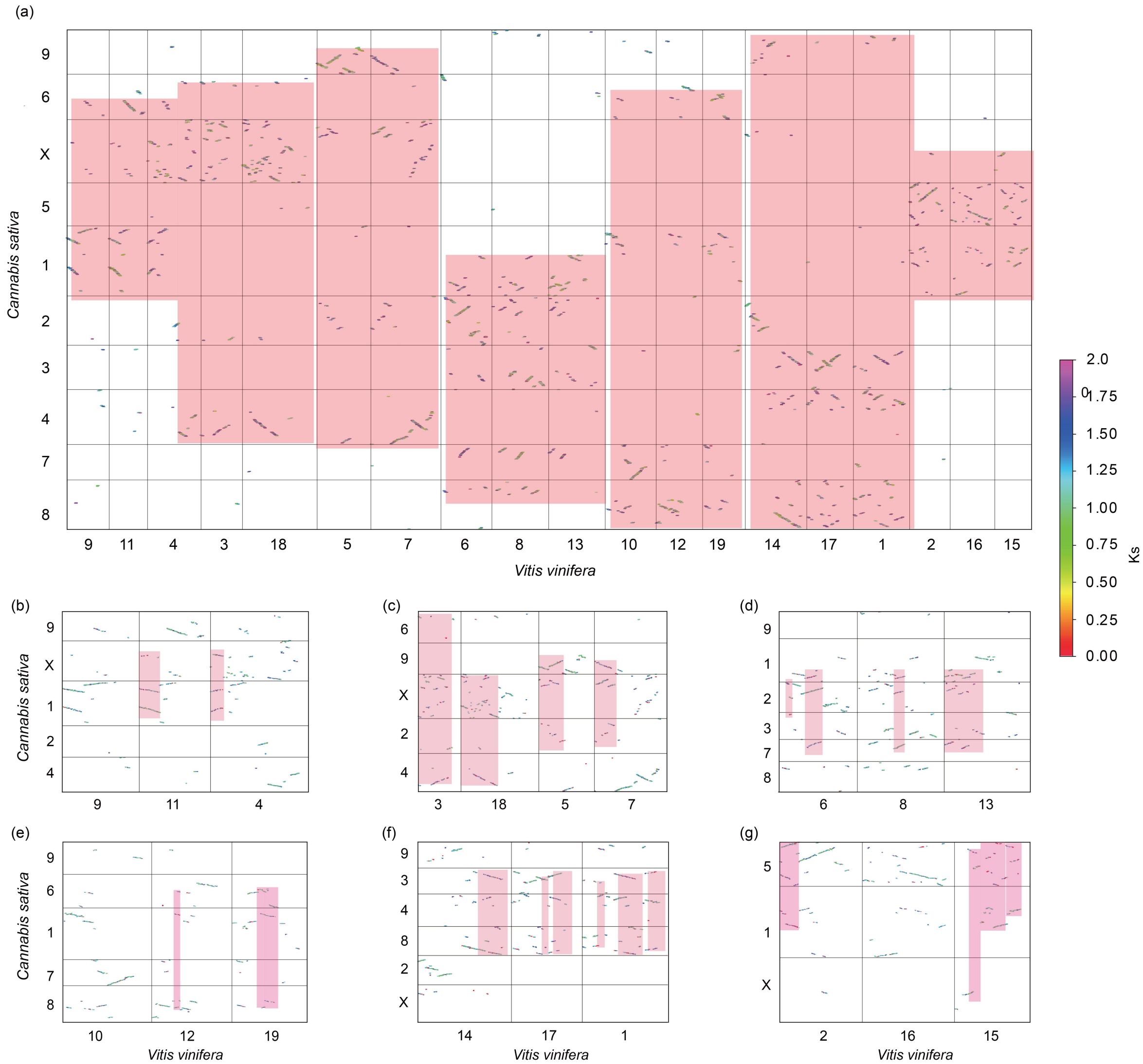

Supplementary Figure 7 | Macrosyntenic correspondence between **Cannabis sativa** and **Vitis vinifera**. (a) Genome-wide macrosyntenic comparison between *C. sativa* and *V. vinifera*. Colored short line segments indicate collinear gene pairs, with colors representing synonymous substitution rates (Ks) of the corresponding anchors. Pink shaded blocks highlight the major chromosome-level syntenic correspondences emphasized for visualization. (b–g) Enlarged views of representative syntenic regions highlighted in panel (a), showing repeated chromosome-scale correspondences between the two genomes. These local and genome-wide patterns are consistent with the retention of ancient polyploid-derived syntenic relationships in *C. sativa*.

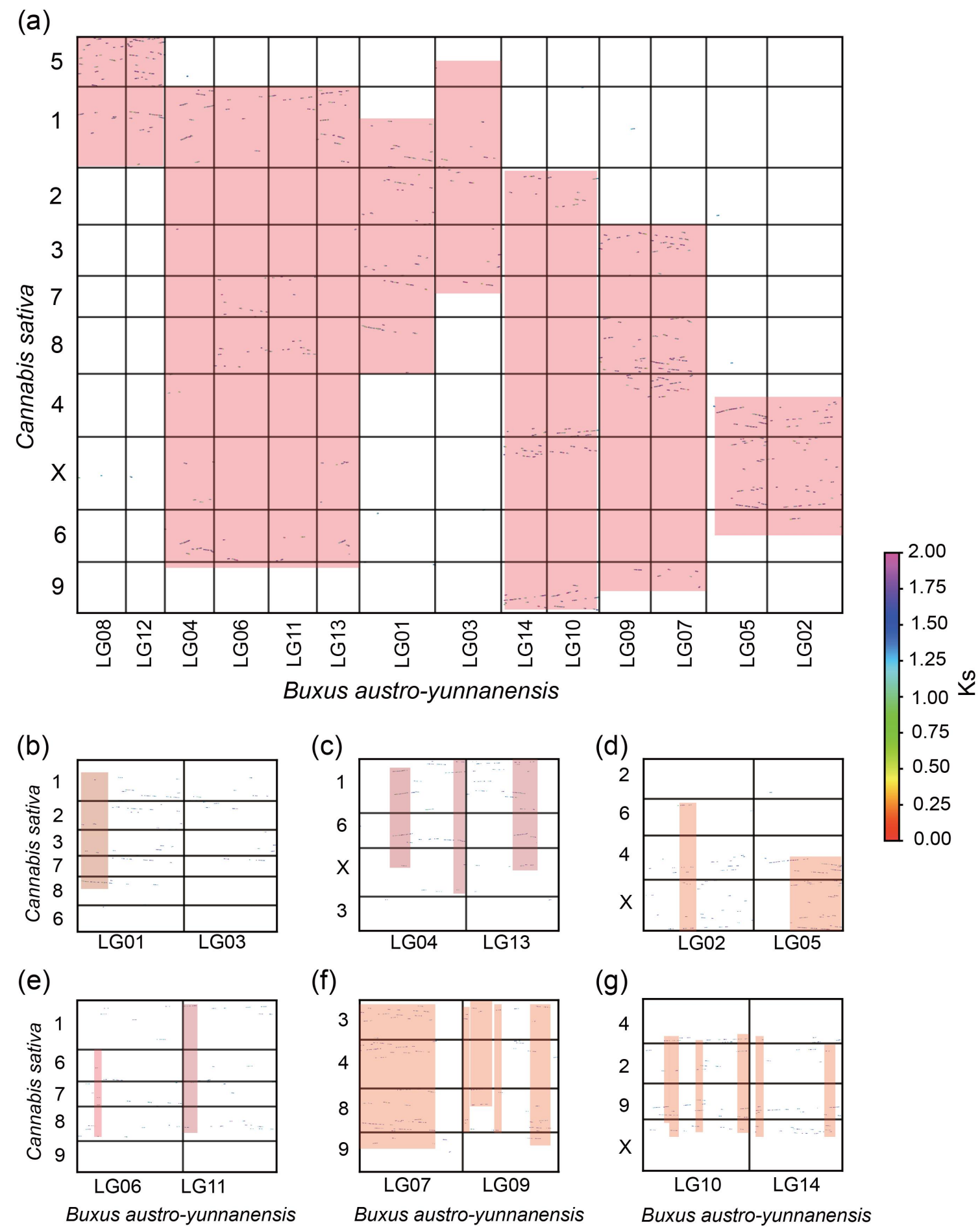

Supplementary FIGURE 8 | Macrosynteny between *Cannabis sativa* and *Buxus austro-yunnanensis*. (a) A Ks-colored macrosynteny heatmap showing homologous relationships between *C. sativa* chromosomes and *B. austro-yunnanensis* linkage groups (LGs). Pink-shaded blocks represent regions of conserved synteny. (b-g**)**, Enlarged views of selected syntenic blocks highlighted in (a), displaying dot plots of homologous gene pairs between specific chromosome–LG pairs. Each dot is colored by synonymous substitution rate (Ks).

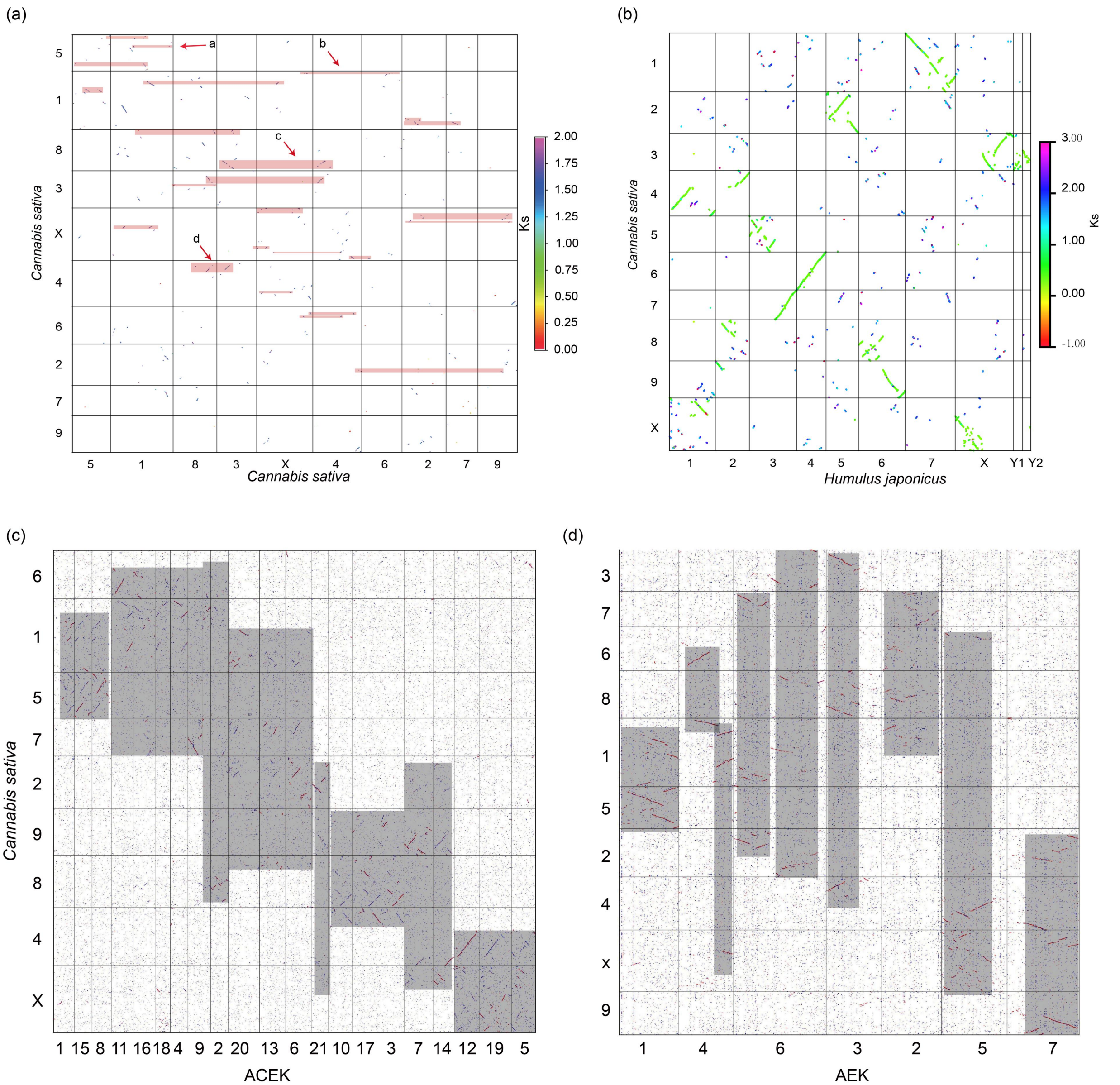

Supplementary FIGURE 9 | Synteny analysis and whole-genome duplication (WGD) signals in *Cannabis sativa*. (a) Dot plot showing self-synteny among the ten *Cannabis sativa* chromosomes. Homologous gene pairs are colored by synonymous substitution rate (Ks), with red indicating recent duplications and purple representing ancient events. Red-shaded blocks indicate high-confidence syntenic regions. Red arrows mark representative duplication events: **a**, intrachromosomal duplications within Chr05 and Chr01; **b**, interchromosomal syntenic blocks between Chr01 and Chr0X, and between Chr01 and Chr06; **c**, collinear blocks between Chr08 and Chr03, as well as between Chr08 and Chr04; **d**, segmental duplications involving Chr04 and Chr08, as well as Chr04 and Chr03. The Ks distribution reveals evidence of both ancient whole-genome duplication remnants and more recent, localized duplication events. (b) Chromosome-scale synteny between *Cannabis sativa* and *Humulus lupulus* colored by synonymous divergence. Each dot represents a syntenic anchor gene pair plotted according to its chromosomal coordinates in C. sativa (vertical axis) and H. lupulus (horizontal axis). Gray grid lines indicate chromosome boundaries. Dot colors show Ks values as indicated by the scale bar, highlighting largely 1:1 collinear relationship between chromosomes of the two species. (c, d) Dot plots illustrating synteny between *Cannabis sativa* (Y-axis) and ancestral genomes reconstructed from Vitis vinifera and Buxus austro-yunnanensis (X-axis), respectively. Each dot represents a homologous gene pair, with darker regions marking duplicated ancestral segments retained in the *Cannabis*

genome. Red indicates high syntenic conservation, while blue denotes lower homology. These plots highlight conserved ancestral genomic blocks, extensive chromosomal rearrangements, and signatures of ancient polyploidy events.

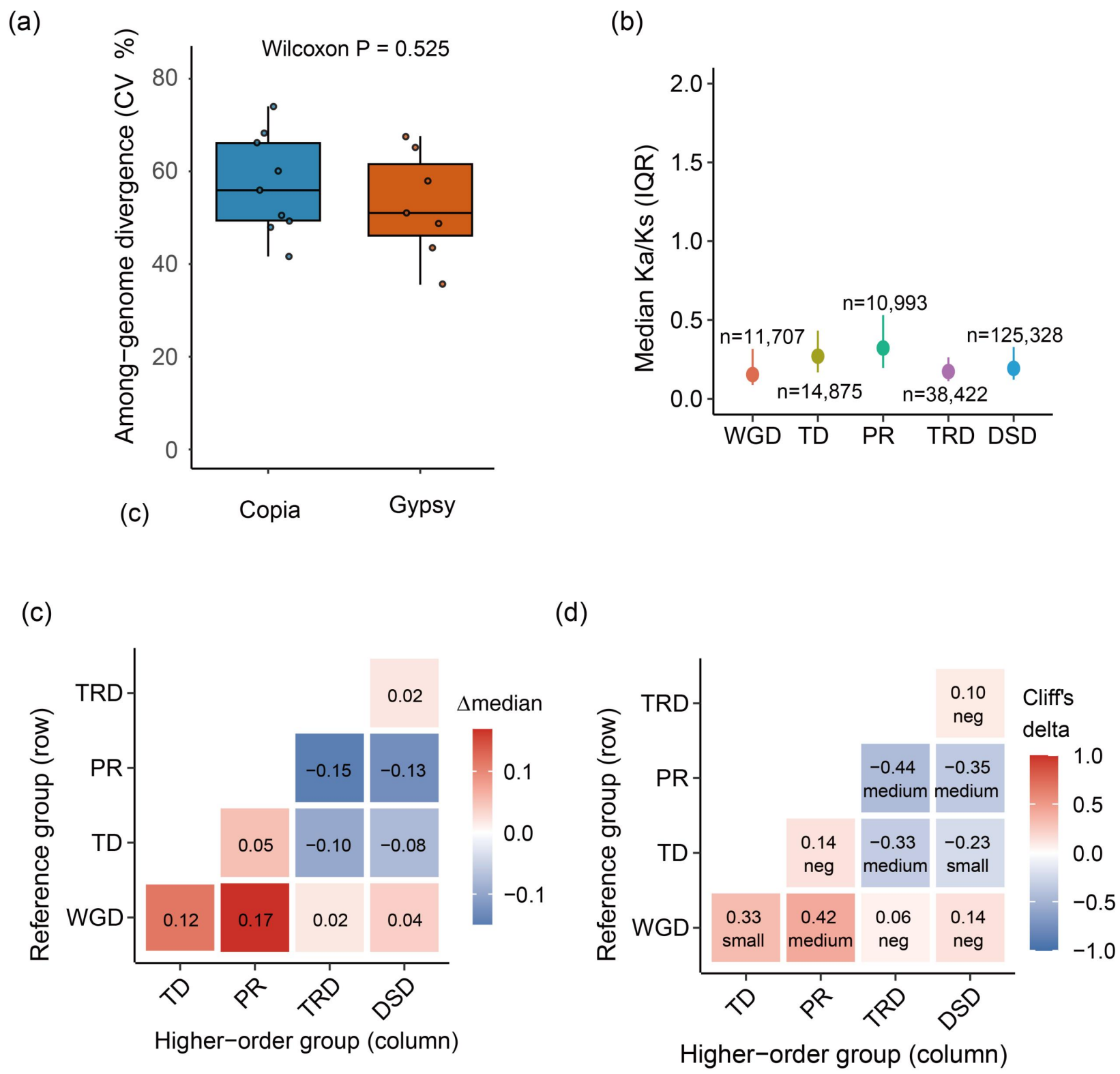

Supplementary FIGURE 10 | Comparative divergence patterns among duplicate-gene classes and major LTR superfamilies. (a) Comparison of among-genome divergence, expressed as coefficient of variation (CV, %), between the Copia and Gypsy superfamilies across *Cannabis* genomes. Each point represents one genome. Boxplots show the median and IQR, and whiskers extend to 1.5 × IQR. The P value was calculated using a two-sided Wilcoxon rank-sum test. (b) Median Ka/Ks values of duplicate gene pairs classified by duplication mode, with vertical bars indicating the interquartile range (IQR). Numbers above each point indicate the number of gene pairs in each category. WGD, whole-genome-duplication-derived duplicates; TD, tandem duplicates; PR, proximal duplicates; TRD, transposed duplicates; DSD, dispersed duplicates. (c) Pairwise differences in median Ka/Ks among duplication classes. Each cell shows Δmedian, calculated as the median Ka/Ks of the column group minus that of the row group. (d) Cliff's delta effect sizes for the same pairwise comparisons shown in panel (b). Positive values indicate relatively higher Ka/Ks values in the column group than in the row group, whereas negative values indicate the opposite. Qualitative effect-size categories are shown within cells.

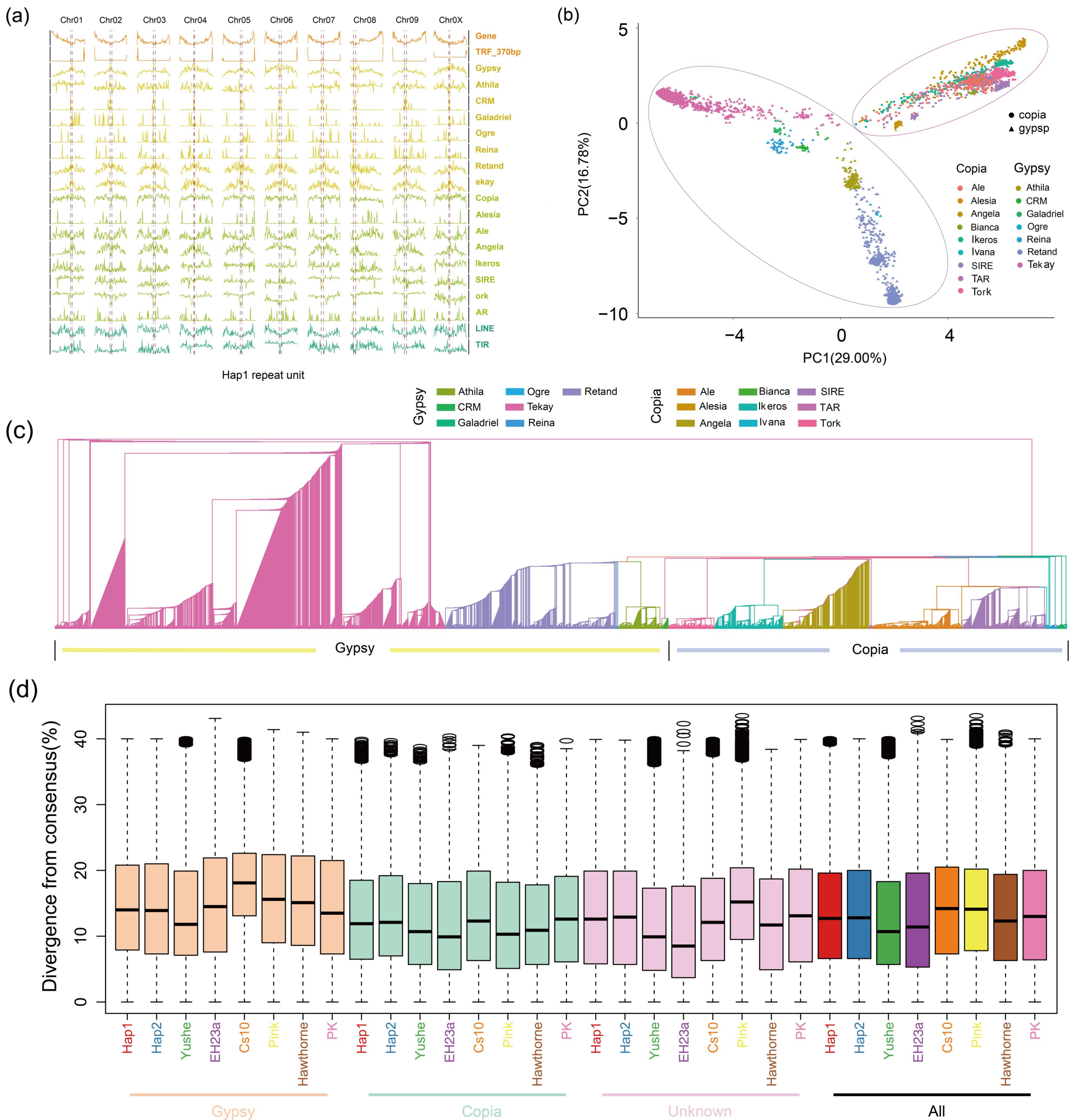

Supplementary FIGURE 11 | Genomic distribution, classification, and divergence profiles of LTR retrotransposons across eight *Cannabis sativa* genomes. (a) Chromosomal distributions of genes and transposable elements (TEs) along Hap1 chromosomes. Gypsy and Copia superfamilies are shown in the upper and middle panels, respectively, and DNA elements are shown in the lower panel. The names of individual TE subfamilies are labeled on the right. Chromosome identifiers are displayed at the top, and dashed vertical lines indicate inferred centromere positions. (b) Principal component analysis (PCA) of intact LTR retrotransposon (LTR-RT) sequences from the Hap1 genome. Subfamilies are color-coded. Gypsy and Copia elements are distinguished by shape (triangles and circles, respectively). Ellipses represent 95% confidence intervals for each superfamily. (c) Maximum-likelihood phylogenetic tree of intact LTR-RTs from the Hap1 genome. Subfamilies are color-coded and annotated along the x-axis. The Gypsy and Copia superfamily regions are indicated below the tree. (d) Boxplots showing sequence divergence from consensus (%) across Gypsy, Copia, and Unknown LTR-RT superfamilies in eight *C. sativa* assemblies. Subfamily classifications are shown in

consistent colors across panels, while in the final group ("All"), divergence values across all LTR-RTs are colored corresponding to individual genomes.

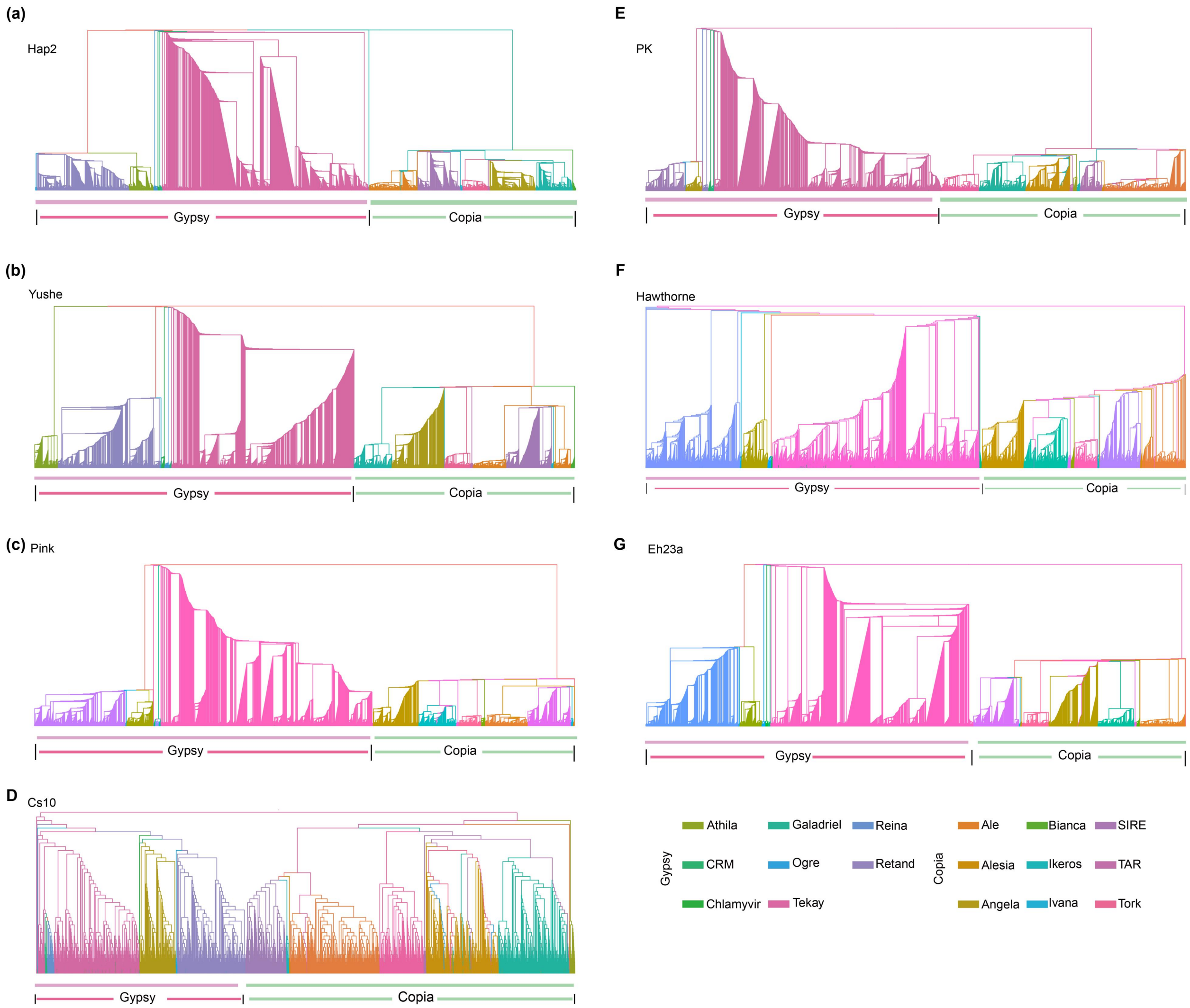

Supplementary FIGURE 12 | Phylogenetic analysis of intact LTR retrotransposons across *Cannabis sativa* genomes. (a-g) Maximum-likelihood phylogenetic trees of intact LTR-RTs (Gypsy and Copia) for seven *C. sativa* assemblies: (a) Hap2, (b) Yushe, (c) Pink, (d) Cs10, (e) PK, (f) Hawthorne, and (g) EH23a. Clades are color-coded by LTR-RT lineage, classified based on conserved domain annotation. The distribution and structure of these clades reflect the diversity and amplification histories of retrotransposons within each genome.

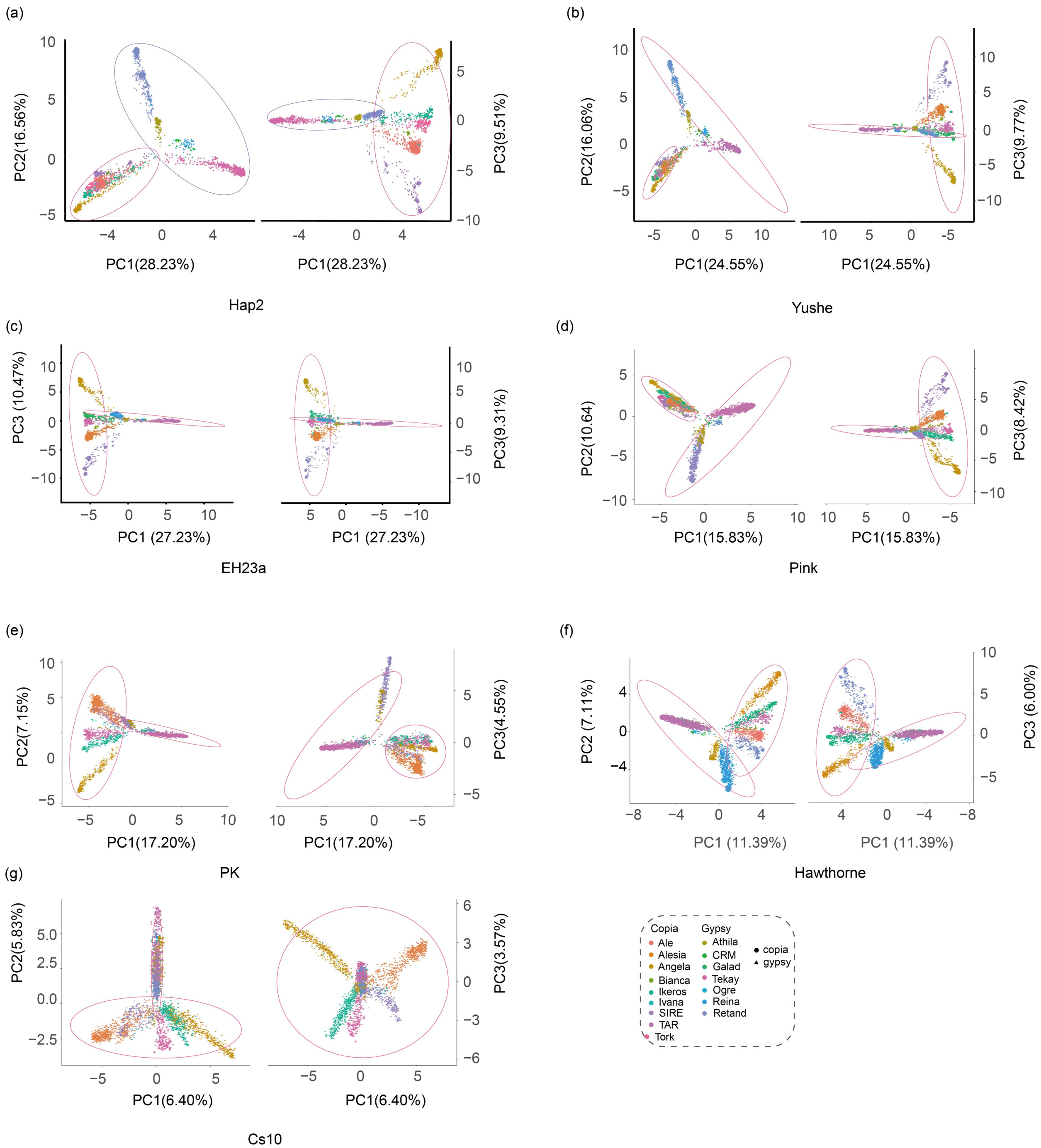

Supplementary FIGURE 13 | Principal component analysis of intact LTR-RT elements across multiple *Cannabis sativa* genomes. (a-g) PCA plots of intact long terminal repeat retrotransposons (LTR-RTs) from seven *Cannabis sativa* assemblies. For each panel genome, the left subplot shows PC1 versus PC2 and the right subplot shows PC1 versus PC3. Data points represent individual intact LTR-RT insertions, colored by transposable element subfamily as indicated in the legend. Ellipses denote clusters of related elements. The paired presentation of PC1-PC2 and PC1-PC3 highlights genome-specific differences in intact LTR-RT differentiation and shows that the convergence patterns of Gypsy and Copia elements vary among genomes. Genome names are indicated next to each pair of plots, and colors correspond to LTR-RT subfamilies as shown in the common legend.

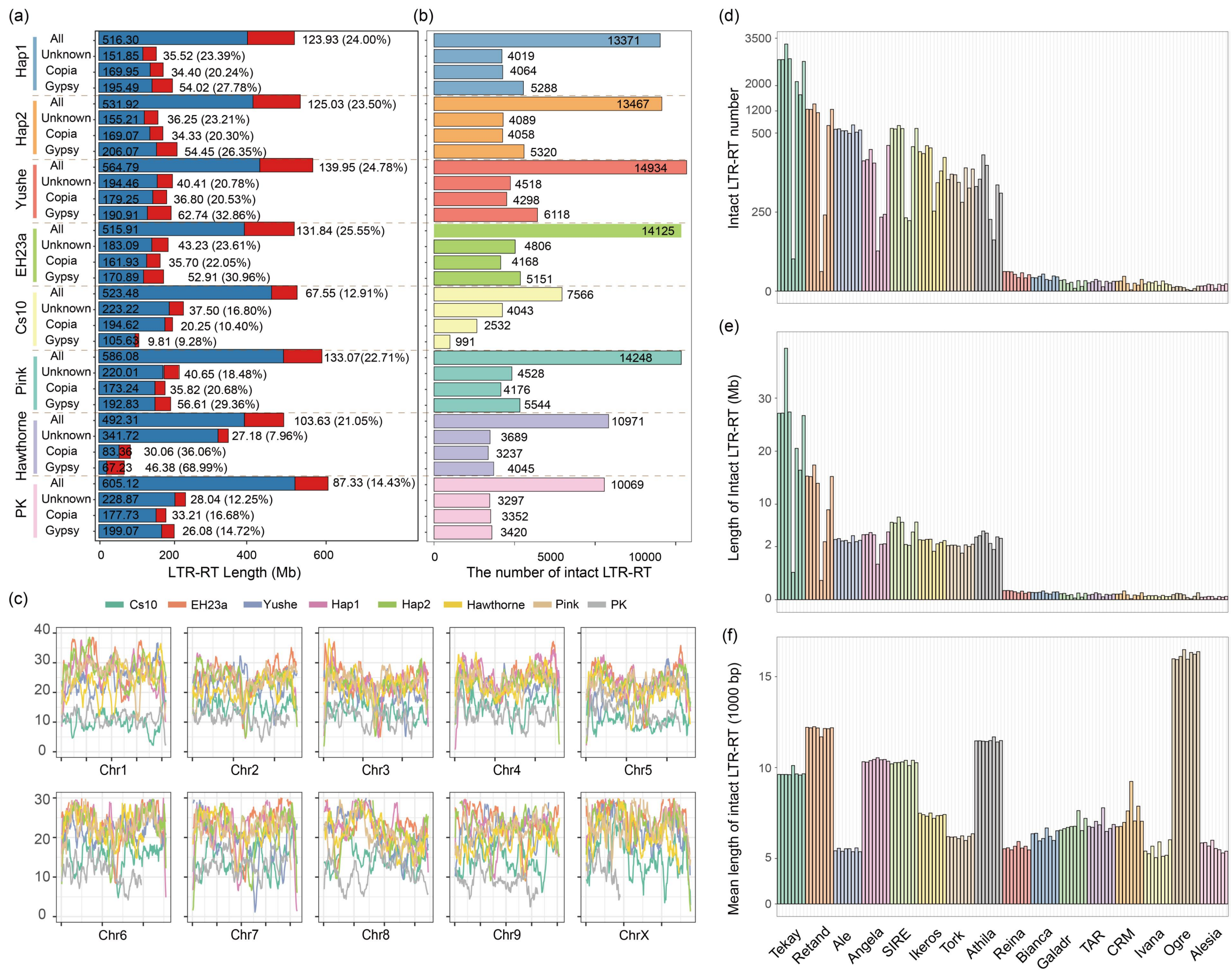

Supplementary FIGURE 14 | Comparative genomic characterization of intact LTR retrotransposons across eight *Cannabis* genomes. (a) Total lengths of intact (red) and raw (blue) LTR retrotransposons (LTR-RTs) across genomes. For each genome, LTR-RT length is shown for various categories including all LTRs, unknown LTRs, Copia LTRs, and Gypsy LTRs. The number within the blue bars indicates the total (raw) LTR-RT length, and the number to the right of each bar indicates the length of intact LTRs. The value in parentheses indicates the ratio of intact to total LTR-RT length. Different genome assemblies are represented by bars in distinct colors. (b) Distribution of intact LTR-RT counts across different genomes. Categories include total intact LTRs, unknown intact LTRs, intact Copia LTRs, and intact Gypsy LTRs. Numbers to the right of each bar indicate the count within each category. Genome assemblies are represented by different colors. (c) Genome-wide chromosomal distribution of LAI values for eight *C. sativa* genomes. LAI was calculated using a 3 Mb window with a 300 kb step size. Chromosomes are aligned based on homologous groups defined by the GCS reference, and original chromosome numbering from other assemblies is ignored. Different line colors represent different *C. sativa* assemblies. (d-f**),** Distribution of intact LTR-RT elements by subfamily across the eight genomes (ordered from left to right within each subfamily: Hap1, Hap2, Yushe, Pink, Cs10, PK, Hawthorne, EH23a). Different colors represent distinct LTR subfamilies. Panel (d**)** shows the number of intact elements per subfamily; due to large differences in copy number, y-axis ranges differ among subfamilies (250-300, 800-1200, and 1200-2000). The panel (e) displays the total length of intact LTRs per subfamily. Panel (f) shows the average length of intact LTRs per subfamily. Missing bars indicate the absence of the corresponding subfamily in a given genome.

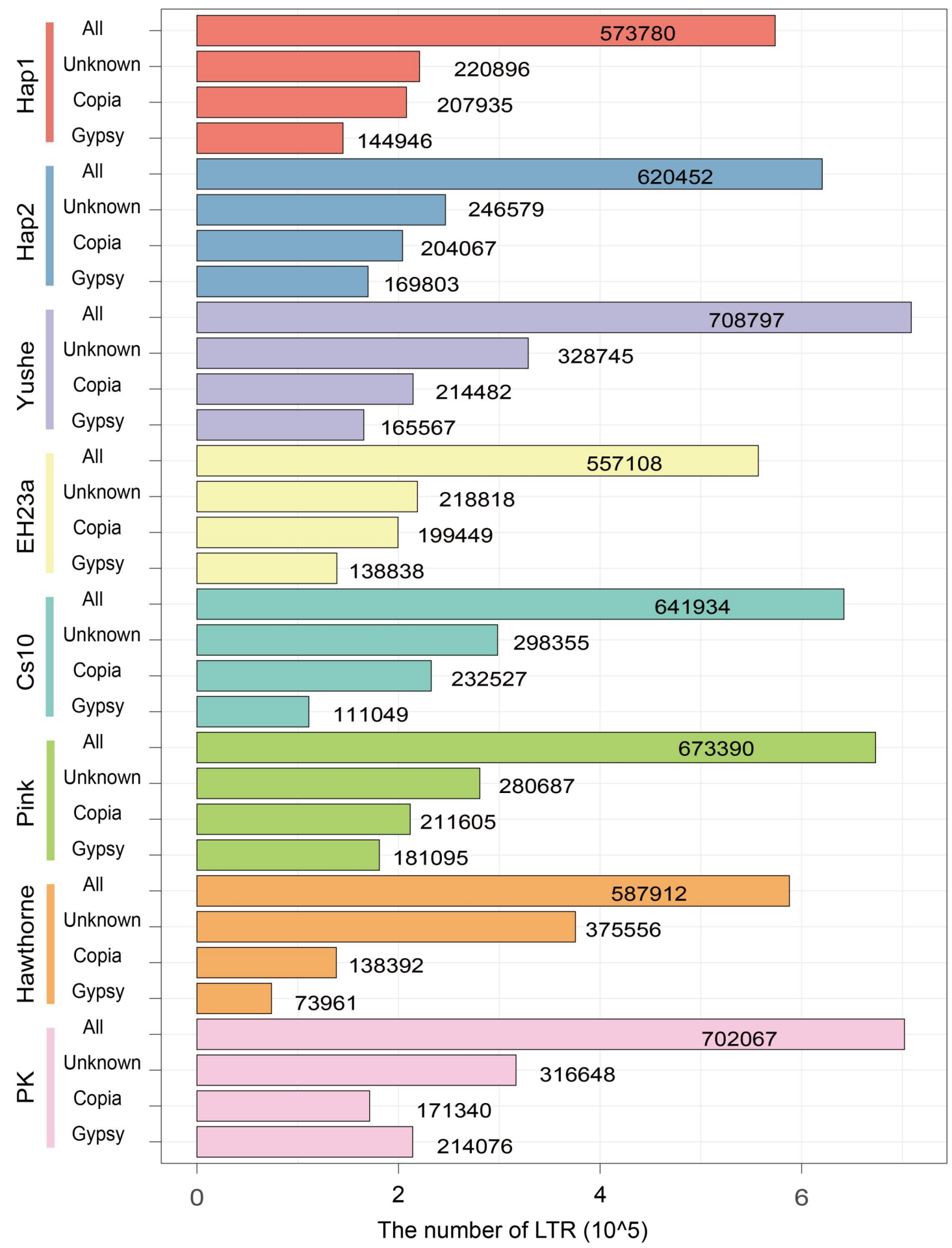

Supplementary FIGURE 15 | Variation in the number of LTR retrotransposons across *Cannabis sativa* genomes. Bar plots depicting the total number of raw LTR-RT annotated in eight *Cannabis sativa* genomes: Hap1, Hap2, Yushe, EH23a, Cs10, Pink Hawthorne, and PK. For each genome, separate bars show the total number of LTRs ("All") and the counts for unclassified (Unknown), Copia, and Gypsy subfamilies. Genomes are color-coded based on haplotype or variety, with total LTR counts (All) indicated at the end of each bar.

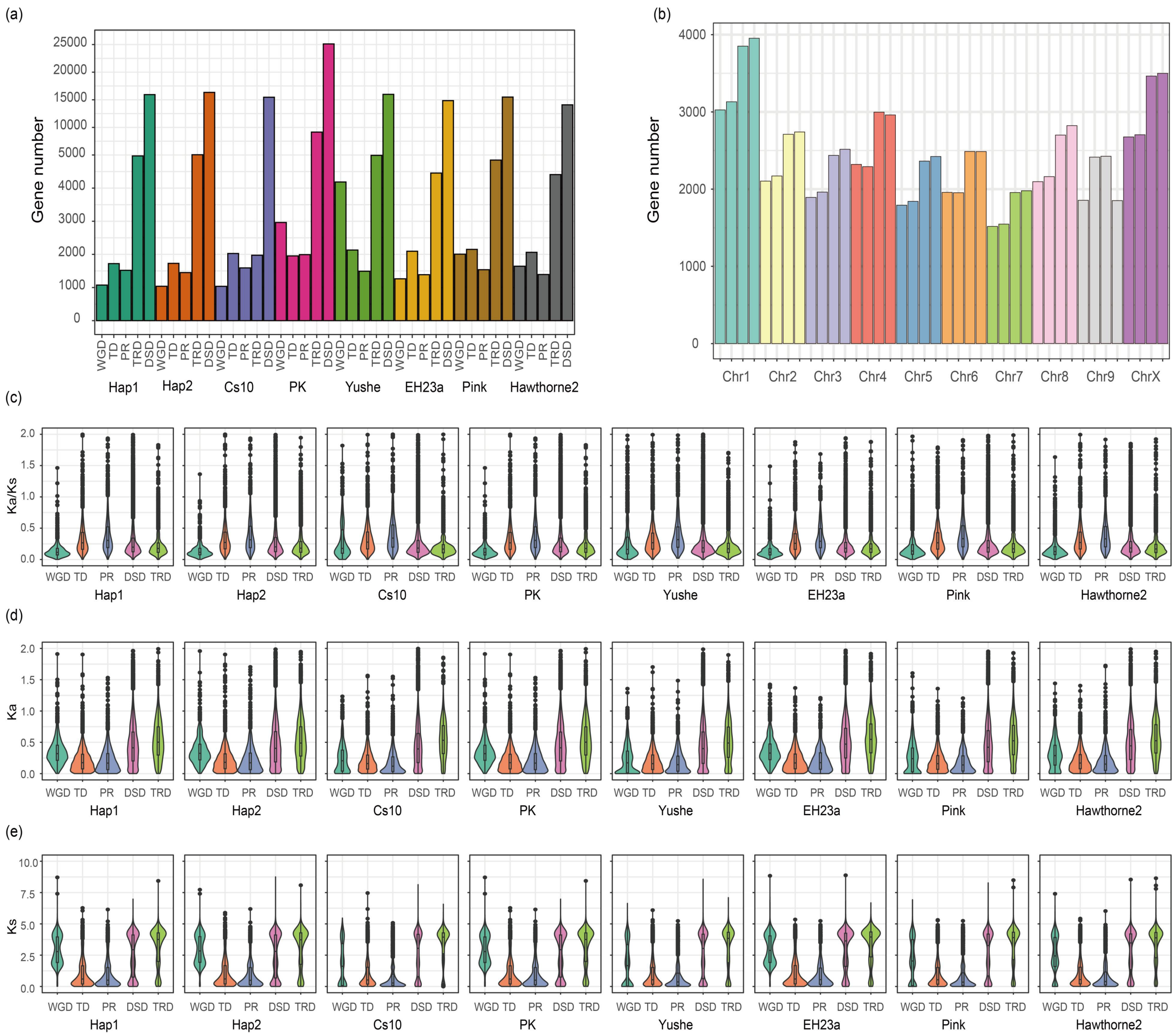

Supplementary FIGURE 16 | Gene duplication patterns and chromosomal homology across different *Cannabis sativa* genomes. (a) Counts of genes assigned to whole-genome duplication (WGD), tandem duplication (TD), proximal duplication (PR), dispersed duplication (DSD), and transposed duplication (TRD) in Hap1, Hap2, Cs10, PK, Yushe, EH23a, Pink and Hawthorne2 genomes. Bars are color-coded by assembly. (b) Gene numbers for homologous chromosome pairs of the two GCS haplotypes. Within each chromosome-specific color group, four adjacent bars represent Hap1 homologous genes, Hap2 homologous genes, Hap1 genes, and Hap2 genes from left to right. (c-e) Violin plots showing, for the five duplication categories in each genome, the distributions of Ka/Ks ratios (c), Ka values (d), and Ks values (e). Different duplication categories are color-coded, and each genome is displayed in a separate plot.

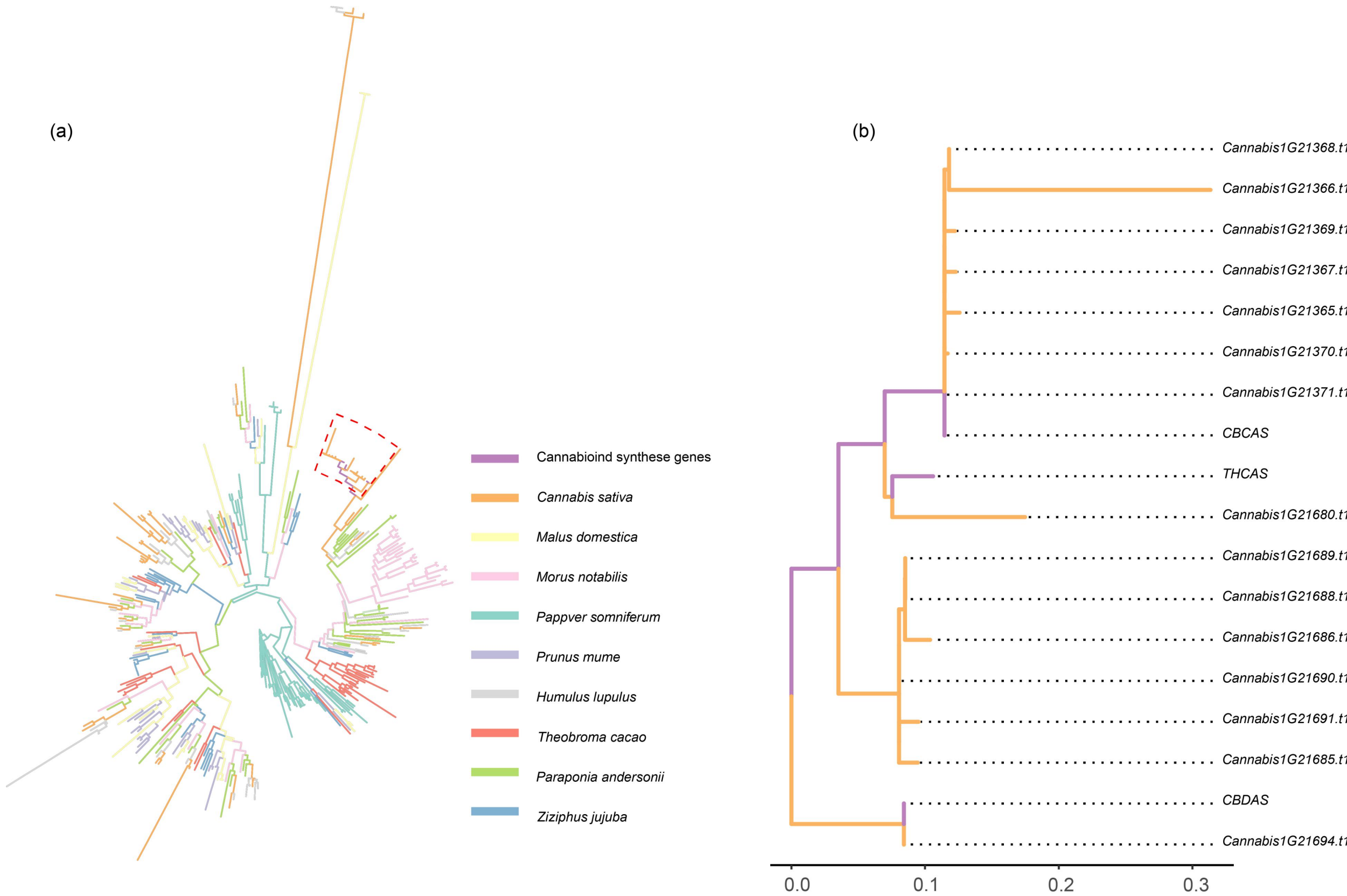

Supplementary FIGURE 17 | Phylogenetic tree of homologues of *CBDAS*, *THCAS* and *CBCAS* across representative plant species. (a) Maximum-likelihood phylogeny of protein sequences homologous to *CBDAS*, *THCAS* and *CBCAS* from representative plant species. Branches are colored by species. The red dashed box indicates the clade containing cannabinoid synthase homologus genes.. (b), Subtree corresponding to the clade in (a), with gene identifiers shown for *CBDAS*, *THCAS*, *CBCAS*, and related homologues from *Cannabis sativa*.

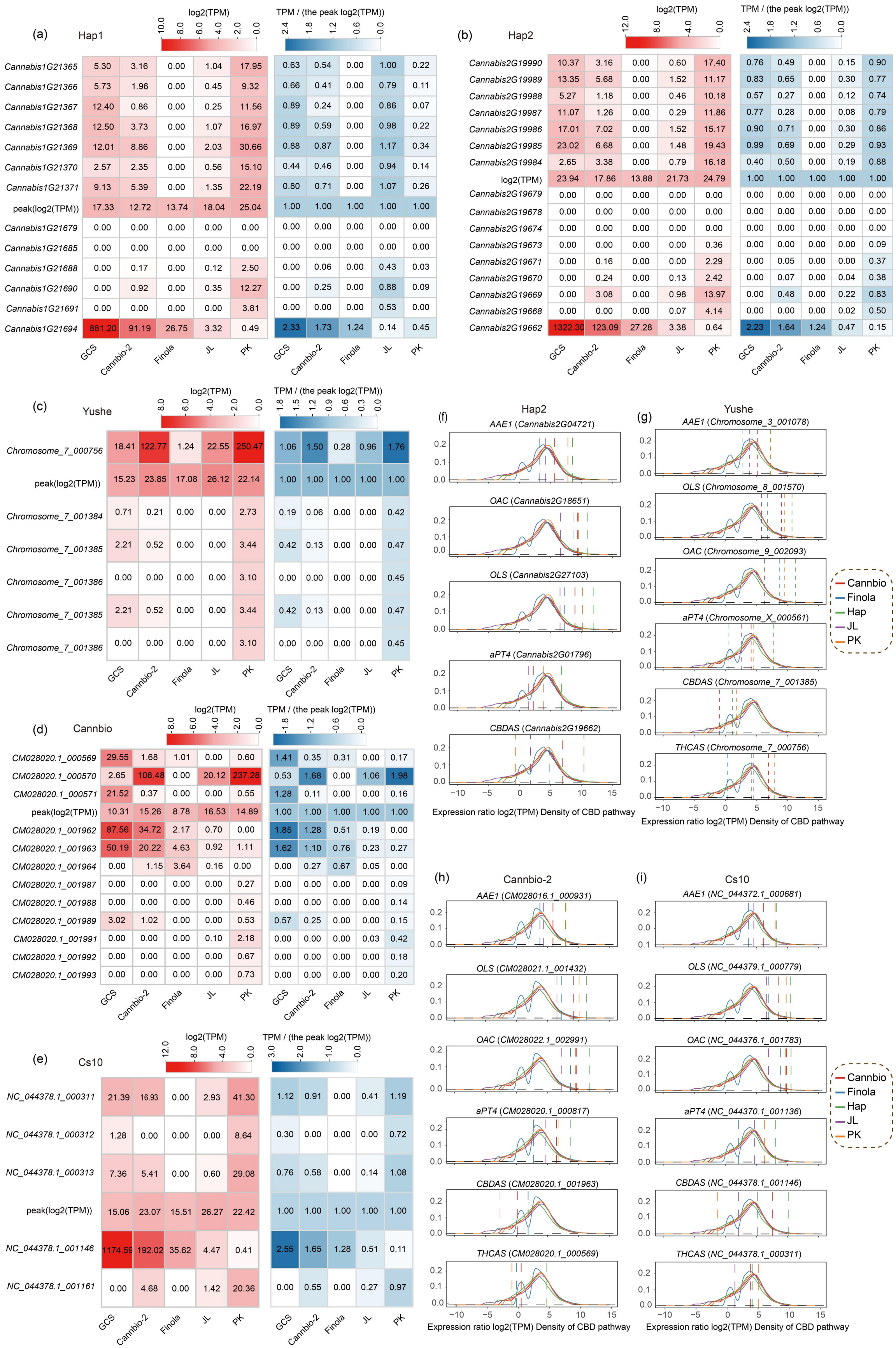

(a) Hap1
log2(TPM)
10.0 8.0 6.0 4.0 2.0 0.0
TPM / (the peak log2(TPM))
2.4 1.8 1.2 0.6 0.0
Cannabis1G21365 5.30 3.16 0.00 1.04 17.95 0.63 0.54 0.00 1.00 0.22
Cannabis1G21366 5.73 1.96 0.00 0.45 9.32 0.66 0.41 0.00 0.79 0.11
Cannabis1G21367 12.40 0.86 0.00 0.25 11.56 0.89 0.24 0.00 0.86 0.07
Cannabis1G21368 12.50 3.73 0.00 1.07 16.97 0.89 0.59 0.00 0.98 0.22
Cannabis1G21369 12.01 8.86 0.00 2.03 30.66 0.88 0.87 0.00 1.17 0.34
Cannabis1G21370 2.57 2.35 0.00 0.56 15.10 0.44 0.46 0.00 0.94 0.14
Cannabis1G21371 9.13 5.39 0.00 1.35 22.19 0.80 0.71 0.00 1.07 0.26
peak(log2(TPM)) 17.33 12.72 13.74 18.04 25.04 1.00 1.00 1.00 1.00 1.00
Cannabis1G21679 0.00 0.00 0.00 0.00 0.00 0.00 0.00 0.00 0.00 0.00
Cannabis1G21685 0.00 0.00 0.00 0.00 0.00 0.00 0.00 0.00 0.00 0.00
Cannabis1G21688 0.00 0.17 0.00 0.12 2.50 0.00 0.06 0.00 0.43 0.03
Cannabis1G21690 0.00 0.92 0.00 0.35 12.27 0.00 0.25 0.00 0.88 0.09
Cannabis1G21691 0.00 0.00 0.00 0.00 3.81 0.00 0.00 0.00 0.53 0.00
Cannabis1G21694 881.20 91.19 26.75 3.32 0.49 2.33 1.73 1.24 0.14 0.45
GCS Cannbio-2 Finola JL PK GCS Cannbio-2 Finola JL PK
(b) Hap2
log2(TPM)
12.0 8.0 4.0 0.0
TPM / (the peak log2(TPM))
2.4 1.8 1.2 0.6 0.0
Cannabis2G19990 10.37 3.16 0.00 0.60 17.40 0.76 0.49 0.00 0.15 0.90
Cannabis2G19989 13.35 5.68 0.00 1.52 11.17 0.83 0.65 0.00 0.30 0.77
Cannabis2G19988 5.27 1.18 0.00 0.46 10.18 0.57 0.27 0.00 0.12 0.74
Cannabis2G19987 11.07 1.26 0.00 0.29 11.86 0.77 0.28 0.00 0.08 0.79
Cannabis2G19986 17.01 7.02 0.00 1.52 15.17 0.90 0.71 0.00 0.30 0.86
Cannabis2G19985 23.02 6.68 0.00 1.48 19.43 0.99 0.69 0.00 0.29 0.93
Cannabis2G19984 2.65 3.38 0.00 0.79 16.18 0.40 0.50 0.00 0.19 0.88
log2(TPM) 23.94 17.86 13.88 21.73 24.79 1.00 1.00 1.00 1.00 1.00
Cannabis2G19679 0.00 0.00 0.00 0.00 0.00 0.00 0.00 0.00 0.00 0.00
Cannabis2G19678 0.00 0.00 0.00 0.00 0.00 0.00 0.00 0.00 0.00 0.00
Cannabis2G19674 0.00 0.00 0.00 0.00 0.00 0.00 0.00 0.00 0.00 0.00
Cannabis2G19673 0.00 0.00 0.00 0.00 0.36 0.00 0.00 0.00 0.00 0.09
Cannabis2G19671 0.00 0.16 0.00 0.00 2.29 0.00 0.05 0.00 0.00 0.37
Cannabis2G19670 0.00 0.24 0.00 0.13 2.42 0.00 0.07 0.00 0.04 0.38
Cannabis2G19669 0.00 3.08 0.00 0.98 13.97 0.00 0.48 0.00 0.22 0.83
Cannabis2G19668 0.00 0.00 0.00 0.07 4.14 0.00 0.00 0.00 0.02 0.50
Cannabis2G19662 1322.30 123.09 27.28 3.38 0.64 2.23 1.64 1.24 0.47 0.15
GCS Cannbio-2 Finola JL PK GCS Cannbio-2 Finola JL PK
(c) Yushe
log2(TPM)
8.0 6.0 4.0 2.0 0.0
TPM / (the peak log2(TPM))
1.8 1.5 1.2 0.9 0.6 0.3 0.0
Chromosome_7_000756 18.41 122.77 1.24 22.55 250.47 1.06 1.50 0.28 0.96 1.76
peak(log2(TPM)) 15.23 23.85 17.08 26.12 22.14 1.00 1.00 1.00 1.00 1.00
Chromosome_7_001384 0.71 0.21 0.00 0.00 2.73 0.19 0.06 0.00 0.00 0.42
Chromosome_7_001385 2.21 0.52 0.00 0.00 3.44 0.42 0.13 0.00 0.00 0.47
Chromosome_7_001386 0.00 0.00 0.00 0.00 3.10 0.00 0.00 0.00 0.00 0.45
Chromosome_7_001385 2.21 0.52 0.00 0.00 3.44 0.42 0.13 0.00 0.00 0.47
Chromosome_7_001386 0.00 0.00 0.00 0.00 3.10 0.00 0.00 0.00 0.00 0.45
GCS Cannbio-2 Finola JL PK GCS Cannbio-2 Finola JL PK
(d) Cannbio
log2(TPM)
8.0 6.0 4.0 2.0 0.0
TPM / (the peak log2(TPM))
1.8 1.2 0.6 0.0
CM028020.1_000569 29.55 1.68 1.01 0.00 0.60 1.41 0.35 0.31 0.00 0.17
CM028020.1_000570 2.65 106.48 0.00 20.12 237.28 0.53 1.68 0.00 1.06 1.98
CM028020.1_000571 21.52 0.37 0.00 0.00 0.55 1.28 0.11 0.00 0.00 0.16
peak(log2(TPM)) 10.31 15.26 8.78 16.53 14.89 1.00 1.00 1.00 1.00 1.00
CM028020.1_001962 87.56 34.72 2.17 0.70 0.00 1.85 1.28 0.51 0.19 0.00
CM028020.1_001963 50.19 20.22 4.63 0.92 1.11 1.62 1.10 0.76 0.23 0.27
CM028020.1_001964 0.00 1.15 3.64 0.16 0.00 0.00 0.27 0.67 0.05 0.00
CM028020.1_001987 0.00 0.00 0.00 0.00 0.27 0.00 0.00 0.00 0.00 0.09
CM028020.1_001988 0.00 0.00 0.00 0.00 0.46 0.00 0.00 0.00 0.00 0.14
CM028020.1_001989 3.02 1.02 0.00 0.00 0.53 0.57 0.25 0.00 0.00 0.15
CM028020.1_001991 0.00 0.00 0.00 0.10 2.18 0.00 0.00 0.00 0.03 0.42
CM028020.1_001992 0.00 0.00 0.00 0.00 0.67 0.00 0.00 0.00 0.00 0.18
CM028020.1_001993 0.00 0.00 0.00 0.00 0.73 0.00 0.00 0.00 0.00 0.20
GCS Cannbio-2 Finola JL PK GCS Cannbio-2 Finola JL PK
(e) Cs10
log2(TPM)
12.0 8.0 4.0 0.0
TPM / (the peak log2(TPM))
3.0 2.0 1.0 0.0
NC_044378.1_000311 21.39 16.93 0.00 2.93 41.30 1.12 0.91 0.00 0.41 1.19
NC_044378.1_000312 1.28 0.00 0.00 0.00 8.64 0.30 0.00 0.00 0.00 0.72
NC_044378.1_000313 7.36 5.41 0.00 0.60 29.08 0.76 0.58 0.00 0.14 1.08
peak(log2(TPM)) 15.06 23.07 15.51 26.27 22.42 1.00 1.00 1.00 1.00 1.00
NC_044378.1_001146 1174.59 192.02 35.62 4.47 0.41 2.55 1.65 1.28 0.51 0.11
NC_044378.1_001161 0.00 4.68 0.00 1.42 20.36 0.00 0.55 0.00 0.27 0.97
GCS Cannbio-2 Finola JL PK GCS Cannbio-2 Finola JL PK
(f) Hap2
AAE1 (Cannabis2G04721)
OAC (Cannabis2G18651)
OLS (Cannabis2G27103)
aPT4 (Cannabis2G01796)
CBDAS (Cannabis2G19662)
Expression ratio log2(TPM) Density of CBD pathway
(g) Yushe
AAE1 (Chromosome_3_001078)
OLS (Chromosome_8_001570)
OAC (Chromosome_9_002093)
aPT4 (Chromosome_X_000561)
CBDAS (Chromosome_7_001385)
THCAS (Chromosome_7_000756)
Expression ratio log2(TPM) Density of CBD pathway
Cannbio
Finola
Hap
JL
PK
(h) Cannbio-2
AAE1 (CM028016.1_000931)
OLS (CM028021.1_001432)
OAC (CM028022.1_002991)
aPT4 (CM028020.1_000817)
CBDAS (CM028020.1_001963)
THCAS (CM028020.1_000569)
Expression ratio log2(TPM) Density of CBD pathway
(i) Cs10
AAE1 (NC_044372.1_000681)
OLS (NC_044379.1_000779)
OAC (NC_044376.1_001783)
aPT4 (NC_044370.1_001136)
CBDAS (NC_044378.1_001146)
THCAS (NC_044378.1_000311)
Expression ratio log2(TPM) Density of CBD pathway
Cannbio
Finola
Hap
JL
PK

Supplementary FIGURE 18 | Expression profiles of cannabinoid synthase genes and associated pathways across different *Cannabis sativa* genomes. (a-e), Heat maps of log2(TPM) expression for homologous genes in the *CBDAS* and *THCAS/CBCAS* regions using the Hap1 (a), Hap2 (b), Yushe (c), Cannbio-2 (d) and Cs10 (e) as reference assemblies. Reference-assembly IDs are shown above each matrix. Accession IDs are plotted at the bottom of each corresponding column. Gene IDs are listed on the left. The row labelled "log2(TPM)" gives the TPM value at the peak of the kernel-density curve of log2(TPM) for all genes in that accession. Rows above "log2(TPM)" contain homologous genes from the *THCAS/CBCAS* region, whereas rows below contain homologous genes from the *CBDAS* region. Cell is colored according to log2(TPM), from white (low) to red (high). The heat map to the right converts each gene's TPM value to a ratio relative to the TPM at the "log2(TPM)" peak, with values colored from white (low) to blue (high). (f–i)**,** Kernel-density plots of log2(TPM) for key cannabinoid-biosynthesis genes using Hap2, Yushe, Cannbio-2 and Cs10 as reference assemblies (Hap2 lacks *THCAS* gene). Each gene-expression panel shows density curves of log2(TPM) values for all accessions, with the log2(TPM) value of that gene in each accession indicated by a vertical dashed line; curves and markers are color-coded by accession. Comparing these values with the peak positions provides an indirect measure of relative expression among accessions.

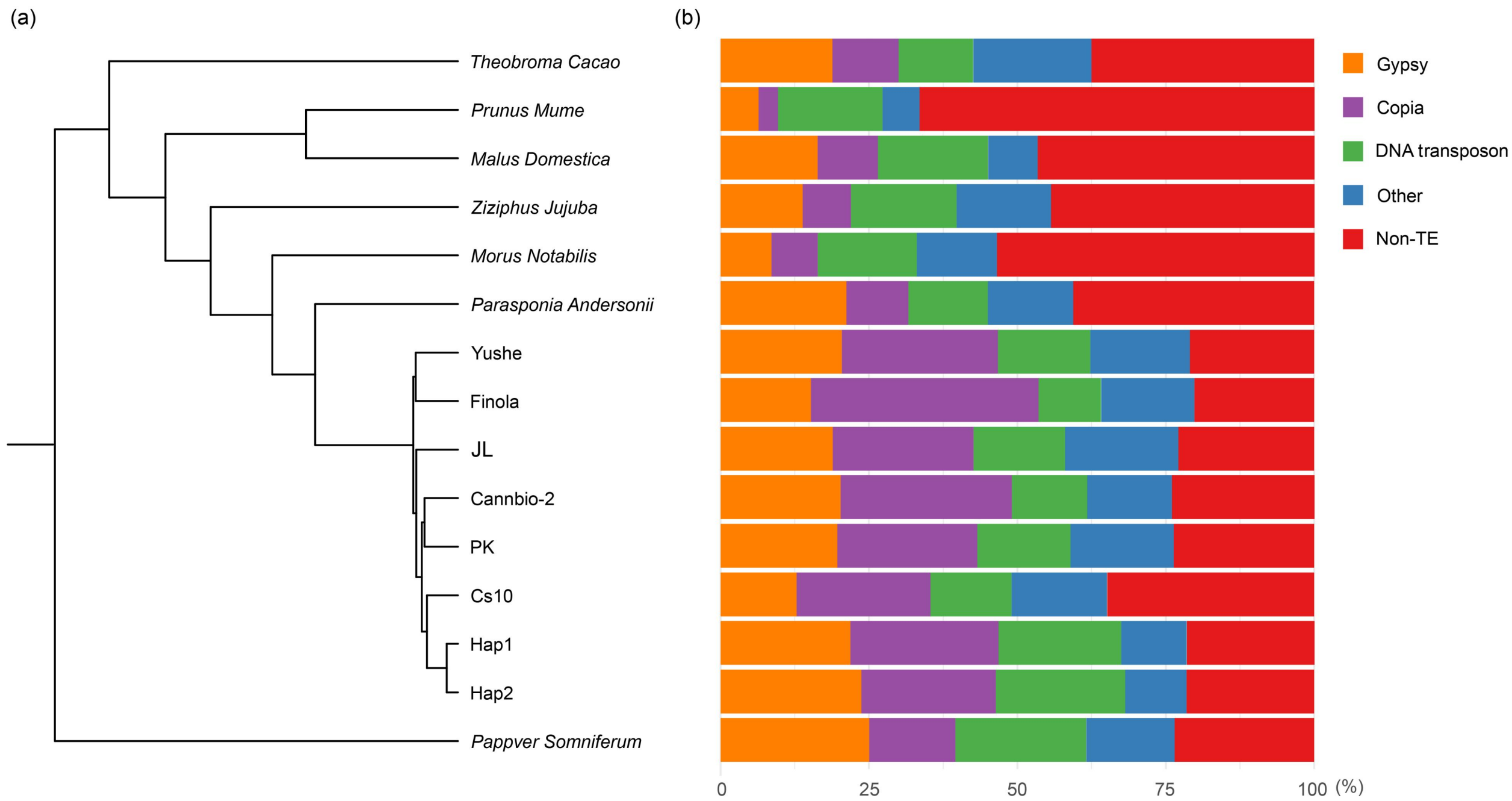

Supplementary FIGURE 19 | Phylogenetic relationships and repeat element compositions among *Cannabis sativa* genomes and related species. (a) A maximum-likelihood phylogenetic tree was constructed from 16 single-copy orthologous genes for 15 plant genomes, including eight *C. sativa* assemblies (Hap1, Hap2, Cs10, PK, Cannbio-2, JL, Finola, and Yushe) and seven other species (*Parasponia andersonii*, *Morus notabilis*, *Ziziphus jujuba*, *Malus domestica*, *Prunus mume*, *Theobroma cacao*, and *Papaver somniferum*), with *Papaver somniferum* used as the outgroup. Within *C. sativa*, Hap1, Hap2, and Cs10 represent cannabidiol (CBDA)-producing cultivars and form a tight cluster; THCA-producing cultivars Cannbio-2 and PK group into a distinct clade; oilseed cultivar Yushe and fiber cultivar Finola cluster closely together, while JL, a wild-type accession, occupies an independent branch. (b) Stacked bar plots illustrating the relative abundance of major TE categories: Gypsy (orange), Copia (purple), DNA transposons (green), and other repeats (blue), together with non-transposable element (non-TE) sequences (red) in the gene-flanking regions of diverse plant genomes and *Cannabis* accessions.

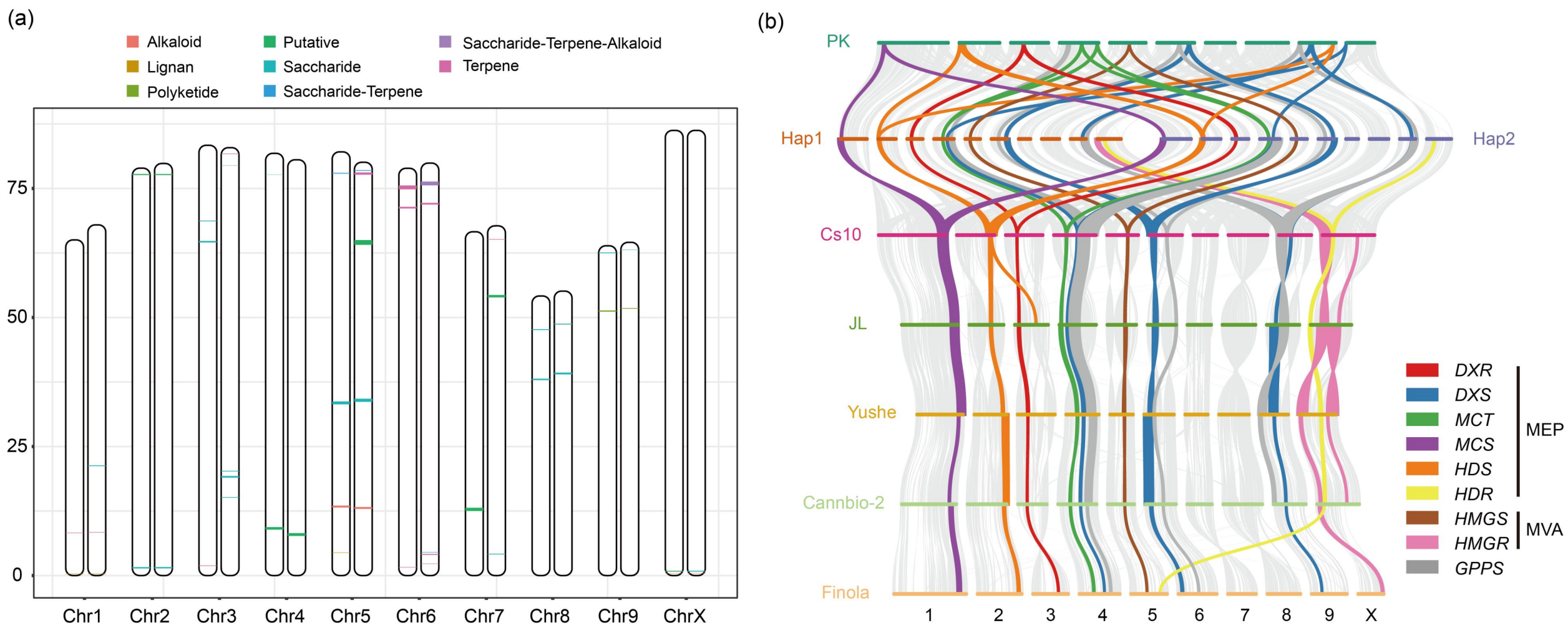

Supplementary FIGURE 20 | Distribution and conservation of secondary metabolite biosynthetic gene clusters in *Cannabis sativa* genomes. (a) Chromosomal distribution of secondary metabolite biosynthetic gene clusters in the two haplotype-phased genomes. Colored blocks denote gene clusters involved in distinct pathways, including alkaloid, lignan, polyketide, saccharide, terpene, and their combinatorial biosynthetic routes. In each chromosome pair, the left and right arms correspond to Hap1 and Hap2, respectively. (b) Synteny of geranyl pyrophosphate (GPP) biosynthetic pathway genes across eight *C. sativa* genomes. Ribbon links indicate conserved orthologs of core pathway genes, including *DXR*, *DXS*, *MCT*, *MCS*, *HDR*, *HDS*, *IDI*, *HMGR*, *HMGS*, and *GPPS*, revealing both shared and lineage-specific retention patterns among cultivars and haplotypes. GCS is shown as two phased haploid assemblies to preserve haplotype-specific synteny, whereas the other accessions are shown as single collapsed or chimeric assemblies.

Part IV: Tables

Table S1. Summary statistics of *Cannabis* genome generated GCS sequencing data used in the genome assembly

Table S2. Evaluation of genome size and heterozygosity

Table S3. Number of gaps per chromosome in the scaffolded GCS haplotype genomes

Table S4. Summary statistics for scaffolding and gap-filling of the two GCS haplotype genomes

Table S5. Summary of the two haplotype genomes

Table S6. Evaluation of genome completeness using BUSCO

Table S7. Chromosome-wise distribution of duplicated BUSCOs in the two haplotype assemblies

Table S8. Summary of telomeric arrays at chromosome ends in the two haplotypes

Table S9. Genomic distribution and characteristics of 45S and 5S rDNA arrays in the haplotype-phased *Cannabis sativa* assemblies

Table S10. Haplotype-phased chromosome-level statistics for 45S/5S rDNA array counts and lengths

Table S11. Statistics of the HiFi reads mapped to the two haplotype genomes

Table S12. Statistics of the CLR reads mapped to the two haplotype genomes

Table S13. Statistics of the NGS reads mapped to the two haplotype genomes

Table S14. Chromosome-level HiFi coverage of the two haplotype assemblies: median read depth and normalized coverage

Table S15. HiFi reads-based k-mer estimates for chromosomes of two haplotype genomes

Table S16. HiFi reads-based estimates for the two *Cannabis sativa* haplotype genomes

Table S17. Comparative analysis of assembly metrics across diverse *Cannabis* genomes

Table S18. Intact LTR-RT content and LAI in two haplotype genomes

Table S19. Haplotype-specific genes and one-to-one allelic gene pairs between the two haplotype genomes

Table S20. Distribution of haplotype-specific (unique) genes across chromosomes in the two haplotype genomes

Table S21. Genes with rapidly changing copy numbers

Table S22. Summary of repetitive sequences, duplicated genes and lengths of repetitive, genic and intergenic regions on different chromosomes

Table S23. Syntenic Depth Distribution Between *Cannabis sativa* and *Humulus japonicus*

Table S24. Syntenic classification of Non-1:1 gene pairs between *Cannabis sativa* and *Humulus japonicus* (tandem vs non-tandem)

Table S25. Raw and intact LTR-RTs numbers across different *Cannabis sativa* genomes

Table S26. Raw and intact LTR-RT lengths in different *Cannabis sativa* genomes

Table S27. Numbers of various duplicated genes in different *Cannabis sativa* genomes

Table S28. HMMER-detected *CBDAS*, *THCAS* and *CBCAS* homologues showing amino acid residues at positions corresponding to His292 and Tyr417

Table S29. Positively selected genes in Cannabis sativa

Table S30. OGs of the cannabinoid pathway in seven Cannabis genomes and the number of PAV insertions

Table S31. Transposable element insertion counts in intronic and 2-kb flanking regions, and corresponding expression levels of *CBDAS*, *THCAS* and *CBCAS* across seven Cannabis sativa accessions

Table S32. Expression of *CBDAS*/*THCAS* tandemly duplicated genes in the Hap1 reference genome

Table S33. Expression of *CBDAS*/*THCAS* tandemly duplicated genes in the Hap2 reference genome

Table S34. Expression of *CBDAS*/*THCAS* tandemly duplicated genes in the Yushe reference genome

Table S35. Expression of *CBDAS*/*THCAS* tandemly duplicated genes in the Cannbio-2 reference genome

Table S36. Expression of *CBDAS*/*THCAS* tandemly duplicated genes in the Cs10 reference genome

Table S37. TE ratio of eight *Cannabis sativa* genomes and seven related species

Table S38. Distribution of secondary metabolite biosynthetic gene clusters in the two haplotype-phased GCS genomes

Tables S1 to S38: full Tables are provided in the Excel file:

Cannabis_Supplementary_table_S1-S38.xlsx

References

Buchfink, B., Reuter, K., and Drost, H.G. (2021) *Sensitive protein alignments at tree-of-life scale using DIAMOND*. *Nature Methods*.

Chen, S., Zhou, Y., Chen, Y., and Gu, J. (2018) *Fastp: An ultra-fast all-in-one FASTQ preprocessor*. In: *Bioinformatics*.

Danecek, P., Bonfield, J.K., Liddle, J., Marshall, J., Ohan, V., Pollard, M.O., et al. (2021) *Twelve years of SAMtools and BCFtools*. *GigaScience*.

Gurevich, A., Saveliev, V., Vyahhi, N., and Tesler, G. (2013) *QUAST : quality assessment tool for genome assemblies*. **29**, 1072–1075.

He, W., Yang, J., Jing, Y., Xu, L., Yu, K., and Fang, X. (2023) *NGenomeSyn: an easy-to-use and flexible tool for publication-ready visualization of syntenic relationships across multiple genomes*. *Bioinformatics*.

Jombart, T. (2008) *Adegenet: A R package for the multivariate analysis of genetic markers*. *Bioinformatics*.

Katoh, K. and Standley, D.M. (2013) *MAFFT multiple sequence alignment software version 7: Improvements in performance and usability*. *Molecular Biology and Evolution*.

Kautsar, S.A., Suarez Duran, H.G., Blin, K., Osbourn, A., and Medema, M.H. (2017) *PlantiSMASH: Automated identification, annotation and expression analysis of plant biosynthetic gene clusters*. *Nucleic Acids Research*, **45**.

Kendig, K.I., Baheti, S., Bockol, M.A., Drucker, T.M., Hart, S.N., Heldenbrand, J.R., et al. (2019) *SentIeon DNaSeq variant calling workflow demonstrates strong computational performance and accuracy*. *Frontiers in Genetics*.

Krueger, F. and Andrews, S.R. (2011) *Bismark: A flexible aligner and methylation caller for Bisulfite-Seq applications*. *Bioinformatics*.

Krzywinski, M., Schein, J., Birol, I., Connors, J., Gascoyne, R., Horsman, D., et al. (2009) *Circos: An information aesthetic for comparative genomics*. *Genome Research*.

Langmead, B. and Salzberg, S.L. (2012) *Fast gapped-read alignment with Bowtie 2*. *Nature Methods*.

Li, H. (2021) *New strategies to improve minimap2 alignment accuracy*. *Bioinformatics*.

Li, H. and Durbin, R. (2010) *Fast and accurate long-read alignment with Burrows-Wheeler transform*. *Bioinformatics*, **26**, 589–595.

Lynch, R.C., Padgitt-Cobb, L.K., Garfinkel, A.R., Knaus, B.J., Hartwick, N.T., Allsing, N., et al. (2025) *Domesticated cannabinoid synthases amid a wild mosaic cannabis pangenome*.

Marçais, G. and Kingsford, C. (2011) *A fast, lock-free approach for efficient parallel counting of occurrences of k-mers*. *Bioinformatics*.

Minh, B.Q., Schmidt, H.A., Chernomor, O., Schrempf, D., Woodhams, M.D., Von Haeseler, A., et al. (2020) *IQ-TREE 2: New Models and Efficient Methods for Phylogenetic Inference in the Genomic Era*. *Molecular Biology and Evolution*.

Ou, S., Chen, J., and Jiang, N. (2018) *Assessing genome assembly quality using the LTR Assembly Index (LAI)*. *Nucleic acids research*.

Quinlan, A.R. and Hall, I.M. (2010) *BEDTools: A flexible suite of utilities for comparing genomic features*. *Bioinformatics*.

R Core Team (2019) *R: A language and environment for statistical computing. R Foundation for Statistical Computing*.

Rambaut (2018) *FigTree. Tree figure drawing tool. http://tree.bio.ed.ac.uk/software/figtree/*.

Ramírez, F., Dündar, F., Diehl, S., Grüning, B.A., and Manke, T. (2014) *DeepTools: A flexible platform for exploring deep-sequencing data*. *Nucleic Acids Research*.

Ranallo-Benavidez, T.R., Jaron, K.S., and Schatz, M.C. (2020) *GenomeScope 2.0 and Smudgeplot for reference-free profiling of polyploid genomes*. *Nature Communications*.

dos Reis, M. and Yang, Z. (2019) *Bayesian molecular clock dating using genome-scale datasets*. In: *Methods in Molecular Biology*.

Rhie, A., Walenz, B.P., Koren, S., and Phillippy, A.M. (2020) *Merqury: Reference-free quality, completeness, and phasing assessment for genome assemblies*. *Genome Biology*, **21**, 1–27.

Robinson, J.T., Thorvaldsdottir, H., Turner, D., and Mesirov, J.P. (2023) *igv.js: an embeddable JavaScript implementation of the Integrative Genomics Viewer (IGV)*. *Bioinformatics*.

Shen, W., Le, S., Li, Y., and Hu, F. (2016) *SeqKit: A cross-platform and ultrafast toolkit for FASTA/Q file manipulation*. *PLoS ONE*.

Simão, F.A., Waterhouse, R.M., Ioannidis, P., Kriventseva, E. V., and Zdobnov, E.M. (2015) *BUSCO: Assessing genome assembly and annotation completeness with single-copy orthologs*. *Bioinformatics*.

Sun, H., DIng, J., Piednoël, M., and Schneeberger, K. (2018) *FindGSE: Estimating genome size variation within human and Arabidopsis using k -mer frequencies*. *Bioinformatics*.

Wang, Y., Tang, H., Wang, X., Sun, Y., Joseph, P. V., and Paterson, A.H. (2024) *Detection of colinear blocks and synteny and evolutionary analyses based on utilization of MCScanX*. *Nature Protocols*.

Xu, S., Li, L., Luo, X., Chen, M., Tang, W., Zhan, L., et al. (2022) *Ggtree: A serialized data object for visualization of a phylogenetic tree and annotation data*. *iMeta*.

Zhang, Y., Liu, T., Meyer, C.A., Eeckhoute, J., Johnson, D.S., Bernstein, B.E., et al. (2008) *Model-based analysis of ChIP-Seq (MACS)*. *Genome Biology*.
